# Admission ionised calcium disturbances and adverse outcomes in paediatric major trauma: a UK multicentre retrospective cohort study

**DOI:** 10.1136/bmjopen-2026-122447

**Published:** 2026-09-18

**Authors:** Owen Hibberd, Edward B G Barnard, Spyridon Karageorgos, Damian Roland, Tim Harris, Stephen H Thomas, Paediatric Emergency Research in the United Kingdom & Ireland (PERUKI), Nichola Anderson

**Affiliations:** 1 Emergency and Urgent Care Research in Cambridge (EURECA), PACE Section, Department of Medicine, University of Cambridge, Cambridge, UK; 2 Blizard Institute, Queen Mary University of London, London, UK; 3 Department of Research, Audit, Innovation, & Development (RAID), East Anglian Air Ambulance, Norwich, England, UK; 4 Academic Department of Military Emergency Medicine, Royal Centre for Defence Medicine (Research & Clinical Innovation), Birmingham, UK; 5 University of Crete School of Medicine, Heraklion, Greece; 6 Pediatric Emergency Department, Aghia Sophia Children's Hospital, Athens, Greece; 7 School of Medicine, European University Cyprus, Nicosia, Cyprus; 8 SAPPHIRE Group, Population Health Sciences, Leicester University, Leicester, UK; 9 Paediatric Emergency Medicine Leicester Academic (PEMLA) Group, Children’s Emergency Department, Leicester Royal Infirmary, Leicester, UK; 10 Department of Emergency Medicine, Harvard Medical School, Boston, Massachusetts, USA

**Keywords:** TRAUMA MANAGEMENT, Paediatric surgery, ACCIDENT & EMERGENCY MEDICINE, Calcium & bone

## Abstract

**Abstract:**

**Background:**

Calcium disturbances are increasingly recognised as detrimental in adult trauma patients; both ionised hypocalcaemia (iHypoCa) and ionised hypercalcaemia (iHyperCa) are associated with increased mortality. Paediatric data is limited and heterogeneous, with suggestions that iHypoCa may be associated with haemodynamic instability and increased treatment requirements. No paediatric studies have explored the effect of iHyperCa.

**Objectives:**

To examine the association between admission ionised calcium (iCa) disturbances and hypotension, treatment requirements, poor functional outcome and mortality in paediatric major trauma.

**Methods:**

A multicentre retrospective cohort study across 13 sites in the United Kingdom (2016–2023). Children aged <16 years with major trauma (Injury Severity Score≥15) and an admission iCa measurement were included. Calcium levels were categorised as iHypoCa (iCa<1.16 mmol/L), ionised normocalcaemia (iCa≥1.16 mmol/L–1.29 mmol/L) and iHyperCa (iCa≥1.30 mmol/L). The primary outcome was hypotension in the Emergency Department. Secondary outcomes included treatment requirements within 24 hours, poor functional outcome at 30 days (Glasgow Outcome Scale≤4), and 24-hour and 30-day mortality. Multivariable logistic regression adjusted for injury severity, age categories, mechanism and sex.

**Results:**

Among 517 children, 102/517 (19.7%) had iHypoCa and 72/517 (13.9%) iHyperCa on admission. iHypoCa was associated with hypotension and coagulation abnormalities on univariable analysis but was not independently associated with treatment requirements, poor functional outcome or mortality after adjustment. In contrast, iHyperCa was associated with a high-risk phenotype and was independently associated with poor functional outcome (OR 2.52, 95% CI 1.45 to 4.35), 24-hour mortality (OR 19.33, 95% CI 6.95 to 53.69) and 30-day mortality (OR 9.07, 95% CI 4.92 to 16.72).

**Conclusions:**

Admission calcium disturbances are prevalent in paediatric major trauma. These findings support contextual interpretation of calcium levels, the need for caution with empiric calcium administration in injured children and highlight the potential value of admission iHyperCa as a marker of physiological stress in injured children.

**STRENGTHS AND LIMITATIONS OF THIS STUDY**

- Multicentre study including 13 sites across the Paediatric Emergency Research UK & Ireland (PERUKI) research network.
- Largest study to date evaluating both ionised hypocalcaemia and ionised hypercalcaemia in paediatric trauma.
- Multivariable regression adjusted for important demographic and injury characteristics.
- Retrospective observational design cannot establish causality.
- Convenience sampling may have introduced residual confounding.

## Introduction

 Trauma-associated mortality has traditionally been linked to the ‘lethal triad’ of acidosis, coagulopathy and hypothermia; more recently, this construct has evolved into the ‘diamond of death’ with the addition of ionised hypocalcaemia (iHypoCa).^1–4^ Ionised calcium (iCa) disturbances are thought to be detrimental in haemorrhage due to calcium’s role in clot formation, vascular tone and cardiac contractility. Proposed mechanisms for iHypoCa in trauma include, but are not limited to, direct calcium consumption and loss from coagulation and bleeding, haemodilution from the administration of intravenous fluids, calcium chelation through the transfusion of citrated blood products, calcium-lactate binding and intracellular calcium influx in the setting of ischaemia and reperfusion.^1 5^ Among adult trauma patients, early iHypoCa in the Emergency Department (ED) is estimated to occur in up to 50% of patients and is associated with shock, coagulopathy, an increase in subsequent blood transfusion requirements, and mortality.^6–11^ However, protocolised exogenous calcium replacement is complicated by variable practice, and adverse outcomes have also been observed in adult trauma patients with ionised hypercalcaemia (iHyperCa).^12–19^

Calcium disturbances in paediatric trauma represent an established research priority across organisations through the prioritisation of research into the management of traumatic haemorrhage, trauma risk stratification and trauma biomarkers.^20–25^ Children may also be particularly vulnerable to calcium disturbances compared with adults because of differences in haemodynamic response and physiological reserves, different injury mechanisms and patterns, higher bone turnover, smaller total calcium stores and developmental haemostasis referring to the age-related maturation of the coagulation system, whereby the concentrations and function of coagulation factors, anticoagulant proteins, fibrinolytic pathways and platelet activity differ between neonates, infants, children and adults.^26–30^ Compared with adults, evidence on the differential determinants and effects of calcium disturbances in paediatric trauma is limited to a small number of heterogeneous studies.^31–35^ These studies indicate that iHypoCa may be associated with higher odds of haemodynamic instability than ionised normocalcaemia (iNormoCa) and suggest associations with other adverse outcomes, such as the need for blood product transfusion, invasive management (in the form of interventional radiology (IR) or surgery) and poor functional outcomes.^32–35^ To date, no paediatric studies have examined the effects of iHyperCa in trauma.^31^ Given the importance of haemodynamic instability and early treatment in paediatric major trauma, further investigation in this area is likely to be clinically meaningful and may improve understanding and management of haemorrhage and shock in injured children. Moreover, a recent international survey of practices around the measurement and replacement of calcium in paediatric major trauma demonstrated substantial variation in both the measurement and replacement of iCa, highlighting a lack of consensus and a need for further evidence.^17^

## Objectives

This study aimed to report on the association between iCa disturbances and hypotension in a large cohort of paediatric major trauma patients. The study also aimed to report on the incidence of iCa disturbances and their associations with adverse outcomes, including physiological derangements and treatment requirements in the first 24 hours, as well as poor functional outcomes and mortality.

## Methods

### Study design

**Figure 1:**
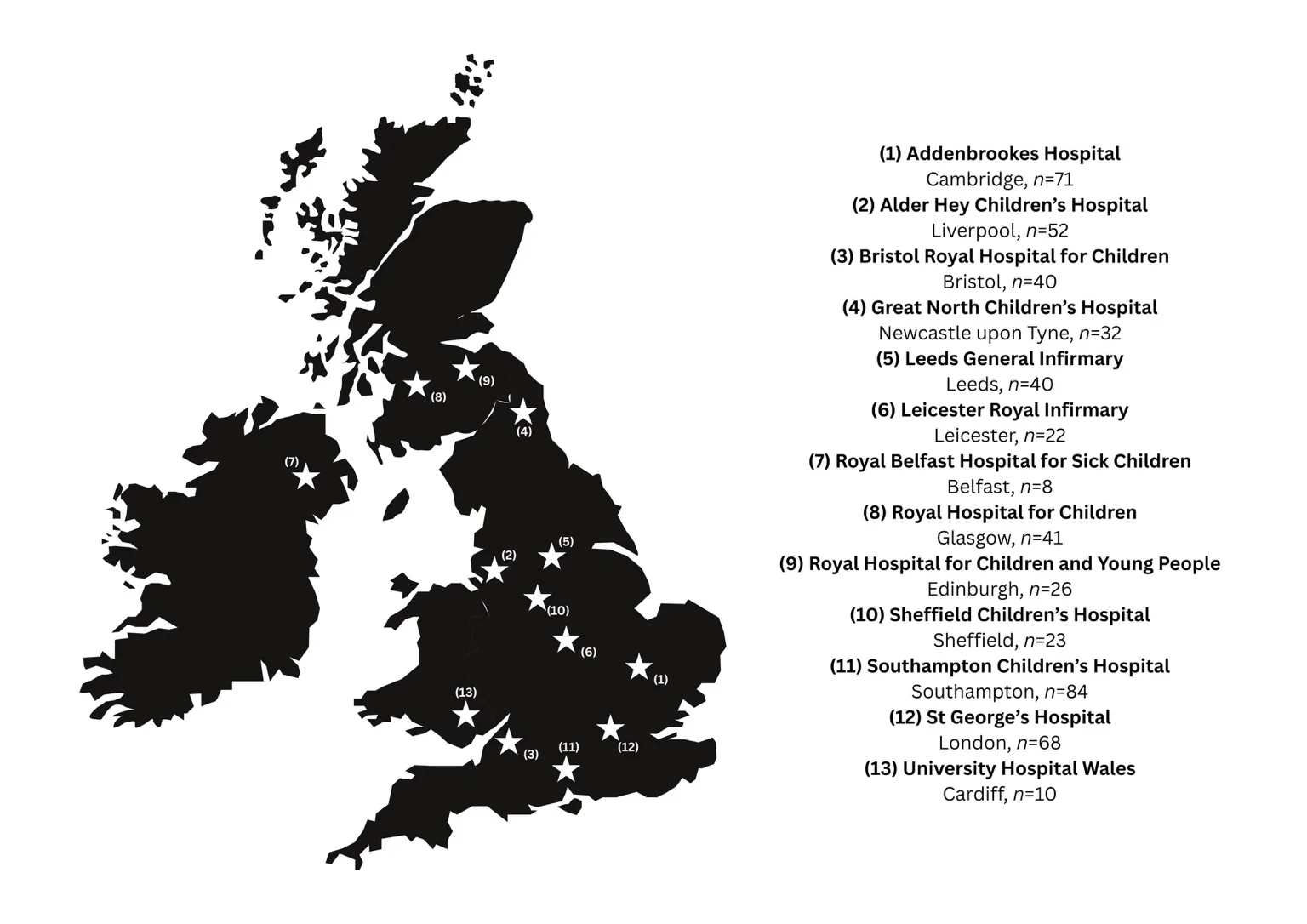
Location of participating sites.

This multicentre retrospective cohort study was undertaken in collaboration with the national research network *Paediatric Emergency Research UK and Ireland (PERUKI*) across 13 sites (figure 1). The study followed the *Strengthening the Reporting of Observational Studies in Epidemiology (STROBE*) guideline (online supplemental appendix 1).^36^ Data between August 2016 and August 2023 were included. The start date is a few years after the commissioning of the major trauma network, allowing for the maturity of the networks and the more widespread introduction of electronic medical records, while the end date was linked to the closure of the *Trauma Audit and Research Network (TARN*) data registry.^37^

### Participants

A convenience sample of children with an iCa level measured on admission was included if they were <16 years old, had an Injury Severity Score (ISS) of ≥15, and were TARN/Scottish Trauma Audit Group (STAG) positive, defined as admission to hospital for 3 days or more, admission to intensive care, transfer for further specialist care or death. Patients were excluded if they received a blood product transfusion in the ED prior to iCa measurement. Patients transferred from another hospital following initial treatment for the same injury were excluded because transfer-related delays and incomplete access to data from the referring hospital could have confounded the analyses.^38^

### Outcomes

The primary outcome was hypotension (defined dichotomously using age-related values) and was measured as the lowest recorded systolic blood pressure during ED stay. This outcome was selected because of its hypothesised mechanistic relationship with calcium disturbances and its clinical relevance in the ED management of paediatric trauma, where mortality is relatively uncommon. Secondary outcomes included the need for exogenous calcium, vasoactive medication, blood product transfusion and IR/surgery within the first 24 hours of admission (all defined dichotomously), and hospital/paediatric intensive care unit (PICU) length of stay (LOS). Mortality within 24 hours and 30 days was also reported, as was poor functional outcome at 30 days (Glasgow Outcome Score (GOS)≤4).^39^

### Data collection

Age categories, sex, mechanism of injury, ISS, 30-day mortality and poor functional outcome were obtained from the local Trauma Office records. Hospital and PICU LOS data were obtained from the local Trauma Office records of participating centres. The presence of hypotension (systolic blood pressure of <80 mm Hg for <1 year, <85 mm Hg for 1–5 years, <90 mm Hg for 5–12 years, <100 mm Hg for >12 years; defined dichotomously based on standard reference values)^35 40^ and details of prehospital and hospital treatments (fluid administration, blood product transfusion, vasoactive agents, invasive management (IR or surgery)) were obtained manually from the electronic medical record (EMR) by local collaborators. Additionally, blood gas data, heart rate and systolic blood pressure were also manually extracted from the EMR. Levels of iCa were defined as iHypoCa (iCa<1.16 mmol/L) and severe iHypoCa (iCa<1 mmol/L),^31^ iNormoCa (iCa≥1.16 mmol/L–1.29 mmol/L) and iHyperCa (iCa≥1.30 mmol/L).^12^ Deranged clotting was defined dichotomously based on the presence of one or more of: raised prothrombin time (PT) ≥12.6 s for <1 year, >13.4 s for 1–5 years, >14.6 s for 6–10 years, >14.1 s for ≥11 years; raised activated partial thromboplastin time (APTT) ≥40.7 s for <1 year, >39.2 s for 1–5 years, >38.7 s for 6–10 years, >38.4 s for ≥11 years; low fibrinogen≤1.41 g/L for <1 year, <1.64 g/L for 1–5 years, <1.71 g/L for 6–10 years, <1.68 g/L for ≥11 years.^26 27 29 35^ Elevated Shock Index Paediatric Age-Adjusted (SIPA) was defined as >1.2 for 0–6 years, >1 for 7–12 years, >0.9 for >12 years.^41 42^

The direct care teams (or local research teams, which form part of the direct care team) at each site de-identified data. De-identified data were extracted into an encrypted Excel workbook (Microsoft Office Professional 2023, V.16.71, Microsoft Corporation, Redmond, WA, USA) and stored in the Queen Mary University of London (QMUL) data Safe Haven. Prism (Prism GraphPad, V.10.1.1 (270), GraphPad Software, Boston, MA, USA, www.graphpad.com) was used for basic statistical analysis. Logistic regression modelling was undertaken in Stata 19.5MP (StataCorp, College Station, TX, USA).

### Statistical methods

Basic demographics, mechanism of injury and injury data were reported as numbers (percentages) and as means (±SD) or medians (IQR), as appropriate. Missing data was not included in any analyses. Normality was assessed using the Shapiro-Wilk test and quantile-normal plots (online supplemental appendix 2). Normally distributed parameters were compared using the Student’s t-test. The Mann–Whitney U test was used for non-normally distributed variables. The χ^2^ test was used to analyse categorical variables. If any cell value fell below 5, Fisher’s exact test was used as an alternative.

The logistic regression model (online supplemental appendix 3) was adjusted for other potential variables of interest (sex, age category, mechanism, ISS). Dichotomous outcomes were analysed using multivariable logistic regression with stepwise and purposeful variable selection.^43 44^ Variables excluded during model reduction were reintroduced to assess confounding, defined as a >10% change in the primary exposure’s effect estimate.^43^ Model parsimony was prioritised using the Bayesian Information Criterion (BIC), with lower values indicating preferred models.^45 46^ Model discrimination was assessed using the area under the receiver operating characteristic curve (c statistic), and calibration was evaluated using Cox calibration plots of observed vs predicted probabilities.^43 47^ Model misspecification was assessed using Pregibon’s link test, with multivariable fractional polynomial techniques applied when indicated.^43^ Predictive marginal probabilities were used to facilitate interpretation.^47^ LOS was analysed as a secondary outcome using quantile (median) regression due to right-skewed, zero-truncated distributions.^43 48^ Coefficients represent the change in median LOS associated with each covariate.^43 48^ Heteroscedasticity was assessed using the Glejser test.^49^

### Ethics

Ethical approval was obtained from the UK Health Research Authority Research Ethics Committee (REC) under reference number 24/PR/1155.

### Patient and public involvement

Patient and Public Involvement was undertaken through the Bristol Young Persons Advisory Group (YPAG); details are available at https://generationr.org.uk/bristol/. Following consultation with the Bristol YPAG, patient information leaflets (online supplemental appendix 4) and a patient information website were made available online and for local sites to display (online supplemental appendix 5).

## Results

### Descriptive data

The study included 517 paediatric trauma patients. Most children were adolescents (236/517 (45.7%)) and male (350/517 (67.7%)), with blunt trauma accounting for the majority of injuries (489/517 (94.5%)). The median ISS was 25. The median iCa level was 1.21 mmol/L, and 102/517 (19.7%) had iHypoCa, 343/517 (66.3%) iNormoCa and 72/517 (13.9%) iHyperCa. Severe iHypoCa was observed in 13/517 (2.5%) of patients (table 1).

**Table 1:** Demographics and differential determinants

	Total (n=517)	Normocalcaemia (n=343)	Hypocalcaemia (n=102)	Hypercalcaemia (n=72)
Ionised calcium level (mmol/L), median (IQR)	1.21 (1.17–1.24)	1.21 (1.19–1.23)	1.12 (1.08–1.14)	1.36 (1.32–1.43)
Infants (0–1 years), n (%)	58 (11.2%)	17 (4.9%)	2 (1.9%), p>0.05	**39 (54.2%), p<0.001**
Toddlers (1–3 years), n (%)	47 (9.1%)	33 (9.6%)	4 (3.9%), p>0.05	10 (13.8%), p>0.05
Preschool (3–5 years), n (%)	40 (7.7%)	27 (7.9%)	10 (9.8%), p>0.05	3 (4.2%), p>0.05
School (5–12 years), n (%)	136 (26.3%)	106 (30.9%)	23 (22.6%), p>0.05	**7 (9.7%), p<0.001**
Adolescent (12–16 years), n (%)	236 (45.7%)	160 (46.7%)	**63 (61.8%), p<0.05**	**13 (18.1%), p<0.001**
Male gender, n (%)	350 (67.7%)	235 (68.5%)	69 (67.6%), p>0.05	46 (63.9%), p>0.05
ISS, median (IQR)	25 (18–32)	25 (17–30)	25 (19–33), p>0.05	**26 (25–35.5), p<0.05**
Blunt trauma, n (%)	489 (94.5%)	330 (96.2%)	96 (94.1%), p>0.05	66 (91.7%), p>0.05
Received prehospital blood product transfusion, n (%)	13 (2.5%)	3 (0.9%)	**5 (4.9%), p<0.05**	**5 (6.9%), p<0.001**
Received prehospital calcium, n (%)	7 (1.4%)	0 (0.0%)	1 (0.9%), N/A	6 (8.3%), N/A
Injury time to calcium measurement (minutes), median (IQR)	121 (86–172)	124 (93–173)	124 (91–180), p>0.05	**98 (71–143), p<0.05**
ED arrival to calcium measurement (min), median (IQR)	21 (10–53)	20 (10–60)	22 (13–55), p>0.05	21 (10–32), p>0.05

Missing data: **Arrival time**=3; **injury time**=83; **ISS**=2; **mortality**=2.

Bold text indicates a statistically significant result.

ED, Emergency Department; ISS, Injury Severity Score.

iHypoCa was observed to be significantly more prevalent in adolescents (63/102 (61.8%), p<0.05) and less common in infants and toddlers. ISS was similar to the iNormoCa group, and there were no significant differences in trauma mechanism or timing of iCa measurement compared with iNormoCa. iHyperCa was strongly associated with infancy (39/72 (54.2%), p<0.001) and was uncommon in school-aged children and adolescents. Sensitivity analyses using age-specific thresholds produced similar results, with no meaningful movement of the effect estimates towards the null (online supplemental appendix 3).^50^ Children with iHyperCa were observed to have a higher median ISS (26, p<0.05), and iCa levels were measured earlier after injury compared with children with iNormoCa (p<0.05), but there was no significant difference in the timing between ED arrival and iCa measurement.

### Outcome data

Associations between iCa disturbances and physiological derangements, treatment requirements in the first 24 hours and adverse outcomes are shown in table 2.

**Table 2:** Ionised calcium levels and physiological derangements, treatment requirements and adverse outcomes

	Total (n=517)	Normocalcaemia (n=343)	Hypocalcaemia (n=102)	Hypercalcaemia (n=72)
Acidosis, n (%)	86 (16.6%)	37 (10.8%)	13 (12.7%), p>0.05	**36 (50.0%), p<0.001**
Hyperkalaemia, n (%)	19 (3.7%)	0 (0.0%)	7 (6.8%), N/A	12 (16.7%), N/A
Hyperlactataemia, n (%)	344 (66.5%)	215 (62.7%)	71 (69.6%), p>0.05	**66 (91.7%), p<0.001**
Deranged clotting, n (%)	98 (18.9%)	53 (15.5%)	**27 (26.5%), p<0.05**	18 (25.0%), p>0.05
Hypotension, n (%)	187 (36.2%)	109 (31.8%)	**44 (43.1%), p<0.05**	**34 (47.2%), p<0.05**
Elevated SIPA, n (%)	181 (35.1%)	117 (34.1%)	27 (26.5%), p>0.05	**37 (51.4%), p<0.05**
24-hour mortality, n (%)	27 (5.2%)	7 (2.0%)	4 (3.9%), p>0.05	**16 (22.2%), p<0.001**
30-day mortality, n (%)	62 (11.9%)	22 (6.4%)	9 (8.8%), p>0.05	**31 (43.1%), p<0.001**
Poor functional outcome, n (%)	152 (29.4%)	91 (26.5%)	24 (23.5%), p>0.05	**37 (51.4%), p<0.001**
Hospital LOS (days), median (IQR)	8 (5–18)	8 (5–19)	10 (5–21), p>0.05	**6 (2–11), p<0.001**
PICU LOS (days), median (IQR)	2 (0–5)	2 (0–4)	2 (0–5), p>0.05	1 (0–6), p>0.05
Blood transfusion in the first 24 hours of admission, n (%)	96 (18.5%)	59 (17.2%)	23 (22.5%), p>0.05	14 (19.4%), p>0.05
Calcium administration in the first 24 hours of admission, n (%)	40 (7.7%)	12 (3.5%)	**15 (14.7%), p<0.001**	**13 (18.1%), p<0.001**
Vasoactive medication administration in the first 24 hours of admission, n (%)	154 (29.8%)	96 (27.9%)	26 (25.5%), p>0.05	**32 (44.4%), p<0.05**
IR or surgery in the first 24 hours of admission, n (%)	206 (39.8%)	146 (42.6%)	46 (45.1%), p>0.05	**14 (19.4%), p<0.001**

Missing data/not measured: **pH**=10; **lactate**=3; **clotting**=49; **SIPA**=38; **poor functional outcome**=27; **hospital LOS**=9; **PICU LOS**=3.

Definitions: **Acidosis**=pH<7.32; **hyperkalaemia**=potassium>5.5 mmol/L; **hyperlactataemia**=lactate≥2.0 mmol/L; **deranged clotting**=any of: raised PT≥12.6 s for <1 year, >13.4 s for 1–5 years, >14.6 s for 6–10 years, >14.1 s for ≥11 years; raised APTT≥40.7 s for <1 year, >39.2 s for 1–5 years, >38.7 s for 6–10 years, >38.4 s for ≥11 years; low fibrinogen≤1.41 g/L for <1 year, <1.64 g/L for 1–5 years, <1.71 g/L for 6–10 years, <1.68 g/L for ≥11 years; **hypotension**=systolic blood pressure of <80 mm Hg for <1 year, <85 mm Hg for 1–5 years, <90 mm Hg for 5-12 years, <100 mm Hg for >12 years; **elevated SIPA**=>1.2 for 0–6 years, >1 for 7–12 years, >0.9 for >12 years; **poor functional outcome**=Glasgow Outcome Score≤4.

Bold text indicates a statistically significant result.

IR, interventional radiology; LOS, length of stay; PICU, paediatric intensive care unit; SIPA, Shock Index Paediatric Age-Adjusted.

iHypoCa was associated with increased hypotension (44/102 (43.1%), p<0.05) compared with iNormoCa. Higher rates of deranged clotting were also observed in children with iHypoCa (27/102 (26.5%), p<0.05). Apart from the requirement for exogenous calcium replacement (15/102, (14.7%), p<0.001), there were no differences observed regarding the need for blood product transfusion, vasoactive use or need for IR/surgery in the first 24 hours. There was also no significant difference in adverse outcomes, including LOS, poor functional outcome or mortality.

iHyperCa was also associated with increased hypotension (34/72 (47.2%), p<0.05), elevated SIPA (37/72 (51.4%), p<0.05) and increased rates of acidosis and hyperlactataemia, although there was no significant difference in rates of deranged clotting. Children with iHyperCa were more likely to have received exogenous calcium replacement (13/72 (18.1%), p<0.001) compared with iNormoCa and were also observed to have a greater need for vasoactive medications (32 (44.4%), p<0.05). Poor functional outcomes (37/72 (51.4%), p<0.001), and both 24-hour mortality (16/72 (22.2%), p<0.001) and 30-day mortality (31/72 (43.1%), p<0.001) were significantly higher in children with iHyperCa compared with iNormoCa. Shorter hospital LOS and less frequent invasive management (IR/surgery) were observed in children with iHyperCa.

### Logistic regression modelling

The logistic regression modelling for iHypoCa did not demonstrate any significant associations between hypocalcaemia and any reported outcome. There was a non-significant association observed for hypotension (OR 1.50 (95% CI 0.94 to 2.40)). The logistic regression modelling for iHyperCa demonstrated a strong association with poor functional outcome, 24-hour and 30-day mortality (table 3).

**Table 3:** Logistic regression for calcium disturbances and associations with deranged physiology, treatment requirements and adverse outcomes

Outcome	Hypocalcaemia, OR (95% CI)	Hypercalcaemia, OR (95% CI)
Hypotension during emergency department stay	1.50 (0.94 to 2.40)	1.53 (0.80 to 2.93)
Elevated SIPA during emergency department stay	0.84 (0.49 to 1.42)	1.05 (0.50 to 2.22)
Need for blood transfusion in the first 24 hours	1.51 (0.83 to 2.72)	0.77 (0.33 to 1.77)
Need for vasoactive medication in the first 24 hours	0.86 (0.50 to 1.47)	1.93 (0.96 to 3.86)
Need for IR/surgery in the first 24 hours	1.07 (0.68 to 1.69)	0.46 (0.23 to 0.95)
Poor functional outcome	0.80 (0.47 to 1.37)	**2.52 (1.45 to 4.35)**
24-hour mortality	0.82 (0.26 to 2.52)	**19.33 (6.95 to 53.69)**
30-day mortality	0.91 (0.41 to 2.01)	**9.07 (4.92 to 16.72)**

Definitions: **Poor functional outcome**–Glasgow Outcome Score≤4.

Missing data: **ISS**=2; **mortality**=2; **poor functional outcome**=27.

Bold text indicates a statistically significant result.

IR, Interventional Radiology; ISS, Injury Severity Score; SIPA, Shock Index Paediatric Age-Adjusted.

Logistic regression modelling for severe iHypoCa did not observe an association with any evaluated outcome (online supplemental appendix 3). Incorporating time from injury to calcium measurement into the regression models did not result in any substantive change in the effect estimates for the three outcomes significantly associated with iHyperCa. In each case, the effect estimates shifted slightly further away from the null (table 4).

**Table 4:** Logistic regression for outcomes associated with ionised hypercalcaemia adjusted for time to ionised calcium measurement

Outcome	Main reported result for hypercalcaemia independent variable, OR (95% CI)	Result obtained when adding time from injury to calcium assessment as an additional covariate, OR (95% CI)
Poor functional outcome	2.52 (1.45 to 4.35)	2.65 (1.48 to 4.78)
24-hour mortality	19.33 (6.96 to 53.69)	19.47 (6.95 to 54.58)
30-day mortality	9.07 (4.92 to 16.72)	10.85 (5.62 to 20.93)

Missing data: **ISS**=2; **mortality**=2; **poor functional outcome**=27.

IR, interventional radiology; ISS, Injury Severity Score.

### Subgroup analysis: injury location

Abbreviated Injury Scale (AIS) data on injury location were available for 220/517 (42.6%) of patients (table 5).

**Table 5:** Ionised calcium levels and injury locations

	Total (n=220)	Normocalcaemia (n=129)	Hypocalcaemia (n=40)	Hypercalcaemia (n=51)
Isolated head injury, n (%)	41 (18.6%)	24 (18.6%)	3 (7.5%)	14 (27.5%)
Isolated chest injury, n (%)	5 (2.3%)	3 (2.3%)	2 (5.0%)	0 (0.0%)
Isolated abdominal injury, n (%)	14 (6.4%)	8 (6.2%)	6 (15%)	0 (0.0%)
Head/face most severely injured region, n (%)	110 (50.0%)	75 (58.1%)	15 (37.5%)	20 (39.2%)
Chest most severely injured region, n (%)	26 (11.8%)	13 (10.1%)	8 (20.0%)	5 (9.8%)
Abdomen most severely injured region, n (%)	33 (15.0%)	17 (13.2%)	13 (32.5%)	3 (5.9%)
Spine most severely injured region, n (%)	4 (1.8%)	3 (2.3%)	1 (2.5%)	0 (0.0%)
Pelvis most severely injured region, n (%)	2 (0.9%)	2 (1.6%)	0 (0.0%)	0 (0.0%)
Limbs most severely injured region, n (%)	9 (4.1%)	9 (7.0%)	0 (0.0%)	0 (0.0%)
Other most severely injured region, n (%)	13 (5.9%)	4 (3.1%)	1 (2.5%)	8 (15.7%)
Multiple similarly severely injured regions, n (%)	9 (4.1%)	6 (4.7%)	2 (5.0%)	1 (2.0%)

Overall, isolated head injuries were observed in 41/220 (18.6%) of patients, and head/face injuries were the most severely injured region in 110/220 (50.0%) of patients. Head injury as the most severe injury (no other regional AIS exceeded head AIS) was associated with poor functional outcome (OR 2.20 (1.01–4.81)), 24-hour mortality (OR 17.79 (4.05–61.53)) and 30-day mortality (OR 12.34 (4.82–31.58)). The abdomen (33/220 (15.0%)) and chest (26/220 (11.8%)) were the next most common severely injured regions, while other regions (spine, pelvis, limbs) were uncommon as the most severe injury sites. Pancreatic (9/517, 1.7%) and parathyroid injuries (1/517, 0.2%) were rare and occurred too infrequently to permit meaningful statistical analysis. Patients with iHypoCa had a higher proportion of abdominal (13/40 (32.5%)) and chest (8/40 (20.0%)) injuries compared with iNormoCa. Patients with iHyperCa had a higher proportion of isolated head injuries (14/51 (27.5%)). ‘Other’ non-regional injury locations (eg, burns, drowning) were also more frequent (8/51 (15.7%)).

**Table 6:** Ionised calcium levels and prehospital demographics, differential determinants and outcomes

	Total (n=328)	Normocalcaemia (n=224)	Hypocalcaemia (n=78)	Hypercalcaemia (n=26)
Ionised calcium level (mmol/L), median (IQR)	1.2 (1.16–1.23)	1.2 (1.19–1.23)	1.13 (1.09–1.14)	1.36 (1.32–1.39)
Infants (0–1 years), n (%)	14 (4.2%)	5 (2.2%), N/A	0 (0.0%), N/A	**9 (34.6%), p<0.001**
Toddlers (1–3 years), n (%)	20 (6.1%)	13 (5.8%)	2 (2.6%), p>0.05	**5 (19.2%), p<0.05**
Preschool (3–5 years), n (%)	24 (7.3%)	16 (7.1%)	7 (8.9%), p>0.05	1 (3.8%), p>0.05
School (5–12 years), n (%)	90 (27.4%)	69 (30.8%)	15 (19.2%), p>0.05	6 (23.1%), p>0.05
Adolescent (12–16 years), n (%)	180 (54.9%)	121 (54.1%)	**54 (69.2%), p<0.05**	**5 (19.2%), p<0.001**
Male gender, n (%)	219 (66.8%)	151 (67.4%)	54 (69.2%), p>0.05	14 (53.8%), p>0.05
ISS, median (IQR)	25 (19–33)	25 (18–33)	26 (21–34), p>0.05	26 (25–40), p>0.05
Blunt trauma, n (%)	309 (94.2%)	213 (95.1%)	73 (93.6%), p>0.05	23 (88.5%), p>0.05
Prehospital hypotension, n (%)	103 (31.4%)	56 (25.0%)	**28 (35.9%), p<0.05**	**19 (73.1%), p<0.001**
Prehospital elevated SIPA, n (%)	120 (36.6%)	72 (32.1%)	32 (41.0%), p>0.05	**17 (65.4%), p<0.05**
Prehospital hypothermia, n (%)	47 (14.3%)	27 (12.1%)	15 (19.2%), p>0.05	5 (19.2%), p>0.05
Received prehospital fluids, n (%)	70 (21.3%)	37 (16.5%)	21 (26.9%), p>0.05	**12 (46.2%), p<0.001**
Received prehospital blood, n (%)	10 (3.0%)	2 (0.8%)	**4 (5.1%), p<0.05**	**4 (15.4%), p<0.001**
Received prehospital calcium, n (%)	6 (1.8%)	0 (0.0%)	1 (1.2%), N/A	5 (19.2%), N/A
24-hour mortality, n (%)	7 (2.1%)	3 (1.3%)	1 (1.2%), p>0.05	**3 (11.6%), p<0.05**
30-day mortality, n (%)	32 (9.8%)	14 (6.3%)	5 (6.4%), p>0.05	**13 (50.0%), p<0.001**
Hospital LOS (days), median (IQR)	10 (6–23)	9 (6–23)	12 (6–28), p>0.05	7 (4–15), p>0.05
PICU LOS (days), median (IQR)	2 (1–6)	2 (0–5)	3 (1–6), p>0.05	**5 (2–9), p<0.05**

Definitions: **Hypotension**=systolic blood pressure of <80 mm Hg for <1 year, <85 mm Hg for 1–5 years, <90 mm Hg for 5–12 years, <100 mm Hg for >12 years; **elevated SIPA**=>1.2 for 0–6 years, >1 for 7–12 years, >0.9 for >12 years.

Missing data: **Hospital LOS**=7; **ISS**=2; **PICU LOS**=2; **SIPA**=1; **temperature**=176.

Bold text indicates a statistically significant result.

ISS, Injury Severity Score; LOS, length of stay; PICU, paediatric intensive care unit; SIPA, Shock Index Paediatric Age-Adjusted.

Among patients with prehospital data available, iHyperCa was disproportionately observed in infants (9/26 (34.6%), p<0.001), whereas iHypoCa was more common in adolescents (54/78 (69.2%), p<0.05). There were no significant differences in sex, mechanism of injury or ISS. Both iHypoCa and iHyperCa were associated with prehospital hypotension. Patients with iHypoCa and iHyperCa were also more likely to receive prehospital fluids and prehospital blood product transfusion than those with iNormoCa, with the strongest associations observed in patients with iHyperCa. Prehospital calcium administration was rare (6/328 (1.8%)) and occurred almost exclusively in patients with iHyperCa; excluding those six cases, repetition of key findings did not suggest any substantive change (online supplemental appendix 3). 24-hour mortality was observed to be higher in iHyperCa (3/26 (11.6%), p<0.05) but not in iHypoCa. Thirty-day mortality was substantially increased in patients with iHyperCa (13/26 (50.0%), p<0.001). PICU LOS was longer in iHyperCa patients (median 5 days (2–9), p<0.05), with no statistically significant differences in overall hospital LOS.

Multivariable logistic regression modelling (online supplemental appendix 3) for prehospital calcium disturbances observed no independently associated adverse outcomes for iHypoCa but demonstrated iHyperCa to be independently associated with hypotension (OR 4.43 (95% CI 1.57 to 11.99)), receipt of prehospital fluids (OR 3.32 (95% CI 1.32 to 7.82)) and receipt of prehospital blood products (OR 13.25 (95% CI 2.81 to 62.37)).

## Discussion

In this large retrospective cohort study, abnormalities in iCa on admission were prevalent among paediatric major trauma patients. iHypoCa was associated with hypotension and coagulation abnormalities on univariable analysis but was not observed to be independently associated with adverse outcomes after adjustment. In contrast, iHyperCa was associated with hypotension on univariate analysis and was strongly and independently associated with poor functional outcome and both early and late mortality.

Approximately one in five children was observed to have iHypoCa at admission, consistent with previous literature showing that iHypoCa is less common in children than in adults.^6–8 31–34^ Systematic reviews of iHypoCa in adult trauma patients have consistently demonstrated associations with shock, coagulopathy, transfusion requirements and mortality.^6–9^ In contrast to evidence among adult trauma, this study did not demonstrate an independent association between iHypoCa and treatment requirements in the first 24 hours, poor functional outcome or mortality, and only a weak, non-significant association with hypotension.^6–8^ A systematic review of current paediatric studies also did not demonstrate a significant association with mortality or PICU LOS, but did indicate that children with iHypoCa may have greater haemodynamic instability and a higher requirement for blood transfusions in the first 24 hours of admission.^31^ A small pilot study explored these associations in greater physiological detail and extended outcomes to include additional treatment requirements relevant to children, such as the need for invasive management (IR/surgery) and poor functional outcomes.^35^ In the pilot study, multivariable logistic regression showed point estimates indicating that children with iHypoCa had two- to three-fold higher odds of haemodynamic instability than those with iNormoCa and suggested associations with other adverse outcomes, such as the need for invasive management and a poor functional outcome.^35^ However, these findings were underpowered due to the small sample size (n=45) and the low incidence of iHypoCa observed in 8/45 (17.8%) patients.^35^ Previous paediatric literature contrasts with the results presented here. For the systematic review, the heterogeneity for definitions of iHypoCa ranged from <1.00 mmol/L to <1.16 mmol/L.^31–34 51^ These findings reflect the wider literature, where there remains no consensus regarding the iCa thresholds that define clinically relevant calcium disturbances, and evidence supporting age-specific reference ranges in children is limited.^17 50^ In the study reported here, <1.00 mmol/L represented severe iHypoCa and was infrequently observed; in contrast, the study by Ciaraglia *et al*, observed iHypoCa (<1.00 mmol/L) to be present in 66/142 (46.5%) of patients and this was significantly associated with hypotension and increased blood product requirement, but with no observed difference in early or in-hospital mortality.^31^ The higher incidence of severe iHypoCa reported by Ciaraglia *et al* may be explained by differences in the study population, including the exclusion of isolated head injuries and a higher proportion of penetrating trauma (38/142, 26.7%), which is likely to have been associated with a greater haemorrhagic burden.^34^ However, it is important to note that mortality in paediatric trauma is proportionally less common than adult trauma, and this provides further justification for the use of the clinically relevant physiological outcomes, treatment requirements and poor functional outcomes used in this study.^52^ Overall, these findings may suggest that iHypoCa in paediatric trauma is best interpreted contextually and as a marker of injury burden and early physiological stress rather than a direct causal mediator of adverse outcomes. It remains unclear what level of iHypoCa is clinically relevant in injured children, and future studies may benefit from further defining this.

iHyperCa was observed in approximately one in seven children and was strongly observed in infants. This was associated with adverse outcomes including poor functional outcome and both 24-hour and 30-day mortality. The apparent association between iHyperCa and reduced hospital LOS and requirement for invasive management (IR or surgery) likely reflects this early mortality rather than a protective physiological effect. No previous studies have explored iHyperCa as an exposure of interest in paediatric trauma patients.^31 32 34 51^ In the adult trauma literature, although iHyperCa has been less frequently examined than iHypoCa, emerging evidence in adults similarly demonstrates an association with adverse outcomes.^12 13 19^ A retrospective study of the TraumaRegister DGU in Germany (2015–2019) by Helsloot *et al* included 30 183 adult major trauma patients with iCa taken on ED arrival.^12^ This study observed iHyperCa in 963/30 183 (3.2%) patients and, following detailed multivariable logistic regression, demonstrated an independent association with coagulopathy, transfusion requirements and 6-hour mortality. Moreover, within this overall cohort reported by Helsloot *et al*, 1593 patients had exogenous calcium replacement.^13^ Using multivariable logistic regression, two propensity-score-matched cohorts of 1447 patients who did and did not receive exogenous calcium showed no significant difference in mortality at 6 and 24 hours.^13^ Further recent studies have demonstrated similar findings.^18 19^ There is also evidence that iHyperCa occurs early in adult trauma patients and may be present in the prehospital environment.^53 54^ The prehospital subgroup in this study similarly demonstrated early physiological derangement and association with adverse outcomes in children with iHyperCa. This is particularly relevant given that exogenous calcium replacement is often given empirically alongside blood products (with only a few UK Helicopter Emergency Medical Services currently able to measure this), even though there is substantial variation in practice and protocols.^15^ From a systems perspective, these data raise the question of whether prehospital point-of-care-testing (POCT) of iCa levels could enhance early risk stratification; however, it is unclear whether this is feasible in children, given the multiple human factors and competing demands on prehospital practitioners in the management of a severely injured child.^3^ Even among Helicopter Emergency Medical Services (HEMS), POCT measurement of iCa is rare, and there is currently no evidence on whether prehospital POCT alters management in any meaningful way.^15 54^ Overall, given the U-shaped relationship between iCa levels reported by Helsloot *et al*, with both iHypoCa and iHyperCa being associated with mortality, and taking into account the independent association with poor functional outcomes and mortality in this study, the findings may not only point towards iHyperCa being a potential biomarker of critical illness, which might be able to be included in future risk stratification models, but also suggest a need to be cautious not to overcorrect iHypoCa.^12–14^

The mechanism for iHyperCa is not completely understood.^14^ While a small proportion of children with iHyperCa received prehospital calcium supplementation, the majority did not. Sensitivity analyses excluding these patients yielded similar results. Moreover, iHyperCa was measured early after injury. These findings argue against iHyperCa being just an iatrogenic phenomenon. Recent experimental work has provided important mechanistic insights into calcium disturbances following trauma.^55^ Using a controlled porcine polytrauma model with serial physiological sampling, Helsloot *et al* demonstrated that iCa undergoes dynamic changes throughout injury and resuscitation, reflecting the combined effects of acid–base disturbances, haemorrhage, tissue hypoperfusion, metabolic derangement and endocrine responses.^55 56^ There may also be age-related physiological differences that are relevant. In this cohort, iHyperCa was more commonly seen in infants, occurred early after injury, and was associated with acidosis and hyperlactataemia. Plausible mechanisms include acidosis-related iCa shifts, haemoconcentration, severe tissue injury or age-specific changes in calcium homeostasis.^1 2 55 57^ These factors may be more pronounced in infants, owing to their smaller circulating volume, smaller total calcium stores, heightened bone turnover that amplifies inflammatory calcium release, immature parathyroid function and increased sensitivity to pH-mediated calcium shifts.^1 2 57 58^ Greater pre-analytical variability may also have been present in this age group owing to the technical challenges of obtaining blood gas samples in infants and the possibility that clinicians preferentially obtained blood gases in those perceived to be more severely unwell. Sensitivity analyses using age-specific reference ranges did not materially alter the findings. However, because the dataset used broad age categories rather than exact ages, more detailed exploration of age-related physiological variation in iCa concentrations was not possible. In particular, neonates could not be distinguished from older infants, despite the neonatal period theoretically being associated with more pronounced differences in calcium homeostasis.^58–61^ Future studies should explore these age-specific effects using more granular age data and larger sample sizes. Additionally, given the complex physiological mechanisms underlying calcium disturbances demonstrated in Helsloot *et al’s* experimental study, future studies should incorporate a broader panel of biochemical markers.^55^ Alongside iCa, this should include arterial carbon dioxide (PaCO₂), which is routinely available from blood gas analysis, as well as potassium, phosphate and magnesium, to better characterise the metabolic and physiological response to trauma.^55 56 62^ Overall, causality cannot be inferred from this data, and the observed association suggests that iHyperCa may be an area for further research and mechanistic studies in paediatric trauma.^6 63 64^

Overall, this study suggests that early measurement of iCa is valuable and may potentially indicate physiological stress. The results also go against a protocolised, one-size-fits-all approach to exogenous calcium replacement and instead may support a phenotype-based interpretation of iCa levels, cautioning against extrapolating adult trauma resuscitation paradigms to children.^65^ Further studies would benefit from exploring dosing guided by measured iCa, prospectively evaluating iCa trajectories over time rather than single admission values, and assessing whether iHyperCa adds incremental value to existing paediatric trauma scores.^66 67^

## Limitations

Strengths of this study include its large sample size, multicentre design, detailed physiological and clinically relevant outcome data and robust multivariable modelling. However, several limitations should be considered, and caution is needed to avoid inferring causality from this retrospective dataset, as residual confounders may remain despite adjustment. The multicentre design may have introduced heterogeneity and potential bias through differences in blood gas analysers, local trauma management protocols and clinician decision-making regarding when to measure iCa. For example, clinicians may have been more likely to request iCa measurements in children perceived to be more severely injured or physiologically unstable, introducing the potential for selection bias.^17^ In addition to this, although the overall sample size was large, when this was subdivided into age categories, the numbers in each category were much smaller, which may under- or overrepresent age-related changes in iCa disturbances. Similarly, the number of patients with calcium disturbances was a small proportion, and the reported effect estimates should be interpreted with caution, as the results may be susceptible to sparse-data bias. There was also insufficient data on specific injured areas (AIS scores) to include them in the regression modelling. Similarly, only a small number of patients had exogenous calcium replacement, and this was also unable to be included in the logistic regression modelling. The inclusion of patients who received prehospital blood product transfusions or exogenous calcium may also be considered a limitation. Although excluding this group might have reduced confounding, access to prehospital data was not consistently available across sites. Given that all UK HEMS services carry blood products, applying this as an exclusion criterion would have risked inadvertently including such patients due to incomplete data capture.^15^ Importantly, only a small proportion of the overall cohort were documented as receiving prehospital blood product transfusion, and blood products were administered to patients in both the iHypoCa and iHyperCa groups, reducing the likelihood of bias in one direction. Additionally, outside the prehospital setting, the timing of exogenous calcium administration was not recorded and therefore could not be confirmed as occurring before or after the index iCa measurement. However, patients receiving ED blood transfusion before calcium measurement were excluded, and empiric calcium administration before either blood transfusion or iCa measurement would not be routine clinical practice.

Since the study occurred in the interim period between TARN closing and the National Major Trauma Registry (NMTR) opening, data availability and patient identification were challenging; this meant that the total number of eligible patients was not available and led to a convenience sample rather than a consecutive sample being obtained, which could have introduced selection and data-availability bias; however, the multicentre design is likely to have reduced the effect of this.^37^ There was also a significant amount of missing data, particularly related to AIS, prehospital and additional biochemical data, which reflects the challenges with data collection. Future studies may benefit from synthesising data from UK trauma and prehospital registries which are being established.^37 68^

## Conclusion

Admission iHypoCa was prevalent but not independently associated with adverse outcomes. In contrast, iHyperCa was associated with a high-risk phenotype and was associated with poor functional outcomes and increased 24-hour and 30-day mortality. This may support consideration of iCa as a potential marker of physiological stress and highlights the need for caution with empiric calcium administration in injured children.

## Supplementary material

10.1136/bmjopen-2026-122447 online supplemental file 1

10.1136/bmjopen-2026-122447 online supplemental file 2

10.1136/bmjopen-2026-122447 online supplemental file 3

10.1136/bmjopen-2026-122447 online supplemental file 4

10.1136/bmjopen-2026-122447 online supplemental file 5

## Data Availability

Data are available upon reasonable request.
