# Supplementary material for: Admission ionised calcium disturbances and adverse outcomes in paediatric major trauma: a UK multicentre retrospective cohort study
Source: BMJ Open. 2026 Sep 18;16(9):e122447. doi: 10.1136/bmjopen-2026-122447 (PMC13599871; doi:10.1136/bmjopen-2026-122447)

Calcium levels on admission and the association with haemodynamic instability in paediatric major trauma:

Shapiro-Wilk test and quantile-normal plots

Shapiro-Wilk Test

	**iCa levels (mmol/L)**	**ISS**	**Injury time to ED arrival (minutes)**	**ED arrival to iCa (minutes)**	**Hospital LOS (days)**	**PICU LOS (days)**
W	0.7233	0.9205	0.7294	0.5068	0.5581	0.6593
P value	<0.0001	<0.0001	<0.0001	<0.0001	<0.0001	<0.0001
Passed normality test (alpha=0.05)?	No	No	No	No	No	No
Abbreviations:	***ED*** *= Emergency Department;* ***iCa*** *= Ionised Calcium;* ***ISS*** *= Injury Severity Score;* ***LOS*** *= Length of stay;* ***PICU*** *= Paediatric Intensive Care Unit*					

Quantile-normal plotting

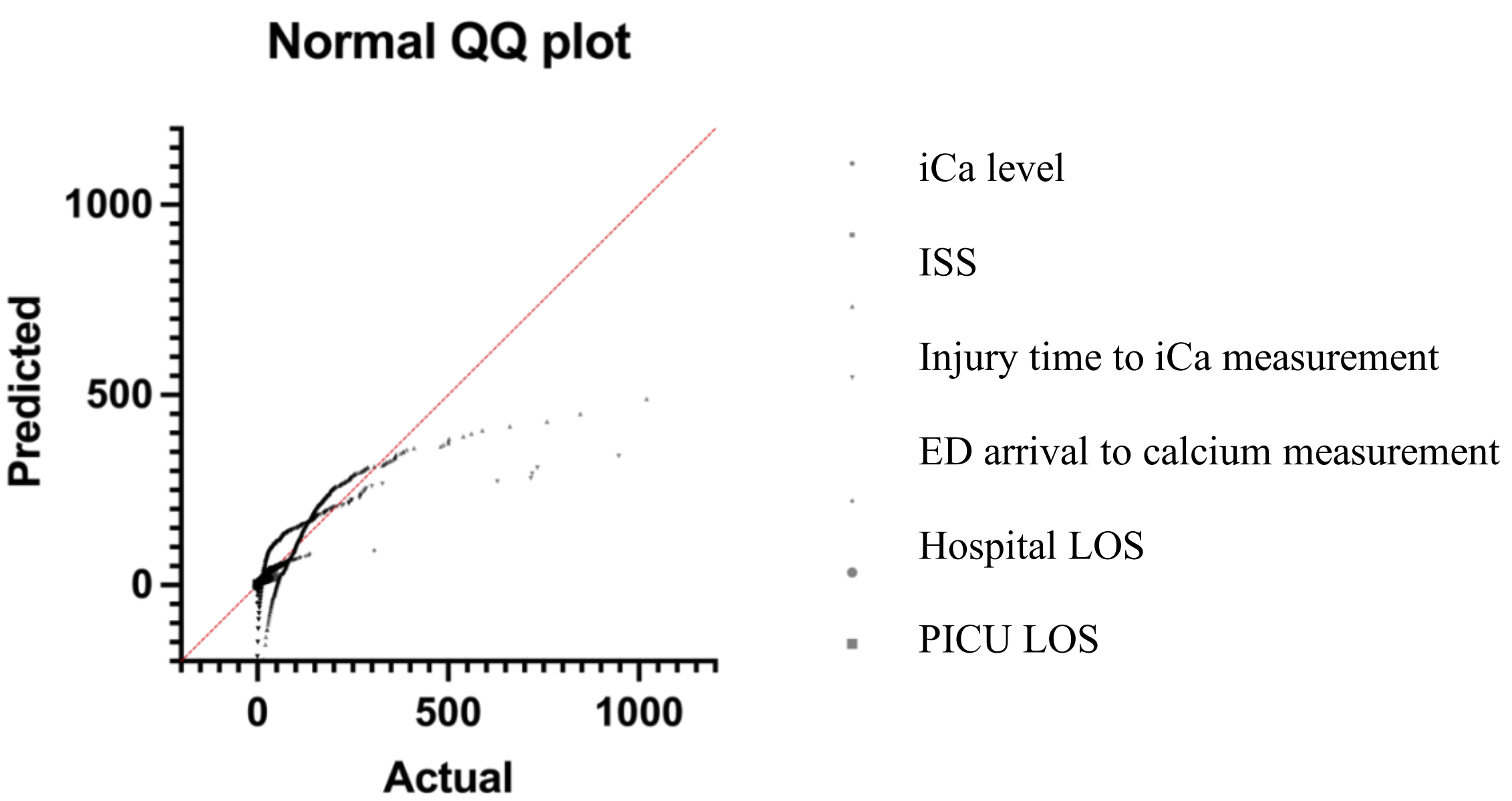

Supplement: Supplementary file 1 [file bmjopen-16-9-s002.pdf]
