# Supplementary material for: Admission ionised calcium disturbances and adverse outcomes in paediatric major trauma: a UK multicentre retrospective cohort study
Source: BMJ Open. 2026 Sep 18;16(9):e122447. doi: 10.1136/bmjopen-2026-122447 (PMC13599871; doi:10.1136/bmjopen-2026-122447)
Supplement: Supplementary file 2 [file bmjopen-16-9-s003.pdf]

STATISTICAL APPENDIX: LOGISTIC REGRESSION MODELLING

Abbreviations

AIC	Akaike's Information Criteria
AUROC	Area under receiver operator characteristic (ROC) curve; equal to *c* statistic
BIC	Bayesian Information Criteria
c	Statistic corresponding to AUROC (discrimination measure for logistic regression)
CI	Confidence interval
GLM	Generalined linear model
GOS	Glasgow Outcome Scale ("dependence" defined as any score <5)
IQR	Interquartile range
IR	Interventional radiology
LOS	Length of stay
LR+/LR-	Positive likelihood ratio/negative likelihood ratio
LRT	Likelihood ratio test (for assessment of logistic regression variables' significance)
MOI	Mechanism of injury (here, penetrating MOI is the "1" and non-penetrating is assigned "0")
NNT	Number needed to treat
OLS	Ordinary least squares (standard linear regression)
OR	Odds ratio
PPV/NPV	Positive predictive value/negative predictive value
RD	Risk difference (inverse of NNT)
RR	Risk ratio (relative risk)
SD	Standard deviation

**Some of the above abbreviations may not be used in this report.*

Definitions & modifications in data

Independent variables

There were a few changes in the data as obtained from the Excel sheet. Age is reported in bands, rather than as continuous variables. These bands were treated as ordinal in the analysis, with additional evaluation for non-linearity (although this analysis was somewhat limited by the low number of age bands).

The main independent variables of focus were:

- Hypocalcemia
- Severe hypocalcemia (evaluated only with Firth penalised regression)
- Hypercalcemia

Dependent variables & endpoints: Overall dataset

The primary patient-centred endpoint was 30-day mortality. The secondary endpoints were:

- 24-hour mortality
- Dependence (GOS 4 or less)
- Clinical findings or treatments occurring within 24 hours of admission:
    - Hypotension
    - Elevated SIPA
    - Receiving blood transfusion
    - Receiving vasopressors
    - IR or operation

Dependent variables & endpoints: Prehospital dataset

For this subset of cases, the independent variables of focus remained (hypocalcemia, severe hypocalcemia, hypercalcemia), and the prehospital-focused dependent variables consisted of *prehospital* occurrence of:

- Hypotension
- Elevated SIPA
- Hypothermia
- Fluid administration
- Blood administration
- Calcium administration

Dependent variable for supplemental analysis

As an additional secondary endpoint, the patient's LOS in the pediatric ICU was assessed.

Statistical analysis

All analysis and plotting was executed with Stata 19.5MP (StataCorp, College Station, TX, USA).

Statistical output other than that summarized in tables, is shown in `Courier font`. The Supplement to this document provides tips on reading Stata printout. (Not all information in that primer is relevant to this particular report.)

Descriptive statistics

The COWS descriptive statistics were previously executed (by OH). This report covers multivariable results.

Univariable analysis & variable selection for evaluation in modeling

Rather than take an approach of executing multiple univariable analyses to determine which variables warranted exploration in multivariable modeling, we instead took a route of predefining clinically and operationally important factors of suspected importance. Since there were relatively few such factors, and in order to preserve capability to identify important relationships (primarily confounding) that may occur in absence of a threshold *p* value, we chose to build models starting with all of the predefined variables.

The variables of interest as identified prior to analysis were:

- Sex
- Age category
- Mechanism Of Injury (MOI)
- Injury Severity Score (ISS)

Multivariable analysis

General approach for dichotomous variables: classical logistic regression

Multivariable logistic regression modeling was executed using stepwise and purposeful selection processes.[1] Variables that were discarded, were assessed for confounding by reintroduction in the final model to assess for >10% change in the magnitude of the effect estimate of the main independent variable.

Logistic regression analysis was not clustered in this analysis. Possible clustering in this type of analysis could be considered, based on EMS region or hospital. Clustering has costs (*e.g.* decrease in precision, inability to accommodate certain modeling and post-estimation commands) but may be considered in future analyses.

Due to violation of the "rare-disease" assumption (*i.e.* the 30-day death endpoint occurred in 12% of cases), logistic regression's ORs are valid as ORs *per se*[2] but they are not all presented with intent to be interpreted as approximating the RR as a measure of relative risk.[3] For endpoints that were relatively rare (*e.g.* 24-hour mortality, at 5%) the ORs are a more reasonable approximation of RR.

Once the model was reduced to only those covariates with statistical significance, post-estimation analytics were executed. Comparisons across models (including non-nested models) were made using the Bayesian Information Criterion (BIC) in order to prioritise parsimony in modelling, with classification of models with lower scores as being preferable.[5]

Logistic regression model evaluation included assessment of both discrimination and calibration. Discrimination was assessed by calculation of the ROC area (*c* statistic). Calibration was assessed using Stata's implementation of the Cox plotting approach of probability deciles' predictions *vs.* outcomes: perfect predictions lie on the 45-degree line, and an intercept at 0 is consistent with acceptable "calibration in the large" (*i.e.* lack of systemic over- or underprediction).[6,7] This approach has been recommended as being less arbitrary, and more precise, than the classically described Hosmer-Lemeshow goodness-of-fit test.[8]

Pregibon's link test was used to assess for signs of model misspecification. If misspecification were identified, multivariable fractional polynomial techniques were used to identify the best-performing (fractional) power for a particular variable.

Stata's predictive marginal probability suite was used to evaluate individual variables and facilitate model interpretation.[9]

Small-sample approach for dichotomous variables: Firth penalised logistic regression

Separation is a consequence of having relatively few events per variable (EPV). Especially for categorical variables, this renders it likely that a particular cutoff for a given covariate can completely predict the outcome. Stata reliably identifies "complete separatIon" and highlights the problem, which can then be addressed. However, quasi-separation is less readily identified by software. Quasi-separation occurs when for all but a few values in a certain range of a covariate, the outcome is perfectly predicted.

The problem with partial or quasi-separation is that Stata will drop all values of the predictor variable that completely predict success (or completely predict failure) and only keep those observations with a value of X for which there is both success and failure. Separation and quasi-separation appear a likely issue with at least some of the COWS dataset's predictors (*e.g.* severe hypocalcemia). We therefore confirmed key results using the Firth penalised likelihood methodology.[10]

First methods, which can be useful in small-sample situations (*e.g.* with low *n* of severe hypocalcemia), avoid the potentially inaccurate and inconsistent coefficients that can be calculated using non-penalised methods. We used Firth methods as a secondary check on primary findings. (Firth is not used as the primary analysis because it is more restricted in model-building – it does not accommodate likelihood ratio testing, for example – and also because Firth models' post-estimation options are limited.)

Evaluation of LOS endpoint: quantile median regression

The endpoint of LOS was evaluated as a supplemental endpoint. Since operations data such as LOS are right-skewed (and zero-truncated), we took an approach commonly used in the acute-care literature and assessed the effect of independent variables on the median LOS (as compared to the mean LOS which would be modeled using ordinary least squares regression).[11]

Median regression offers advantages of stability in calculations and robustness to highly skewed operations data distributions.[12] With the quantile regression model approach used in Stata, the coefficients are interpreted in the same fashion as they are for OLS regression, except the coefficient reports the result of an incremental change in the independent variable in the LOS *median*.[13] Stata's command suite includes a Glejser test to confirm heteroskedasticity (which finding increases likelihood that median regression in more reliable than OLS regression).[13,14]

Results: Overall cases (*n* 517)

Hypocalcemia

This section outlines results for the independent variable "hypocalcemia." Each subsection presents results for a different dependent variable (endpoint).

30-day mortality

The variable for hypocalcemia was the first variable for removal in the initial model (see below).

```
logistic mort30d hypoca male i.ageband pen iss

Logistic regression                        Number of obs =  515
                                   LR chi2(8)  = 40.45
                                   Prob > chi2  = 0.0000
Log likelihood = -167.1403                     Pseudo R2    = 0.1080

----------------------------------------------------
   mort30d | Odds ratio  Std. err.     z   P>|z|    [95% conf. interval]
----------+------------------------------------------------------------------------
    hypoca | .9110042  .3692705   -0.23  0.818    .4116144  2.016277
      male |  1.186121  .3754025    0.54  0.590    .6378628  2.205621
           |
   ageband |
    Infant |         1  (base)
   Toddler | .3767327  .1943401   -1.89  0.058    .1370677  1.035455
 Preschool | .0711747  .0755878   -2.49  0.013    .0088786  .5705644
    School | .1899487  .0845588   -3.73  0.000    .0793798  .4545301
Adolescent | .2376493  .0894005   -3.82  0.000    .113691   .4967606
           |
       pen |    2.5939  1.320318    1.87  0.061    .9564978  7.034324
       iss |   1.03857  .0115985    3.39  0.001    1.016084  1.061553
```

Removing the "male" variable instead, we still have no significance in the hypocalcemia term (see below).

```
logistic mort30d hypoca i.ageband pen iss

Logistic regression                        Number of obs =  515
                                   LR chi2(7)  = 40.16
                                   Prob > chi2  = 0.0000
Log likelihood = -167.2879                     Pseudo R2    = 0.1072

----------------------------------------------------
   mort30d | Odds ratio  Std. err.     z   P>|z|    [95% conf. interval]
---------+   ----------------------------------------------------------------------
    hypoca |   .909347  .3683233   -0.23  0.815    .4111097  2.011415
           |
   ageband |
    Infant |         1  (base)
   Toddler |   .381643  .1963119   -1.87  0.061    .1392551  1.045932
 Preschool |  .0710514  .0754481   -2.49  0.013    .0088654  .5694392
    School |  .1903723  .0846848   -3.73  0.000    .0796081  .4552504
```

```
 Adolescent |  .2411443  .0903788   -3.80  0.000    .1156789   .5026896
            |
        pen |  2.647303  1.348898    1.91  0.056    .9751812   7.186576
        iss |  1.038233  .0115381    3.38  0.001    1.015864   1.061096
```

Conclusion: there is no supporting evidence for an association between hypocalcemia and 30-day mortality.

24-hour mortality

For this analysis, the hypocalcemia variable was the second variable for discard (after PenMOI). Results of that second model – the last one including hypocalcemia – are shown below.

```
logistic mort24 hypoca male i.ageband iss
note: 3.ageband != 0 predicts failure perfectly;
      3.ageband omitted and 40 obs not used.


Logistic regression                          Number of obs =   475
                                             LR chi2(6)    =  4.79
                                             Prob > chi2   = 0.5705
Log likelihood = -98.413949                  Pseudo R2     = 0.0238

------------------------------------------------------------------------------
      mort24 | Odds ratio   Std. err.      z    P>|z|     [95% conf. interval]
-------------+----------------------------------------------------------------
      hypoca |  .8158348   .4688746    -0.35  0.723     .2644854   2.516534
        male |  1.297443   .5938134     0.57  0.569     .5290669    3.18175
             |
     ageband |
      Infant |         1  (base)
     Toddler |  .3841643   .3246797    -1.13  0.258     .0733021   2.013341
   Preschool |         1  (empty)
      School |  .3542487   .2243528    -1.64  0.101     .1023824    1.22572
  Adolescent |  .5324285    .283819    -1.18  0.237     .1872904   1.513586
             |
         iss |  1.017804   .0163366     1.10  0.272     .9862829   1.050332
```

Note there is a separation issue (ageband). Firth logit was executed (note that the coefficients are not exponentiated but the *p* values are the focus) and confirms no indication of hypocalcemia association with 24-hour mortality.

```
. firthlogit mort24 hypoca male i.ageband iss, nolog

                                             Number of obs =   515
                                             Wald chi2(7)  =  6.36
Penalized log likelihood = -90.345103        Prob > chi2   = 0.4980

------------------------------------------------------------------------------
      mort24 | Coefficient  Std. err.      z    P>|z|     [95% conf. interval]
-------------+----------------------------------------------------------------
      hypoca | -.1235536   .5459993    -0.23  0.821    -1.193693   .9465854
        male |  .2121947   .4422631     0.48  0.631    -.6546251   1.079014
             |
     ageband |
```

Infant	0 (base)					
Toddler	-.8025754	.7723271	-1.04	0.299	-2.316309	.711158
Preschool	-2.146926	1.488264	-1.44	0.149	-5.06387	.7700174
School	-1.005472	.6060019	-1.66	0.097	-2.193214	.1822698
Adolescent	-.6508152	.5154245	-1.26	0.207	-1.661029	.3593983
iss	.0184689	.0156462	1.18	0.238	-.0121971	.0491348

Conclusion: there is no supporting evidence for an association between hypocalcemia and 24-hour mortality.

Dependence

For this analysis, hypocalcemia was the third variable to be dropped (after PenMOI and male). Results of the last model containing hypocalcemia are shown below.

```
logistic depend hypoca i.ageband iss

Logistic regression                    Number of obs =  488
                                   LR chi2(6)  = 45.43
                                   Prob > chi2  = 0.0000
Log likelihood = -279.17953             Pseudo R2   = 0.0752

---------------------------------------------------------------
     depend | Odds ratio  Std. err.    z   P>|z|    [95% conf. interval]
-----------+---------------------------------------------------
     hypoca |  .7975786  .2192343  -0.82  0.411    .4653713   1.366933
            |
    ageband |
     Infant |         1  (base)
    Toddler |  .6253697   .267616  -1.10  0.273    .2703211    1.44675
  Preschool |  .6940558  .3252596  -0.78  0.436    .2770069   1.738994
     School |  .4595977  .1578332  -2.26  0.024    .2344562   .9009363
 Adolescent |  .4435146  .1432534  -2.52  0.012    .2354904   .8353002
            |
        iss |   1.05645  .0101827   5.70  0.000     1.03668   1.076597
```

Conclusion: there is no supporting evidence for an association between hypocalcemia and GOS<5 (dependence).

Hypotension

For the endpoint of hypotension, hypocalcemia was the third variable to be discarded (after male and PenMOI). Results of the last analysis including hypocalcemia are shown below.

```
. logistic hypo24 hypoca i.ageband iss

Logistic regression                    Number of obs =  515
                                   LR chi2(6)  = 39.54
                                   Prob > chi2  = 0.0000
Log likelihood = -317.08365             Pseudo R2   = 0.0587

---------------------------------------------------------------
     hypo24 | Odds ratio  Std. err.    z   P>|z|    [95% conf. interval]
-----------+---------------------------------------------------
     hypoca |  1.502743  .3584338   1.71  0.088    .9415784   2.398353
            |
    ageband |
     Infant |         1  (base)
    Toddler |  .7284319  .3021189  -0.76  0.445    .3231105   1.642203
  Preschool |  .2986838  .1514021  -2.38  0.017     .110596   .8066474
     School |  .5171685  .1735079  -1.97  0.049    .2679523   .9981751
 Adolescent |  .9240253  .2849846  -0.26  0.798    .5048465   1.691252
            |
```

iss	1.03865	.0088373	4.46	0.000	1.021473	1.056116

The above result was confirmed in Firth logistic regression, which also discarded male and PenMOI and identified a *p* value (for hypocalcemia) of .087.

Conclusion: there is at most a potential signal (given *p* = .088) of an association between hypocalcemia and hypotension within 24 hours. The current results do not provide any significant indication of such an association.

Elevated SIPA

The hypocalcemia variable was the first variable to be dropped in modeling of elevated SIPA (results below).

Logistic regression — Number of obs = 478
LR chi2(8) = 81.12
Prob > chi2 = 0.0000
Log likelihood = -276.54998 — Pseudo R2 = 0.1279

sipa24	Odds ratio	Std. err.	z	P>|z|	[95% conf. interval]	
hypoca	.8365184	.2264086	-0.66	0.510	.4921447	1.421864
male	.7886638	.1735851	-1.08	0.281	.512321	1.214064
ageband						
Infant	1 (base)					
Toddler	.6113026	.2843033	-1.06	0.290	.2456864	1.521007
Preschool	.1802103	.0907896	-3.40	0.001	.0671345	.4837415
School	.2314442	.0894154	-3.79	0.000	.1085416	.4935108
Adolescent	.1030793	.0392125	-5.97	0.000	.0489064	.2172589
pen	3.808314	1.80594	2.82	0.005	1.503435	9.646749
iss	1.031116	.0093596	3.38	0.001	1.012933	1.049624

Conclusion: there is no supporting evidence for an association between hypocalcemia and elevated SIPA.

Blood transfusion

The hypocalcemia variable was the second to be dropped (after male) in this model; results of the final model containing hypocalcemia are shown below.

Logistic regression — Number of obs = 515
LR chi2(7) = 67.66
Prob > chi2 = 0.0000
Log likelihood = -213.86826 — Pseudo R2 = 0.1366

blood	Odds ratio	Std. err.	z	P>|z|	[95% conf. interval]	

```
---------+----------------------------------------------------------------
     hypoca |  1.505743  .4533312    1.36  0.174    .8346052   2.716567
            |
    ageband |
     Infant |          1  (base)
    Toddler |  3.210433  1.660804    2.25  0.024    1.164728   8.849174
  Preschool |  2.583417   1.46564    1.67  0.094    .8497391   7.854225
     School |  1.345494  .6200261    0.64  0.520    .5452981   3.319936
 Adolescent |  1.035446  .4476047    0.08  0.936    .4437802   2.415942
            |
        pen |  6.639945  2.982591    4.21  0.000     2.75305   16.01456
        iss |  1.069338  .0109541    6.54  0.000    1.048083   1.091025
```

Conclusion: there is no supporting evidence for an association between hypocalcemia and blood transfusion.

Pressors

The hypocalcemia variable was the first to be dropped in this model; results are shown below.

```
Logistic regression                Number of obs =  515
                           LR chi2(8)  =  77.54
                           Prob > chi2  = 0.0000
Log likelihood = -274.54161            Pseudo R2   = 0.1237

---------------------------------------------------------
    pressor | Odds ratio  Std. err.     z   P>|z|    [95% conf. interval]
----------+------------------------------------------------------------
     hypoca |  .8578777  .234423   -0.56  0.575    .5021422  1.465629
       male |  1.267575  .2916767   1.03  0.303    .807434   1.989941
            |
    ageband |
     Infant |         1  (base)
    Toddler |  1.964488  .8617636   1.54  0.124    .8314808  4.641375
  Preschool |  .4523786  .2497042  -1.44  0.151    .1533399  1.334593
     School |  .7419308  .2716902  -0.82  0.415    .3619587  1.520785
 Adolescent |  .8617317  .2934463  -0.44  0.662    .442091   1.679703
            |
        pen |  .7235438  .362093   -0.65  0.518    .2713228  1.929493
        iss |  1.072997  .0106072   7.13  0.000    1.052407  1.093989
```

Conclusion: there is no supporting evidence for an association between hypocalcemia and vasopressor Rx.

IR or operation

For modeling requirement for IR or operation, the hypocalcemia variable was the second (after male) to be dropped. The results from the final model that contiained hypocalcemia are shown below.

```
Logistic regression                Number of obs =  515
                           LR chi2(7)  =  29.06
                           Prob > chi2  = 0.0001
Log likelihood = -332.06866            Pseudo R2   = 0.0419

---------------------------------------------------------
     iroror | Odds ratio  Std. err.     z   P>|z|    [95% conf. interval]
---------+------------------------------------------------------------
     hypoca |  1.072612  .2485064   0.30  0.762    .6811351  1.689086
            |
    ageband |
     Infant |         1  (base)
    Toddler |  2.351075  1.134942   1.77  0.077    .9127736  6.055776
  Preschool |  4.902073  2.385196   3.27  0.001    1.888911  12.72178
     School |  4.182199  1.67612    3.57  0.000    1.906622  9.173704
 Adolescent |  4.262823  1.633178   3.78  0.000    2.011796  9.032556
            |
        pen |  2.556215  1.109678   2.16  0.031    1.091648  5.985663
        iss |  1.018536  .008221    2.28  0.023    1.00255   1.034777
```

Conclusion: there is no supporting evidence for an association between hypocalcemia and need for IR or operation.

Severe hypocalcemia (Firth regression)

This section outlines results for the independent variable "severe hypocalcemia." Each subsection presents results for a different dependent variable (endpoint). The last five endpoints represent therapies provided within 24 hours of admission. (The same convention is followed for the remaining results presentations.)

It is important to note that the Stata software does not output Firth logit results in exponentiated (OR) form. The results are valid, but they are non-exponentiated. The *p* values are more of a key for interpretations in this section. (Results can easily be converted later as needed, but this does not seem likely necessary given negative findings.)

30-day mortality

After male, severe hypocalcemia was the second variable removed in modeling. Results of the last model including severe hypocalcemia are shown below.

```
                                  Number of obs =  515
                                  Wald chi2(7) = 38.43
Penalized log likelihood = -156.59157          Prob > chi2   = 0.0000

----------------------------------------------------------------
   mort30d | Coefficient  Std. err.     z   P>|z|    [95% conf. interval]
---------+------------------------------------------------------
 sevhypoca |  .986673  .7560784    1.30  0.192  -.4952135   2.46856
        |
   ageband |
   Infant |       0  (base)
  Toddler | -.9435703  .5013425   -1.88  0.060   -1.926184   .039043
 Preschool | -2.405247  .8990734   -2.68  0.007   -4.167399  -.6430956
   School | -1.659884  .4345159   -3.82  0.000   -2.511519  -.808248
Adolescent | -1.450212  .3587468   -4.04  0.000   -2.153343  -.747081
        |
      pen | .9480837  .4966281   1.91  0.056  -.0252895   1.921457
      iss | .0373098  .0109575   3.40  0.001   .0158335   .0587862
```

Conclusion: there is no supporting evidence for an association between severe hypocalcemia and 30-day mortality.

24-hour mortality

After removal of male, PenMOI, and ISS, severe hypocalcemia was the next variable due for removal. The results from the final model including severe hypocalcemia are shown below.

```
                                  Number of obs =  517
                                  Wald chi2(5) =  6.01
Penalized log likelihood = -99.318549          Prob > chi2   = 0.3050

----------------------------------------------------------------
```

```
     mort24 | Coefficient  Std. err.      z    P>|z|     [95% conf. interval]
---------+----------------------------------------------------------------
   sevhypoca |  1.193708  .9100996    1.31  0.190   -.5900548   2.97747
             |
     ageband |
      Infant |          0  (base)
     Toddler | -.8416868  .7721205   -1.09  0.276   -2.355015  .6716415
   Preschool | -2.439351  1.500142   -1.63  0.104   -5.379576  .5008738
      School | -1.113413  .6035466   -1.84  0.065   -2.296343  .0695165
  Adolescent | -.6702464  .4984885   -1.34  0.179   -1.647266  .3067732
```

Conclusion: there is no supporting evidence for an association between severe hypocalcemia and 24-hr mortality.

Dependence

After removal of PenMOI and male, severe hypocalcemia was the next variable due for removal. Results from the last model containing severe hypocalcemia are shown below.

```
                                        Number of obs =   488
                                        Wald chi2(6) = 40.31
Penalized log likelihood = -267.52255              Prob > chi2   = 0.0000

-----------------------------------------------------------------------
     depend | Coefficient  Std. err.      z    P>|z|     [95% conf. interval]
---------+----------------------------------------------------------------
   sevhypoca |  .3001018   .617763    0.49  0.627   -.9106914   1.510895
             |
     ageband |
      Infant |          0  (base)
     Toddler | -.4728807  .4220986   -1.12  0.263   -1.300179  .3544174
   Preschool | -.4268116  .4653733   -0.92  0.359   -1.338926   .4853032
      School | -.8053858  .3388388   -2.38  0.017   -1.469498 -.1412738
  Adolescent | -.8630809   .314855   -2.74  0.006   -1.480185 -.2459765
             |
         iss |  .0539786   .009508    5.68  0.000    .0353432   .072614
```

Conclusion: there is no supporting evidence for an association between severe hypocalcemia and GOS<5 (dependence).

Hypotension

After PenMOI and male, severe hypocalcemia was the third variable due for dropping. The results of the last model that contained severe hypocalcemia are shown below.

```
                                        Number of obs =   515
                                        Wald chi2(6) = 32.61
Penalized log likelihood = -305.6478              Prob > chi2  = 0.0000

-----------------------------------------------------------------------
     hypo24 | Coefficient  Std. err.      z    P>|z|     [95% conf. interval]
```

```
---------+----------------------------------------------------------------
  sevhypoca | -.7803326  .7214133   -1.08  0.279  -2.194277  .6336114
            |
    ageband |
     Infant |          0  (base)
    Toddler | -.2706371  .4102143   -0.66  0.509  -1.074642   .533368
  Preschool | -.9784965  .4916298   -1.99  0.047  -1.942073 -.0149197
     School | -.5792265  .3304408   -1.75  0.080  -1.226879  .0684254
 Adolescent |     .03081  .3004181   0.10  0.918  -.5579987  .6196188
            |
        iss | .0370477  .0084354   4.39  0.000   .0205147  .0535808
```

Conclusion: there is no supporting evidence for an association between severe hypocalcemia and hypotension within 24 hours of admission.

Elevated SIPA

Severe hypocalcemia was the first variable due for dropping in modeling; results are shown below.

```
                                Number of obs =   478
                                Wald chi2(8) = 64.17
Penalized log likelihood = -262.38418          Prob > chi2   = 0.0000

--------------------------------------------------------------
    sipa24 | Coefficient  Std. err.      z   P>|z|     [95% conf. interval]
---------+----------------------------------------------------
  sevhypoca | .4028376  .6034012    0.67  0.504    -.779807   1.585482
       male | -.2265588  .2179901  -1.04  0.299  -.6538115   .2006939
            |
    ageband |
     Infant |         0  (base)
    Toddler | -.4958356  .4569531   -1.09  0.278  -1.391447   .399776
  Preschool | -1.735464  .4981866   -3.48  0.000  -2.711892  -.7590362
     School |  -1.4512  .3791011   -3.83  0.000  -2.194224  -.7081753
 Adolescent | -2.269286  .3702947  -6.13  0.000  -2.995051  -1.543522
            |
        pen |  1.276174  .4639097   2.75  0.006    .3669281   2.185421
        iss |  .030173  .0089652    3.37  0.001    .0126015   .0477445
```

Conclusion: there is no supporting evidence for an association between severe hypocalcemia and elevated SIPA within 24 hours of admission.

Blood transfusion

After male, severe hypocalcemia was the second variable to be dropped. Results from the last model containing severe hypocalcemia are shown below.

```
                                Number of obs =   515
                                Wald chi2(7) = 57.85
Penalized log likelihood = -202.46335          Prob > chi2   = 0.0000

--------------------------------------------------------------
     blood | Coefficient  Std. err.      z   P>|z|     [95% conf. interval]
---------+----------------------------------------------------
  sevhypoca | .6769255  .6441158    1.05  0.293   -.5855183   1.939369
            |
    ageband |
     Infant |         0  (base)
    Toddler |  1.145002  .5076118   2.26  0.024    .1501013   2.139903
  Preschool | .9603694    .55478    1.73  0.083   -.1269793   2.047718
     School | .3156472  .4498616    0.70  0.483   -.5660653    1.19736
 Adolescent | .0992475  .4156092    0.24  0.811   -.7153315   .9138265
            |
        pen |  1.868669   .437853   4.27  0.000    1.010493   2.726845
```

iss | .0652551 .0100603 6.49 0.000 .0455373 .084973

Conclusion: there is no supporting evidence for an association between severe hypocalcemia and transfusion within 24 hours of admission.

Pressors

After PenMOI and male, the next variable due for dropping was severe hypocalcemia. The final model that included severe hypocalcemia is shown below.

```
                                    Number of obs =   515
                                    Wald chi2(6) = 60.40
Penalized log likelihood = -262.77499              Prob > chi2   = 0.0000

---------------------------------------------------------------
    pressor | Coefficient  Std. err.      z   P>|z|     [95% conf. interval]
-----------+-----------------------------------------------------
  sevhypoca | -1.135769  .8901234   -1.28  0.202   -2.880379   .6088406
            |
    ageband |
     Infant |          0  (base)
    Toddler |  .7144943  .4297298    1.66  0.096   -.1277607   1.556749
  Preschool | -.6516119  .5341706   -1.22  0.223   -1.698567   .3953432
     School | -.2667346  .3565904   -0.75  0.454    -.965639   .4321697
 Adolescent | -.1437419  .3293523   -0.44  0.663   -.7892606   .5017768
            |
        iss |  .0687622  .0097489    7.05  0.000    .0496547   .0878697
```

Conclusion: there is no supporting evidence for an association between severe hypocalcemia and pressor treatment within 24 hours of admission.

IR or operation

After male, severe hypocalcemia was the next variable due to be dropped. The results from the final model that included severe hypocalcemia are shown below.

```
                                    Number of obs =   515
                                    Wald chi2(7) = 24.13
Penalized log likelihood = -318.73965              Prob > chi2   = 0.0011

---------------------------------------------------------------
      iroror | Coefficient  Std. err.      z   P>|z|     [95% conf. interval]
-----------+-----------------------------------------------------
  sevhypoca | -.2278597  .5695594   -0.40  0.689   -1.344176   .8884561
            |
    ageband |
     Infant |          0  (base)
    Toddler |  .8381371  .4729326    1.77  0.076   -.0887939   1.765068
  Preschool |  1.585812  .4777446    3.32  0.001    .6494494   2.522174
     School |  1.400095  .3914446    3.58  0.000    .6328773   2.167312
 Adolescent |  1.423703  .3708509    3.84  0.000    .6968488   2.150558
            |
        pen |  .9220569  .4238136    2.18  0.030    .0913975   1.752716
        iss |  .0180621  .0079879    2.26  0.024    .0024062    .033718
```

Conclusion: there is no supporting evidence for an association between severe hypocalcemia and requirement for IR or operation within 24 hours of admission.

Hypercalcemia

This section outlines results for the independent variable "hypercalcemia." Each subsection presents results for a different dependent variable (endpoint). Since there were many cases with hypercalcemia, classical logistic regression was used (instead of the Firth logit procedure used for severe hypocalcemia).

30-day mortality

After removal of male (*p* = .481) and age band (all *p* .188 or higher), it was confirmed that these variables were not confounders (*i.e.* dropping the variables did not result in a substantive change in the main effect estimate for hypercalcemia). The final model for hypercalcemia and 30-day mortality is shown below.

```
Logistic regression                        Number of obs =  515
                                   LR chi2(3)  =  65.85
                                   Prob > chi2  = 0.0000
Log likelihood = -154.4423                      Pseudo R2   = 0.1757

---------------------------------------------------
   mort30d | Odds ratio   Std. err.      z   P>|z|     [95% conf. interval]
---------+-----------------------------------------------------------------
   hyperca |  9.07056  2.831467   7.06  0.000    4.919522   16.7242
       pen |  2.882486  1.518197   2.01  0.044    1.026696  8.092682
       iss |  1.036126  .0123647   2.97  0.003    1.012173  1.060646
```

The model above was confirmed in post-estimation evaluation as having acceptable calibration (Cox plot below), discrimination (*c* = .79), and specification (linktest hat^2 *p* = .901).

Figure 1. Cox predicted and observed calibration plot (hypercalcemia & 30-day mortality)

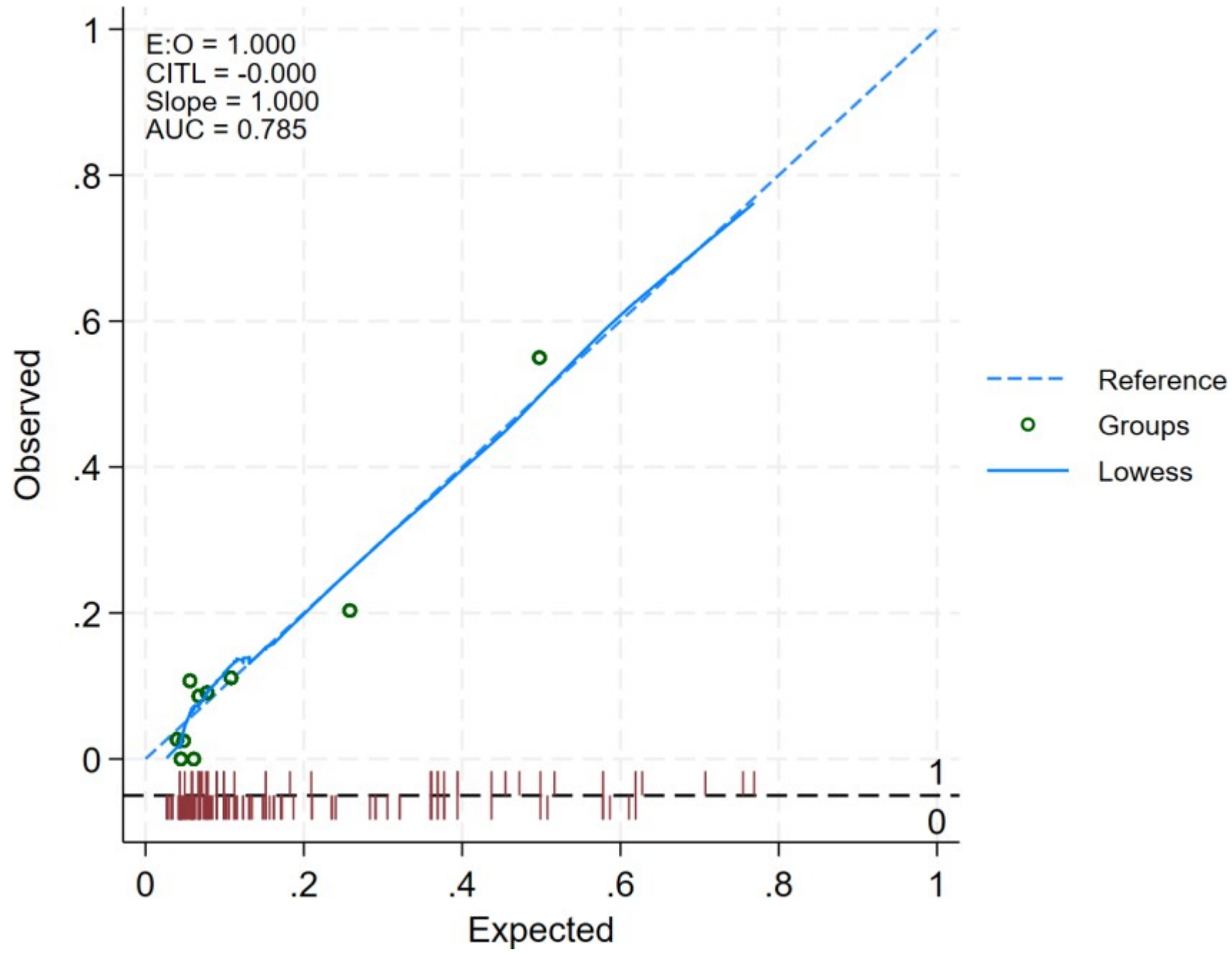

Conclusion: there is strong evidence for an association between hypercalcemia and 30-day mortality.

24-hour mortality

The evaluation of 24-hour mortality entailed an initial step of modeling that resulted in removal of the PenMOI variable (*p* = .993). The next step entailed removal of the ISS variable (as the next-highest *p*) but this step suggested confounding by ISS, with the main effect estimate (for hypercalcemia) changing by >14%. ISS was thus retained, and male was removed with no substantive effect on the main effect. This left the potential model as shown below, which was limited by the empty bin for one of the age-band groups.

```
logistic mort24 hyperca i.ageband iss, nolog

 note: 3.ageband != 0 predicts failure perfectly;
      3.ageband omitted and 40 obs not used.


Logistic regression                          Number of obs =  475
                                     LR chi2(5)  =  35.30
                                     Prob > chi2  = 0.0000
Log likelihood = -83.159715                    Pseudo R2    = 0.1751

----------------------------------------------------------------
     mort24 | Odds ratio   Std. err.      z   P>|z|     [95% conf. interval]
---------+------------------------------------------------------
    hyperca |  21.30216  11.47058   5.68  0.000    7.414413   61.2027
            |
    ageband |
     Infant |         1  (base)
    Toddler |  1.210421  1.088651   0.21  0.832    .2076668   7.055144
```

```
 Preschool |        1  (empty)
   School |  2.698352  1.979142   1.35  0.176   .6408752   11.36119
Adolescent |  4.382938  2.86389   2.26  0.024   1.217798   15.7745
          |
      iss |  1.005219  .0179452   0.29  0.771   .9706557   1.041014
```

Given the results in the above model, the analytic approach transitioned to Firth in order to better accommodate sparse data (and resulting separation) in the age band variable. The Firth modeling started with all variables and (as did classical logistic regression) discarded both PenMOI and male, while retaining ISS as a non-significant variable with suggestion of confounding. Results of the final Firth model are shown below (note that Firth results now show exponentiated forms).

```
firthlogit mort24 hyperca i.ageband iss, nolog or
                                        Number of obs =  515
                                        Wald chi2(6) = 36.86
Penalized log likelihood = -76.272639             Prob > chi2   = 0.0000

------------------------------------------------------------------------
     mort24 | Odds ratio   Std. err.      z   P>|z|     [95% conf. interval]
---------+--------------------------------------------------------------
    hyperca |  19.32925  10.07497   5.68  0.000    6.95895   53.68912
          |
    ageband |
    Infant |        1  (base)
   Toddler |  1.368237  1.132546   0.38  0.705   .2701387   6.93004
  Preschool |  .5444773  .8342949  -0.40  0.692   .0270206   10.97145
    School |  2.631674  1.854157   1.37  0.170   .661477   10.47007
 Adolescent |  4.007582  2.532967   2.20  0.028   1.161144   13.83181
          |
      iss |  1.006277  .0173699   0.36  0.717   .9728018   1.040904
```

Firth post-estimation options are limited, but the model calibration and slope are acceptable. The CITL indicates that the model slightly overpredicts risk; the slope just above 1.000 indicates the model is silghtly underfitted. The Cox plot is shown in the following figure.

Figure 2. Firth model: Cox predicted and observed calibration plot (hypercalcemia & 24-hour mortality)

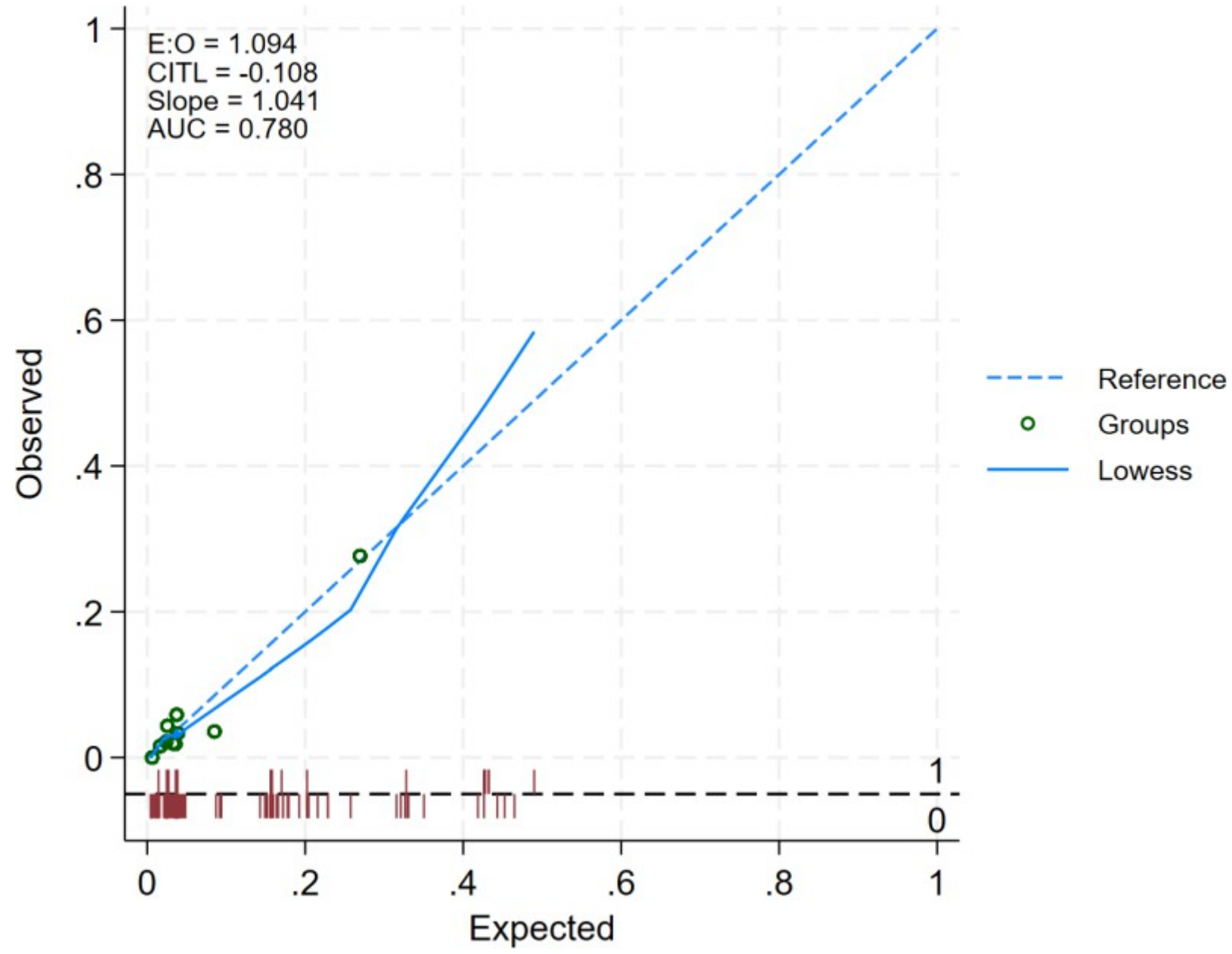

Because the Firth result essentially confirmed the finding of the classical logistic regression result, that classical regression was re-run. Post-estimation confirmed adequate specification (linktest p = .310), discrimination (c = .76), and calibration as shown in the figure below.

Figure 3. Cox predicted and observed calibration plot (hypercalcemia & 24-hour mortality)

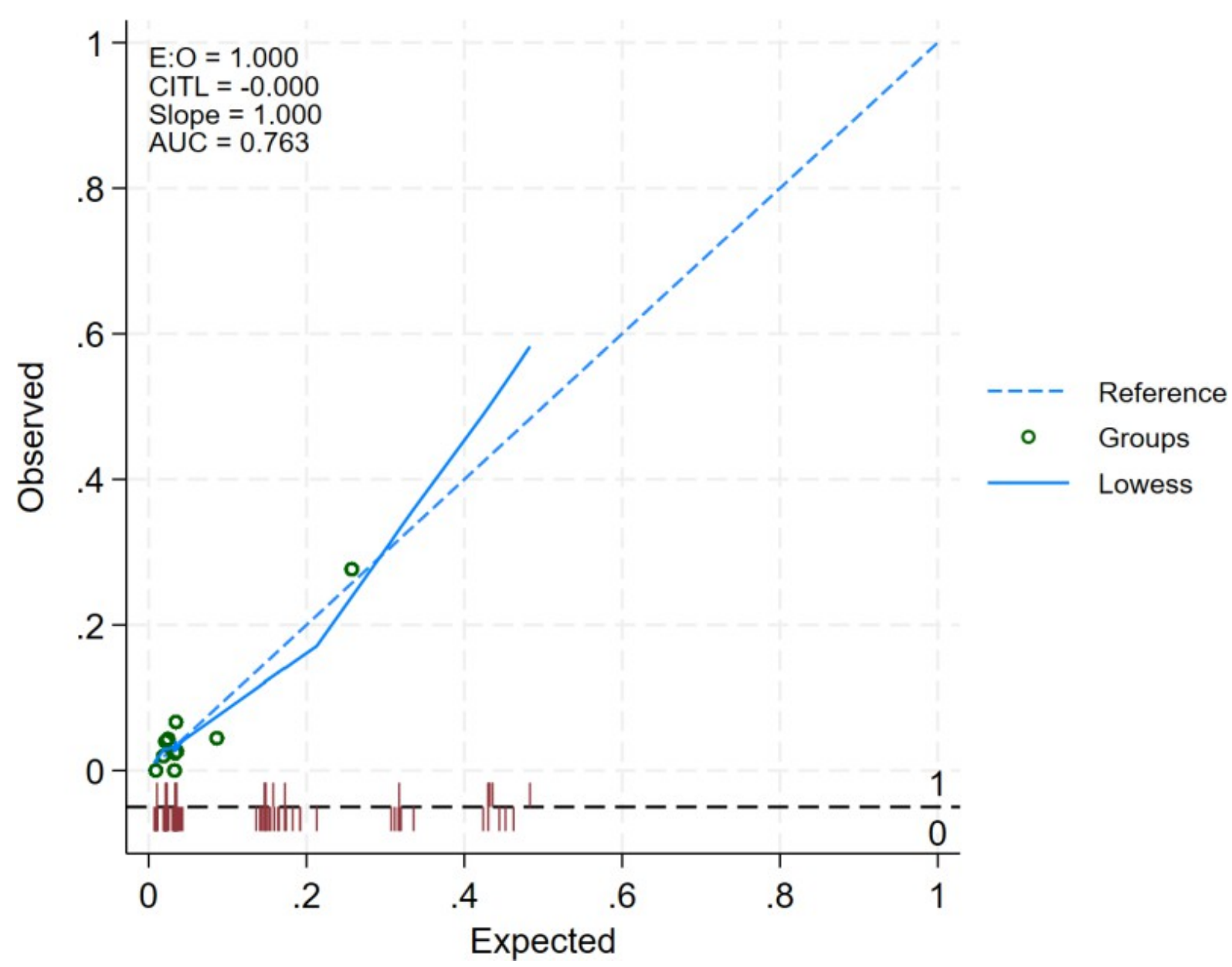

Conclusion: there is strong evidence for an association between hypercalcemia and 24-hour mortality.

Dependence

In modeling for dependence, all variables but hypercalcemia and ISS dropped. However, the Pregibon's test strongly suggested misspecification (p = .003). Multivariable fractional polynomial analysis was used to elucidate the preferred form for ISS (a power of -2 was ultimately identified based on optimal deviance), and the resulting model (shown below) was no longer misspecified (linktest p = .924) and had acceptable discrimination (.73) and calibration (as shown in figure below.

```
Logistic regression                      Number of obs =  488
                                    LR chi2(2)  =  70.75
                                    Prob > chi2  = 0.0000
Log likelihood = -266.52222                   Pseudo R2   = 0.1172

-----------------------------------------------------------------
     depend | Odds ratio  Std. err.      z   P>|z|     [95% conf. interval]
---------+-------------------------------------------------------
    hyperca |  2.515649  .7041761   3.30  0.001    1.453393  4.354285
    Iiss_1 |  .0007148  .0008001  -6.47  0.000    .0000797   .0064107
```

Figure 4. Cox predicted and observed calibration plot (hypercalcemia & dependence)

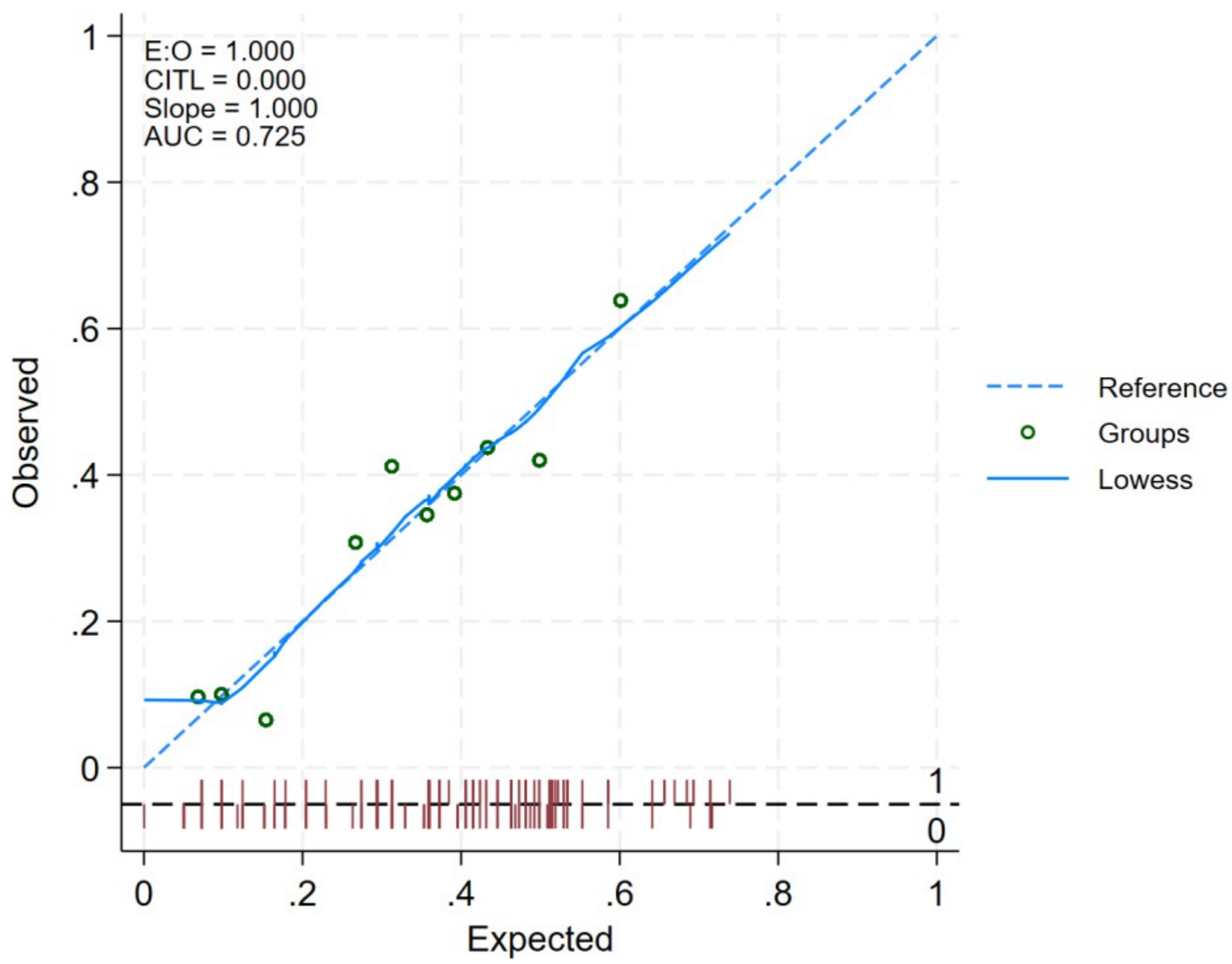

Conclusion: there is strong evidence for an association between hypercalcemia and dependence (GOS<5).

Hypotension

The variables male and PenMOI were dropped first in this modeling, with the hypercalcemia variable the third variable dropped in the modeling procedure. The results from the final model that contained hypercalcemia are tabulated in the output below.

```
Logistic regression                         Number of obs =  515
                                    LR chi2(6)  =  38.29
                                    Prob > chi2  = 0.0000
Log likelihood = -317.70766                      Pseudo R2    = 0.0568

-----------------------------------------------------
   hypo24 | Odds ratio  Std. err.     z   P>|z|    [95% conf. interval]
---------+-------------------------------------------------------------
  hyperca | 1.529357  .5065535  1.28  0.200   .7990497   2.927142
          |
  ageband |
   Infant |        1  (base)
  Toddler |  .914692  .4090199  -0.20  0.842   .3807551  2.197374
Preschool |  .4261428  .2294138  -1.58  0.113   .148358   1.22405
   School | .7156141  .2818918  -0.85  0.396   .3306565  1.548748
Adolescent | 1.329527  .4919214   0.77  0.441    .643806   2.745614
          |
      iss | 1.037484  .0088838   4.30  0.000   1.020217   1.055043
```

Conclusion: there is no evidence for association between hypercalcemia and hypotension within 24 hours of admission.

Elevated SIPA

In this analysis hypercalcemia was the first variable to be discarded (results shown below).

```
Logistic regression                         Number of obs =  478
                                    LR chi2(8)  =  80.69
                                    Prob > chi2  = 0.0000
Log likelihood = -276.7612                      Pseudo R2    = 0.1272

-----------------------------------------------------
   sipa24 | Odds ratio  Std. err.     z   P>|z|    [95% conf. interval]
---------+-------------------------------------------------------------
  hyperca | 1.051546  .4002646   0.13  0.895   .4986819  2.217344
     male | .7896257  .173721  -1.07  0.283   .5130425  1.215316
          |
  ageband |
   Infant |        1  (base)
  Toddler | .618438  .3056033  -0.97  0.331   .2347858  1.628998
Preschool | .1788451  .0967433  -3.18  0.001   .0619489  .5163219
   School | .2333649  .1030768  -3.29  0.001   .0981887  .5546377
Adolescent | .1022032  .0448993  -5.19  0.000   .0432035  .241774
          |
      pen |  3.72608  1.761651   2.78  0.005   1.475072  9.412201
      iss | 1.031013  .0094095   3.35  0.001   1.012735  1.049621
```

Conclusion: there is no evidence for association between hypercalcemia and elevated SIPA within 24 hours of admission.

Blood transfusion

In the blood transfusion (within 24 hours) analysis, male was the first variable to be dropped and the second variable out was hypercalcemia. The results of the final model that contained the variable hypercalcemia are shown in the Stata output below.

```
Logistic regression                    Number of obs =    515

                                LR chi2(7)  = 66.26
                                Prob > chi2  = 0.0000
Log likelihood = -214.5687               Pseudo R2    = 0.1338

--------------------------------------------------
     blood | Odds ratio   Std. err.      z   P>|z|     [95% conf. interval]
---------+-----------------------------------------------------------------
   hyperca |  .7672622  .3269249   -0.62  0.534    .3328534   1.768621
           |
   ageband |
   Infant  |          1  (base)
  Toddler  |  2.908845  1.611617    1.93  0.054    .9820208   8.616293
 Preschool |  2.467035  1.507873    1.48  0.140    .7445838  8.174045
   School  |  1.222942  .6424606    0.38  0.702    .4367523   3.424335
Adolescent |  .9811938  .4875389   -0.04  0.970    .3705159    2.59838
           |
       pen |  6.854755  3.061352    4.31  0.000    2.856558   16.44905
       iss |  1.069347   .01094     6.55  0.000    1.048119   1.091006
```

Conclusion: there is no evidence for association between hypercalcemia and transfusion within 24 hours of admission.

Pressors

After discarding PenMOI and male, the third variable to be discarded from the analysis was hypercalcemia. However, in this case the *p* value – which was non-significant – was relatively low (p = .065). Results of the final model that included hypercalcemia are shown below.

```
Logistic regression                    Number of obs =   515
                                LR chi2(6)   =  79.13
                                Prob > chi2   = 0.0000
Log likelihood = -273.74644              Pseudo R2    = 0.1263

--------------------------------------------------
   pressor | Odds ratio   Std. err.      z   P>|z|     [95% conf. interval]
---------+-----------------------------------------------------------------
```

hyperca	1.925635	.6832243	1.85	0.065	.9606468	3.859973

ageband						
Infant	1 (base)					
Toddler	2.855117	1.371199	2.18	0.029	1.113847	7.318502
Preschool	.6664268	.3921665	-0.69	0.490	.2103067	2.111795
School	1.143404	.4930791	0.31	0.756	.4910539	2.66238
Adolescent	1.301219	.532569	0.64	0.520	.5834001	2.902245
iss	1.071746	.0106081	7.00	0.000	1.051155	1.092741

Conclusion: there is no significant finding for association between hypercalcemia and pressors within 24 hours of admission. However, and with the *caveat* that some journals like this wording and others don't, the association approached (while not reaching) statistical significance.

IR or operation

Modeling for this endpoint commenced with discarding male. All other variables were significant. Furthermore, the link test did not indicate any misspecification (p = .563). Thus the final logistic regression model for this (final) endpoint of IR or operative intervention, included hypercalcemia, age band, penetrating MOI, and ISS. The results are shown below. It is noted that post-estimation identified only marginal discrimination (c = .65) performance; calibration was acceptable as shown in the figure below.

```
Logistic regression                      Number of obs =  515
                                     LR chi2(7)  =  33.59
                                     Prob > chi2  = 0.0000
Log likelihood = -329.80606                 Pseudo R2   = 0.0485
```

iroror	Odds ratio	Std. err.	z	P>|z|	[95% conf.	interval]
hyperca	.4630692	.1703802	-2.09	0.036	.2251425	.9524328
ageband						
Infant	1 (base)					
Toddler	1.68865	.8590225	1.03	0.303	.6230607	4.576664
Preschool	3.315757	1.71959	2.31	0.021	1.199885	9.162747
School	2.745977	1.220546	2.27	0.023	1.14908	6.562106
Adolescent	2.808228	1.19923	2.42	0.016	1.216001	6.48531
pen	2.683098	1.173552	2.26	0.024	1.138497	6.323259
iss	1.020535	.0083434	2.49	0.013	1.004313	1.037019

Figure 5. Cox predicted and observed calibration plot (hypercalcemia & IR/operation)

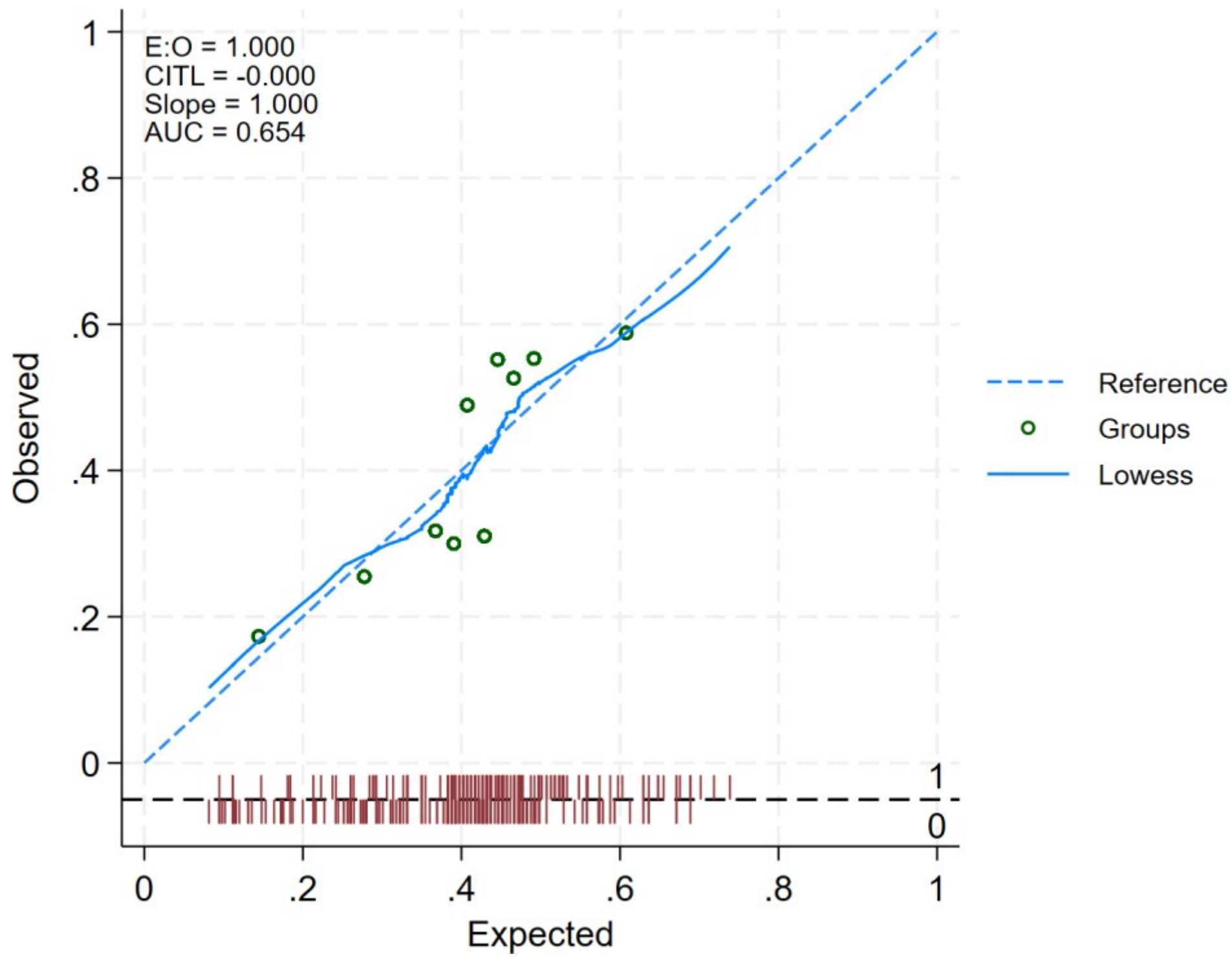

Conclusion: there is a suggestion of (protective) association between hypercalcemia and IR/operation, but the model performance was borderline and these findings should be considered preliminary.

Results: Prehospital cases (*n* 328)

Hypocalcemia

This section outlines results for the independent variable "hypocalcemia." Each subsection presents results for a different dependent variable (endpoint).

Hypotension

After removing male and PenMOI, the next variable due for removal was hypocalcemia. The final model that retained hypocalcemia is shown below.

```
Logistic regression                    Number of obs =  328
                                       LR chi2(6)   =  32.65
                                       Prob > chi2  = 0.0000
Log likelihood = -187.78313            Pseudo R2    = 0.0800

------------------------------------------------------------------------
   emslowbp | Odds ratio  Std. err.     z   P>|z|    [95% conf. interval]
---------+--------------------------------------------------------------
     hypoca |  1.60933  .4649232    1.65  0.100    .9135649   2.834985
            |
    ageband |
     Infant |        1  (base)
```

Toddler | .2651218 .2124865 -1.66 0.098 .0551106 1.275427
Preschool | .117948 .0954074 -2.64 0.008 .0241634 .5757349
School | .0925222 .0650865 -3.38 0.001 .0233052 .3673161
Adolescent | .1050326 .071959 -3.29 0.001 .0274257 .4022457
|
iss | 1.03605 .0108421 3.38 0.001 1.015016 1.057519

Conclusion: there is no significant evidence for association between hypocalcemia and prehospital hypotension.

Elevated SIPA

After removing the variable male, the hypocalcemia variable was next due for removal. The output below shows the final model that included hypocalcemia.

```
Logistic regression                    Number of obs =  327
                                   LR chi2(7)  =  43.42
                                   Prob > chi2  = 0.0000
Log likelihood = -194.29965                Pseudo R2   = 0.1005

---------------------------------------------------
    emshisi | Odds ratio  Std. err.     z   P>|z|    [95% conf. interval]
---------+-----------------------------------------------------------
     hypoca | 1.646594  .4672301   1.76  0.079   .9441766  2.871571
           |
    ageband |
    Infant |       1  (base)
   Toddler | .3666302  .334362  -1.10  0.271   .0613682  2.190347
  Preschool | .1767998  .1570662  -1.95  0.051   .0309947   1.0085
    School | .0768397  .0625552  -3.15  0.002   .015582  .3789205
 Adolescent | .0831254  .0664529  -3.11  0.002   .0173483  .3982995
           |
       pen | 4.580759  2.488054   2.80  0.005   1.57981  13.28221
       iss | 1.029852  .010678   2.84  0.005   1.009135  1.050995
```

Conclusion: there is no significant evidence for association between hypocalcemia and prehospital elevated SIPA.

Hypothermia

For hypothermia modeling, all variables were discarded until there remained the hypocalcemia variable and ISS. The results of this model are shown below.

```
Logistic regression                    Number of obs =  152
                                   LR chi2(2)  =  10.84
                                   Prob > chi2  = 0.0044
Log likelihood = -88.587802                Pseudo R2   = 0.0576

---------------------------------------------------
    emslowt | Odds ratio  Std. err.     z   P>|z|    [95% conf. interval]
---------+-----------------------------------------------------------
     hypoca | 1.523843  .6151812   1.04  0.297   .6907279  3.361813
```

iss | 1.047339 .0163243 2.97 0.003 1.015827 1.079828

Conclusion: there is no significant evidence for association between hypocalcemia and hypothermia.

Fluid administration

After discarding male and PenMOI the next variable for dropping was hypocalcemia. The output below shows the final model that retained hypocalcemia.

```
Logistic regression                     Number of obs =  328
                                LR chi2(6)  = 41.05
                                Prob > chi2  = 0.0000
Log likelihood = -149.52683                  Pseudo R2   = 0.1207

--------------------------------------------------
    fluids | Odds ratio  Std. err.     z   P>|z|     [95% conf. interval]
---------+----------------------------------------------------------------
    hypoca |  1.81683  .6024917    1.80  0.072    .9485085   3.480063
           |
   ageband |
   Infant |        1  (base)
  Toddler | .4019061  .3211931   -1.14  0.254    .0839201 1.924789
 Preschool | .4154326  .3272637   -1.12  0.265    .088706 1.945576
   School | .2901681  .1850003   -1.94  0.052   .0831678  1.012381
Adolescent |  .343884  .2095889  -1.75  0.080    .1041419   1.13553
           |
      iss |  1.06908  .0129478    5.52  0.000    1.044002  1.094761
```

Conclusion: there is no significant evidence for association between hypocalcemia and fluid administration, although the *p* value of .072 approached – without reaching – the predefined threshold defining significance.

Blood transfusion

The analysis for blood transfusion was complicated by empty bins in the age bands. The classical logistic regression did not seem indicative of any statistically significant finding. The results from the classical regression are shown below. These results were confirmed using Firth penalized regression (which was better suited to execute calculations in the setting of empty age bins). The Firth results were not substantively different from those obtained for the variable hypocalcemia, in the classical logistic regression. (The Firth results removed age band early, and the final model that included hypocalcemia included only hypocalcemia with p = .161 and ISS as the only significant variable with p = .022).

```
Logistic regression                     Number of obs =  290
                                LR chi2(4)  =  8.64
                                Prob > chi2  = 0.0709
Log likelihood = -39.18056                  Pseudo R2   = 0.0993

--------------------------------------------------
```

```
     blood | Odds ratio   Std. err.      z   P>|z|     [95% conf. interval]
---------+----------------------------------------------------------------
    hypoca |  2.487729   1.763159     1.29   0.198     .6201841    9.978966
           |
   ageband |
    Infant |          1  (empty)
   Toddler |  3.050337   2.732681     1.24   0.213     .5269736    17.65659
 Preschool |          1  (empty)
    School |  .3233753   .3521594    -1.04   0.300     .0382594    2.733223
Adolescent |          1  (omitted)
           |
       iss |  1.043558   .0224523     1.98   0.048     1.000467    1.088504
```

Conclusion: there is no significant evidence for association between hypocalcemia and prehospital blood administration.

Calcium administration

The analysis for calcium administration was complicated by empty bins in the age bands. The classical logistic regression did not seem indicative of any statistically significant finding. The results from the classical regression are shown below (hypocalcemia was the first variable to be removed). These results were confirmed using Firth penalized regression (which was better suited to execute calculations in the setting of empty age bins). The Firth results were not substantively different from those obtained for the variable hypocalcemia, in the classical logistic regression. (The Firth results also showed that hypocalcemia was the first variable removed with $p = .809$.)

```
Logistic regression                         Number of obs =   274
                                            LR chi2(5)    =  16.51
                                            Prob > chi2   = 0.0055
Log likelihood = -20.605825                 Pseudo R2     = 0.2861

-------------------------------------------------------------------------
        ca | Odds ratio   Std. err.      z   P>|z|     [95% conf. interval]
---------+----------------------------------------------------------------
    hypoca |  1.089084   1.306674     0.07   0.943     .1037043    11.43737
      male |  .0504308   .0661766    -2.28   0.023     .0038524    .6601823
           |
   ageband |
    Infant |          1  (empty)
   Toddler |  12.45005   14.22134     2.21   0.027     1.326992    116.8084
 Preschool |          1  (empty)
    School |  .6595026   .7999539    -0.34   0.731     .0611982    7.107135
Adolescent |          1  (omitted)
           |
       pen |          1  (omitted)
       iss |  1.071605   .0309887     2.39   0.017     1.012558    1.134096
```

Severe hypocalcemia (Firth regression)

Based on the lack of any findings in the larger group analysis (based on severe hypocalcemia) and based on the even smaller *n* for the prehospitla group, these analyses have been held for now.

Hypercalcemia

This section outlines results for the independent variable “hypercalcemia.” Each subsection presents results for a different dependent variable (endpoint).

Hypotension

Hypotension modeling indicated an association between hypercalcemia and prehospital low BP. The model (shown below) had acceptable specification (linktest p = .445), and calibration (shown in figure below) but discrimination was marginal (c = .66).

```
Logistic regression                         Number of obs =  328
                                    LR chi2(6)  =  38.34
                                    Prob > chi2  = 0.0000
Log likelihood = -184.9384                       Pseudo R2    = 0.0939

--------------------------------------------------------------------
   emslowbp | Odds ratio  Std. err.      z   P>|z|     [95% conf. interval]
---------+----------------------------------------------------------
    hyperca |  4.344522  2.250765   2.84  0.005    1.573839  11.99289
            |
    ageband |
     Infant |         1  (base)
    Toddler |    .45785   .390427  -0.92  0.360    .0860735  2.435436
  Preschool |   .289293  .2477409  -1.45  0.148    .0540004   1.54981
     School |  .2014946  .1528016  -2.11  0.035    .0455788  .8907677
 Adolescent |  .2628734  .1959459  -1.79  0.073    .0609903  1.133007
            |
        iss |  1.035024  .0110691   3.22  0.001    1.013555  1.056948
```

Figure 6. Cox predicted and observed calibration plot (hypercalcemia & EMS hypotension)

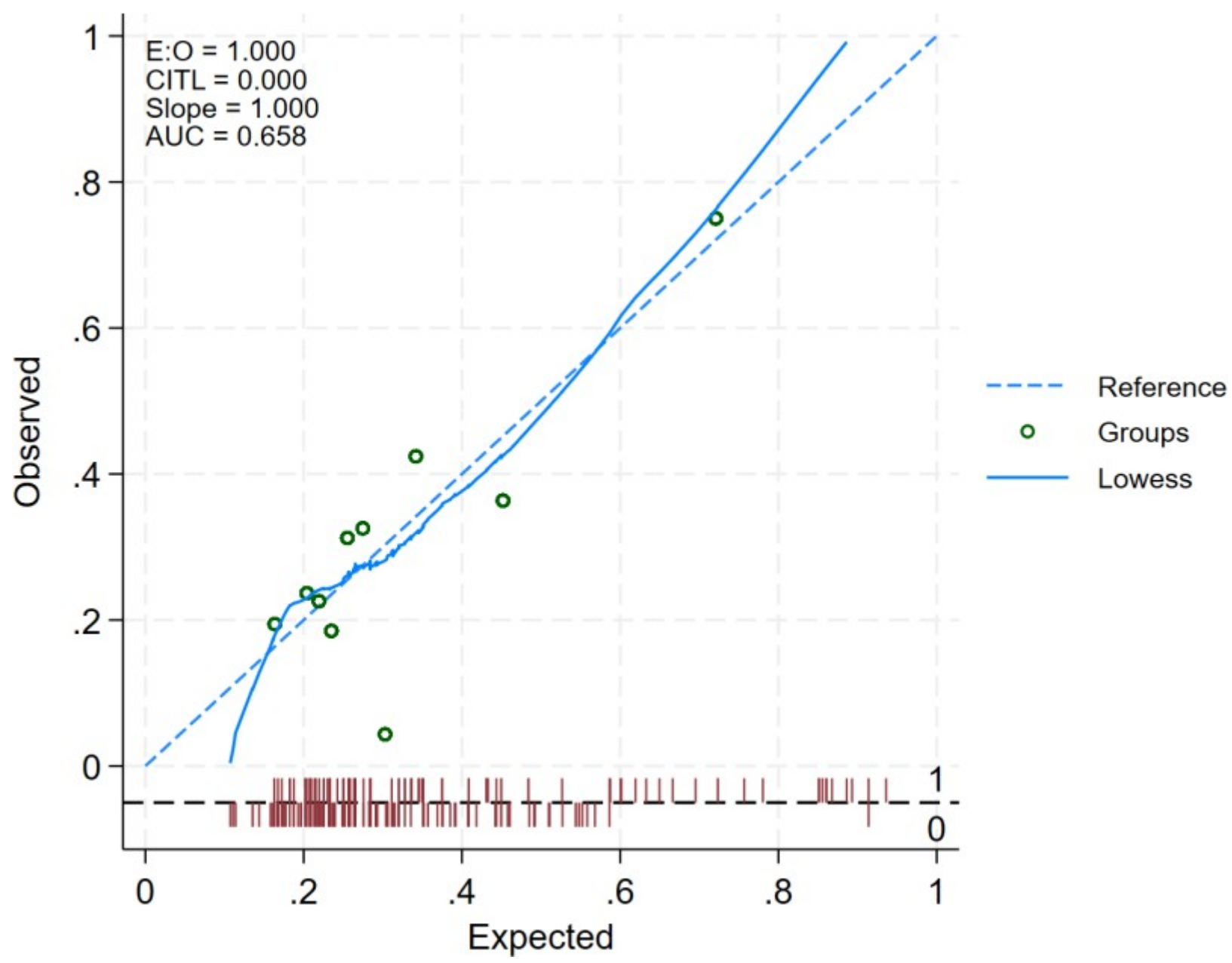

Conclusion: there is evidence for association between hypercalcemia and prehospital hypotension but the model performance was marginal and results should be considered preliminary.

Elevated SIPA

The hypercalcemia variable was the first to be dropped in this analysis. Results are shown below.

Logistic regression Number of obs = 327
LR chi2(8) = 42.12
Prob > chi2 = 0.0000
Log likelihood = -194.94804 Pseudo R2 = 0.0975

emshisi	Odds ratio	Std. err.	z	P>|z|	[95% conf. interval]	
hyperca	1.507299	.787364	0.79	0.432	.5414509	4.196038
male	.7643529	.1996996	-1.03	0.304	.4580403	1.275511
ageband						
1	1 (base)					
2	.4563261	.426712	-0.84	0.401	.0729985	2.852572
3	.2572814	.2381223	-1.47	0.142	.0419366	1.578422
4	.1035936	.0886008	-2.65	0.008	.0193786	.5537864
5	.1231471	.1040203	-2.48	0.013	.0235195	.6447923
pen	4.804665	2.612891	2.89	0.004	1.654853	13.94976
iss	1.028786	.0106517	2.74	0.006	1.00812	1.049876

Conclusion: there is no significant evidence for association between hypercalcemia and prehospital elevation in SIPA.

Hypothermia

All variables were dropped in modeling, until there were only hypercalcemia and ISS. These results are shown below.

```
Logistic regression                     Number of obs =   152
                                LR chi2(2)  =  11.23
                                Prob > chi2  = 0.0036
Log likelihood = -88.389908                   Pseudo R2    = 0.0598

---------------------------------------------------
   emslowt | Odds ratio   Std. err.      z   P>|z|     [95% conf. interval]
---------+-----------------------------------------
   hyperca |  2.292432  1.552788   1.22  0.221    .6077591   8.646917
       iss |  1.047478  .0163001   2.98  0.003    1.016012   1.079917
```

Conclusion: there is no significant evidence for association between hypercalcemia and prehospital hypothermia.

Fluid administration

In modeling, all variables were removed with the exception of hypercalcemia and ISS. Both of these were significant, and the post-estimation evaluation suggested acceptable model specification (linktest *p* = .372), discrimination (*c* = .73), and calibration (as shown in figure below). The model is shown below, with the calibration on next page.

```
Logistic regression                     Number of obs =   328
                                LR chi2(2)  =  40.91
                                Prob > chi2  = 0.0000
Log likelihood = -149.59768                   Pseudo R2    = 0.1203

---------------------------------------------------
    fluids | Odds ratio   Std. err.      z   P>|z|     [95% conf. interval]
---------+-----------------------------------------
   hyperca |  3.212701  1.458593   2.57  0.010    1.319525   7.822091
       iss |  1.066962  .012946   5.34  0.000    1.041888   1.09264
```

Figure 7. Cox predicted and observed calibration plot (hypercalcemia & EMS fluids)

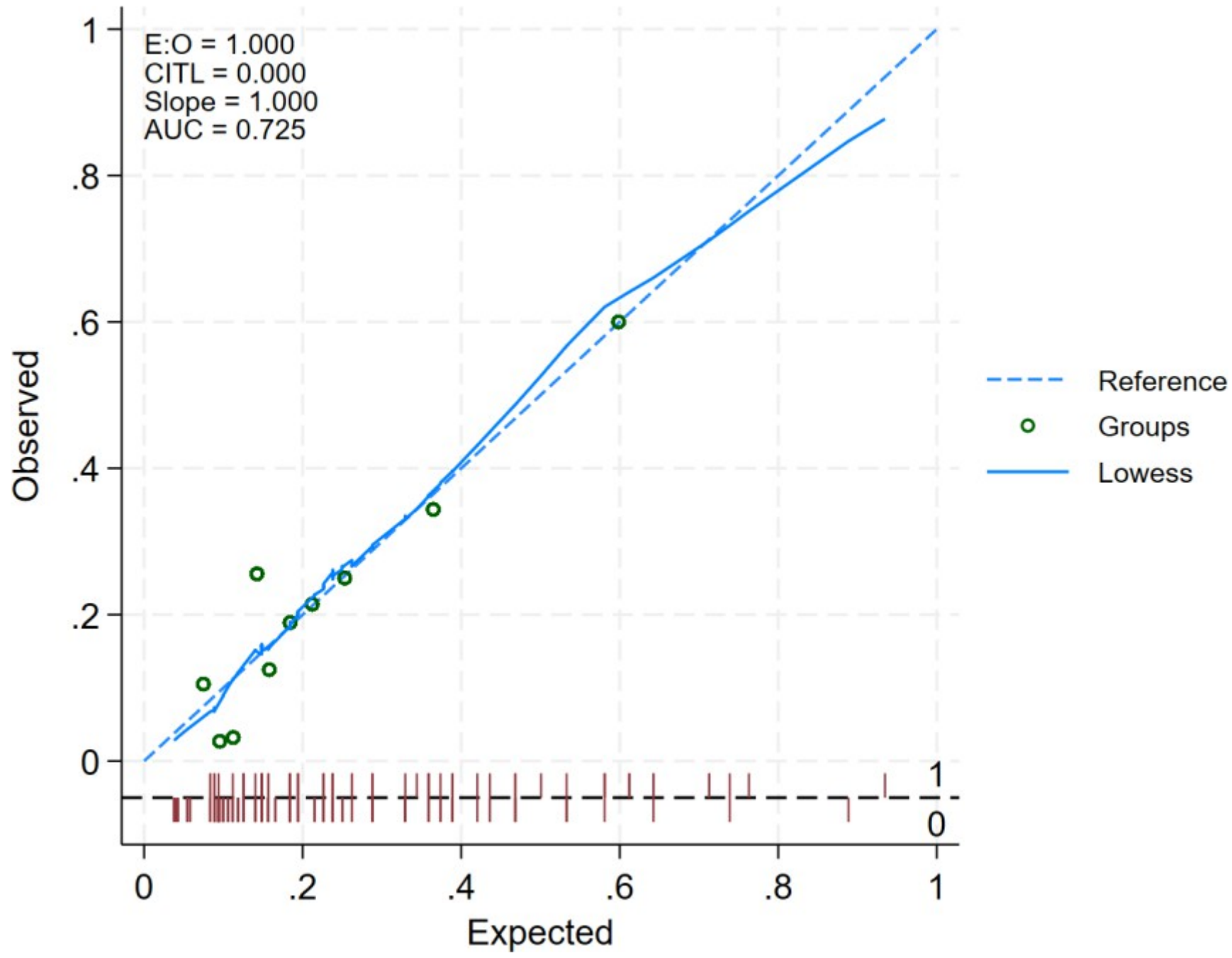

Conclusion: there is evidence for association between hypercalcemia and prehospital fluid administration.

Blood transfusion

Analysis for blood transfusion was limited by empty bins in the age band variable. The classical logistic regression modeling ended with retention of the age bands (due to empty bins and perfect separation) and ISS (due to confounding when removed from model with over 20% change in main effect on ISS removal). This model is shown below.

Logistic regression Number of obs = 290
LR chi2(4) = 16.00
Prob > chi2 = 0.0030
Log likelihood = -35.497753 Pseudo R2 = 0.1839

blood	Odds ratio	Std. err.	z	P>|z|	[95% conf. interval]	
hyperca	15.45462	13.21282	3.20	0.001	2.892874	82.56333
ageband						
Infant	1 (empty)					
Toddler	.7955824	.84216	-0.22	0.829	.0999194	6.33462
Preschool	1 (empty)					
School	.2010037	.2272296	-1.42	0.156	.0219251	1.842748
Adolescent	1 (omitted)					
iss	1.026095	.0227879	1.16	0.246	.9823895	1.071745

Due to the separation problem, the suggested association between hypercalcemia and blood transfusion was reassessed using Firth penalized regression. The Firth model results are shown below. Firth modeling suggested confounding of the

main effect (hypercalcemia OR) by both age band and ISS; in the event this particular association is of interest then further evaluation may be needed to optimize the analysis.

```
firthlogit blood hyperca i.ageband iss, nolog or
                                   Number of obs =   328
                                   Wald chi2(6) = 18.91
Penalized log likelihood = -31.960998              Prob > chi2   = 0.0043

--------------------------------------------------
     blood | Odds ratio   Std. err.      z   P>|z|     [95% conf. interval]
---------+----------------------------------------------------------------
   hyperca |  13.24669  10.47179   3.27  0.001    2.813288  62.37356
           |
   ageband |
    Infant |         1  (base)
   Toddler |  9.362346  15.51595   1.35  0.177    .3636746  241.0218
 Preschool |  4.599501  9.713592   0.72  0.470    .0732952  288.6329
    School |  2.917379  5.024827   0.62  0.534    .0997477   85.3263
Adolescent |  9.713027  15.76798   1.40  0.161    .403206   233.9819
           |
       iss |  1.025164  .0214162   1.19  0.234    .9840367   1.06801
```

Conclusion: there is evidence for association between hypercalcemia and prehospital blood administration, but the modeling requires further details due to separation problems (empty age band bins) and suggestion of potential confounding by both age and ISS.

Calcium administration

This analysis was not run in any detail. As would presumably be expected, the estimated association between hypercalcemia and prehospital calcium therapy was both significant (p <.001) and strong (OR 163).

Results: Quantile regression

Each of the following three sections presents the multivariable association between the endpoint of ICU LOS and calcium status (hypocalcemia, severe hypocalceamia, or hypercalcemia each evaluated in its own section).

Hypocalcemia

Hypocalcemia was not identified as a predictor of ICU LOS. The initial two discarded variables were male and PenMOI, with the next variable due to drop being hypocalcemia. The final model containing hypocalcemia is shown below. (Note that the Machado-Santos-Silva *p* value of <.001 was consistent with heteroskedasticity and a preference for using median regression rather than OLS regression.)

```
qreg2 piculos hypoca i.ageband iss

Median regression
R-squared = .09455785
Number of obs = 512
Objective function = 1.5792101

             Heteroskedasticity robust standard errors
---------------------------------------------------------------
   piculos | Coefficient Std. err.      t    P>|t|     [95% conf. interval]
-----------+---------------------------------------------------
    hypoca |  .1111111  .3829539   0.29  0.772  -.6412679  .8634901
           |
   ageband |
    Infant |         0  (base)
   Toddler |  .3333333  .6536689   0.51  0.610   -.950912   1.617579
 Preschool |  .4444444  .6358963   0.70  0.485  -.8048836   1.693773
    School |  .4444444  .5598776   0.79  0.428  -.6555318   1.544421
Adolescent |  .3333333  .5302759   0.63  0.530  -.7084853   1.375152
           |
       iss |  .1111111  .0191326   5.81  0.000   .0735217   .1487005
     _cons | -1.222222  .588629   -2.08  0.038  -2.378686  -.0657589
---------------------------------------------------------------

Machado-Santos Silva test for heteroskedasticity
       Ho: Constant variance
       Variables: Fitted values of piculos and its squares

       chi2(2)    = 46.782
       Prob > chi2 =  0.000
```

Conclusion: there is no evidence for association between hypocalcemia and median LOS (days) in the pediatric ICU.

Severe hypocalcemia

Severe hypocalcemia was not identified as a predictor of ICU LOS. The initial two discarded variables were male and PenMOI, with the next variable due to drop being hypocalcemia. The final model containing severe hypocalcemia is

shown below. (Note that the Machado-Santos-Silva *p* value of <.001 was consistent with heteroskedasticity and a preference for using median regression rather than OLS regression.)

Median regression
R-squared = .09381532
Number of obs = 512
Objective function = 1.5792101

Heteroskedasticity robust standard errors

piculos	Coefficient	Std. err.	t	P>|t|	[95% conf. interval]	
sevhypoca	-.5555556	.6683247	-0.83	0.406	-1.868595	.7574837
ageband						
Infant	0 (base)					
Toddler	.6666667	.6557687	1.02	0.310	-.6217041	1.955037
Preschool	1.222222	.6220287	1.96	0.050	.0001394	2.444305
School	.7777778	.578622	1.34	0.179	-.3590249	1.91458
Adolescent	.6666667	.5333755	1.25	0.212	-.3812416	1.714575
iss	.1111111	.0201012	5.53	0.000	.0716189	.1506034

Conclusion: there is no evidence for association between severe hypocalcemia and median LOS (days) in the pediatric ICU.

Hypercalcemia

After removal of male, PenMOI, and age band, the two remaining variables in the model were hypercalcemia and ISS. The results of this final model are shown below.

Median regression
R-squared = .08623838
Number of obs = 512
Objective function = 1.5767144

Heteroskedasticity robust standard errors

piculos	Coefficient	Std. err.	t	P>|t|	[95% conf. interval]	
hyperca	-1	.5489129	-1.82	0.069	-2.078414	.0784139
iss	.1111111	.0175136	6.34	0.000	.0767033	.1455189
_cons	-.7777778	.3492448	-2.23	0.026	-1.463917	-.091639

Machado-Santos Silva test for heteroskedasticity
Ho: Constant variance
Variables: Fitted values of piculos and its squares

chi2(2) = 37.363
Prob > chi2 = 0.000

Conclusion: there is no significant finding of association between hypercalcemia and median LOS (days) in the pediatric ICU. However, the non-significant *p* value approached (.069) – while not reaching – the threshold

Addendum in response to reviewer comments

Sensitivity analysis adjusting for injury patterns in patients with available AIS data

Given the reviewer's reasonable question about injury patterns, we have calculated our main results incorporating the available AIS data to generate three additional analyses. In the first analysis, we incorporate the AIS for the head region as a scored variable (from 0 to 6). In the second analyses, we incorporated a dichotomous definition for "any head injury" (any head AIS >0). In the third analysis, we classified cases as to whether the head injury was the main injury; this variable was coded as 1 if none of the other AIS regional scores exceeded that of the head AIS. The results from these three analyses are shown in the table below.

Analysis	Main reported result for hypercalcemia independent variable, OR (95% CI)	Results incorporating head AIS in three different ways		
		Head AIS as ordinal from 0-6, OR (95% CI)	Any head injury (AIS head >0), dichotomous, OR (95% CI)	Head injury is most severe injury (no other regional AIS exceeds head AIS)
Functional dependence	2.52 (1.45-4.35)	2.31 (1.04-5.11)	2.37 (1.08-5.21)	2.20 (1.01-4.81)
24-hour mortality	19.33 (6.96-53.69)	16.40 (4.16-64.59)	17.2 (4.25-69.64)	15.79 (4.05-61.53)
30-day mortality	9.07 (4.92-16.72)	12.16 (4.78-30.97)	11.73 (4.59-29.98)	12.34 (4.82-31.58)

**Abbreviations:* AIS – Abbreviated Injury Scale (score)

Exogenous calcium administration

While documentation of prehospital therapy was available for only part of the dataset (*n* = 328), in only six cases was calcium treatment initiated in the field. (With exclusion of those six cases, repetition of key findings did not suggest any substantive change in findings, as evidenced by the table below. The table reproduces the methods of the main results (including primary reporting of Firth penalized regression results for the 24-hour mortality endpoint).

Analysis	Main reported result for hypercalcemia independent variable, OR (95% CI)	Result excluding cases with prehospital calcium, OR (95% CI)
Functional dependence	2.52 (1.45-4.35)	2.53 (1.45-4.43)
24-hour mortality	19.33 (6.96-53.69)	22.27 (7.60-65.24)
30-day mortality	9.07 (4.92-16.72)	9.90 (5.31-18.48)

**Abbreviations:* CI – confidence interval, OR – odds ratio

Discrimination and calibration

Analysis	Receiver operator characteristic curve area (95% confidence interval)	Calibration-in-the-large	Calibration slope
Functional dependence	0.73 (0.68-0.77)	0.00 (-0.21 to 0.20)	1.01 (0.74-1.28)
24-hour mortality	0.78 (0.68-0.88)	-0.11 (-0.53 to 0.31)	1.04 (0.71-1.37)
30-day mortality	0.78 (0.72-0.85)	-0.02 (-0.31 to 0.27)	1.01 (0.76-1.26)

Calibration plot for hypercalcemia and functional outcome

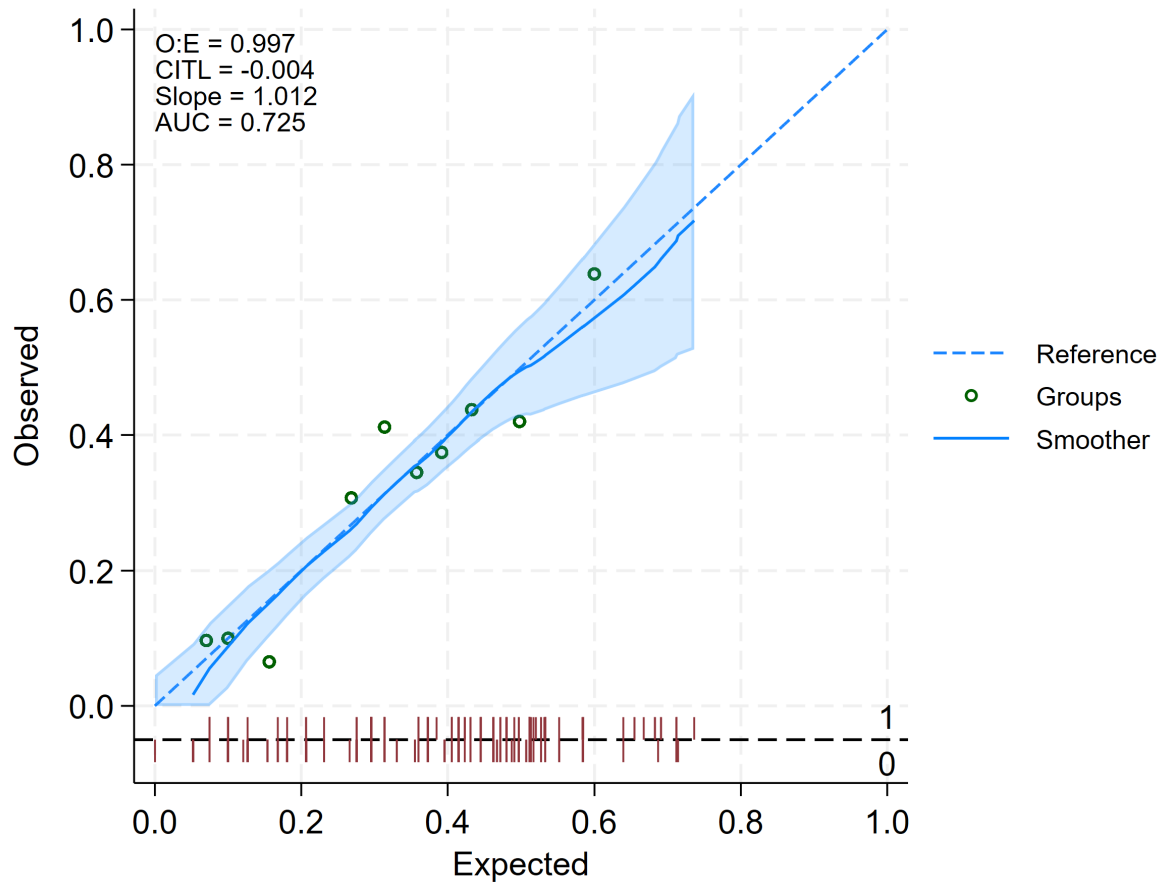

Calibration plot for hypercalcemia and 24-hour mortality

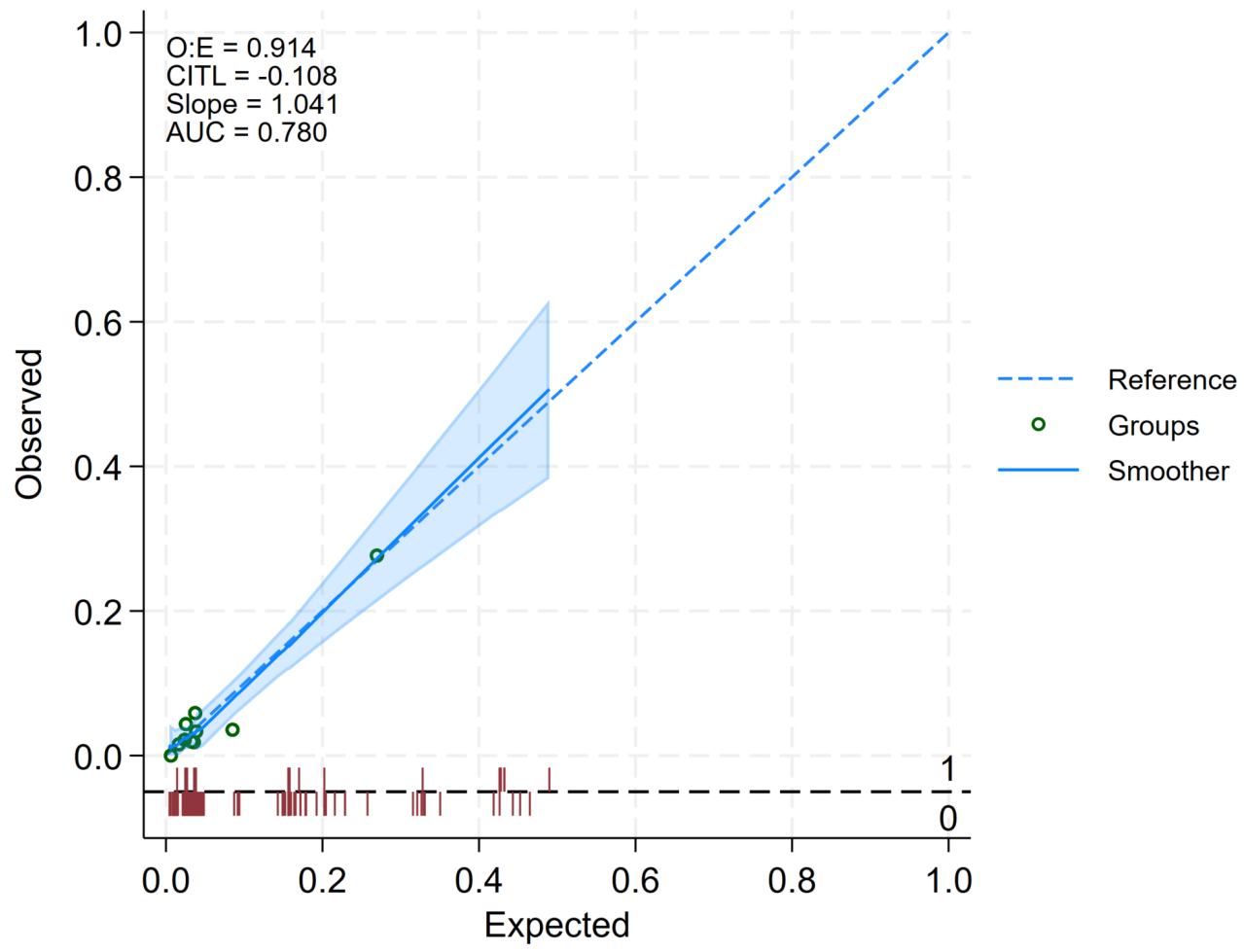

Calibration plot for hypercalcemia and 30-day mortality

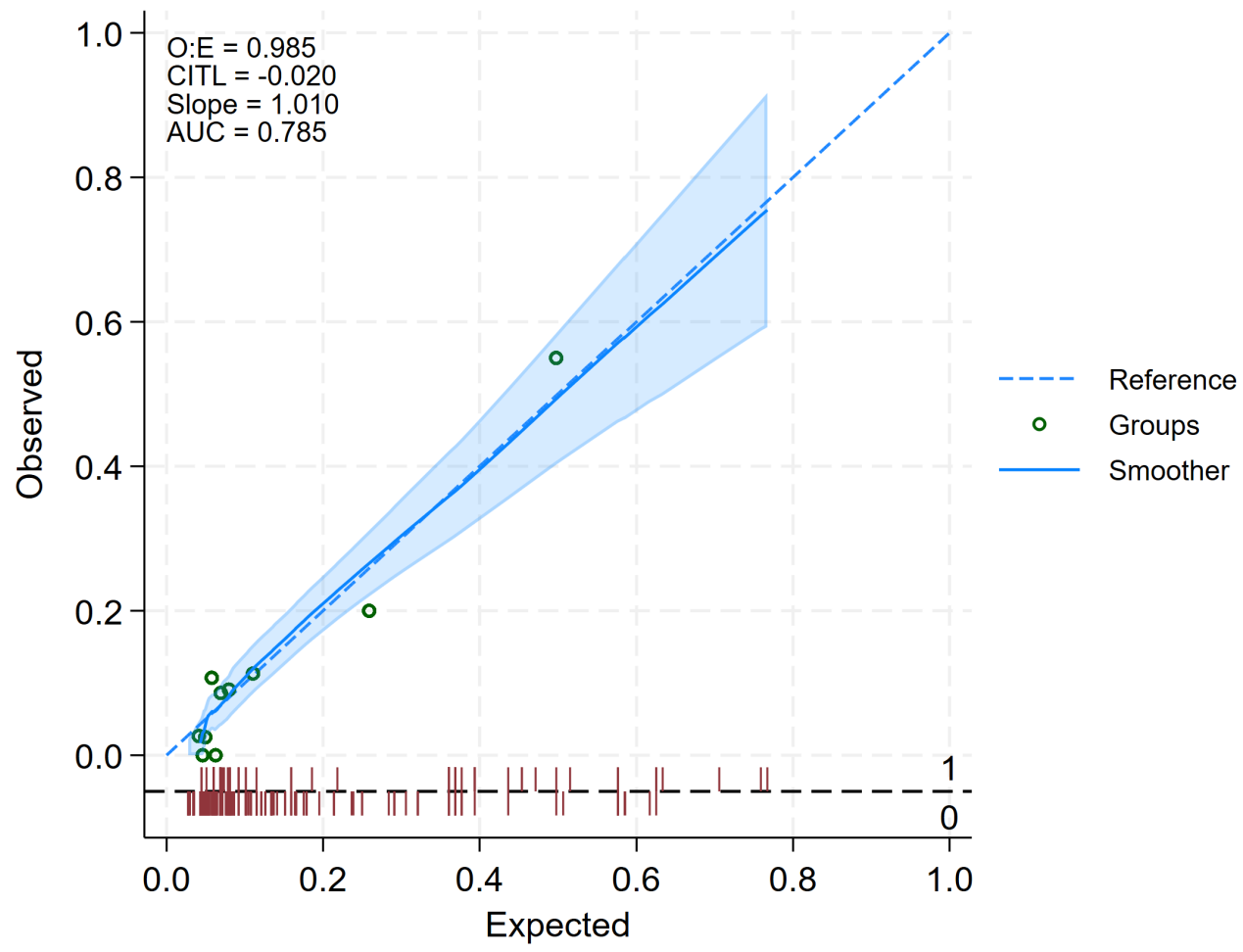

Sensitivity analysis for missing functional outcome

The model for dependent functional outcome utilized a smaller portion of the overall dataset: of the overall *n* of 517 the functional outcome utilized 488 patients. However, nearly all of the model's missingness was due to missing functional outcome in 27 cases (5.22% of 517). With removal of these cases due to lack of ascertainment of the functional status, there were only two other cases dropped due to covariate (ISS) missingness. Since we do not execute MICE in either the primary independent variable (hypercalcemia, with 0% missingness) or the outcome (functional status), we judged there was no role for MICE for these data. We have added a sensitivity analysis that shows the results of replacing all missing functional outcome cases as either having poor functional outcome or good functional outcome; results are shown along with the original results in the table below and indicate little substantive change in the effect estimate (with 95% CIs that overlap with each other and the main results, and which exclude the null value).

**Main independent variable**	**Main results OR (95% CI) for hypercalcemia indepdendent variable in estimation sample *n* = 488**	**Replacement of cases missing functional outcome with either favorable or non-favorable status**	
		**Replacement with unfavorable status, OR (95% CI)**	**Replacement with favorable status, OR (95% CI)**
Hypercalcemia	2.52 (1.45-4.35)	2.82 (1.68-4.71)	2.49 (1.45-4.29)

**Abbreviations:* CI – confidence interval, OR – odds ratio

Age-based definitions of ionised hypercalcaemia

Our goal with this sensitivity analysis was to determine whether our main findings of association of hypercalcemia with untoward outcomes were nullified when we used an age-based definition of hypercalcemia. The findings were not substantively moved towards the null when we utilized age-based definitions for hypercalcemia. The table below shows the findings after we changed *n* = 27 cases' from "hypercalcemia" to "no hypercalcemia;" of these 27, 19 were infants, 6 were toddlers, and 1 each were in the school-age and adolescent groups.

Analysis	Main reported result for hypercalcemia independent variable, OR (95% CI)	Result obtained when utilizing age-based varying reference for hypercalcemia, OR (95% CI)
Functional dependence	2.52 (1.45-4.35)	3.39 (1.72-6.67)
24-hour mortality	19.33 (6.96-53.69)	27.65 (10.17-75.23)
30-day mortality	9.07 (4.92-16.72)	12.63 (6.28-25.40)

**Abbreviations:* CI – confidence interval, OR – odds ratio

Time from injury to calcium measurement

We evaluated the three hypercalcemia endpoints in models that incorporated time from injury to calcium assessment. In none of the three models was the time from injury to calcium statistically significant, although the evaluation for the functional outcome approached significance (time from injury to calcium assessment p = .069). For the two mortality endpoints the time from injury to calcium assessments were both >.9. As shown in the table below, there was no substantive change in hypercalcemia effect estimates for any of the three outcomes; the directionality of movement when incorporating time from injury to calcium assessment was away from the null.

Analysis	Main reported result for hypercalcemia independent variable, OR (95% CI)	Result obtained when adding time from injury to calcium assessment as an additional covariate, OR (95% CI)
Functional dependence	2.52 (1.45-4.35)	2.65 (1.48-4.78)
24-hour mortality	19.33 (6.96-53.69)	19.47 (6.95-54.58)
30-day mortality	9.07 (4.92-16.72)	10.85 (5.62-20.93)

**Abbreviations:* CI – confidence interval, OR – odds ratio

Supplement: Interpreting Stata output

CI output

For CIs: in the example below there were **13880** observations, with the proportion of 17.9%, having a 95% CI of **17.2-18.6%.**

```
. cii prop 13880 2485
                                        -- Binomial Exact --
   Variable |        Obs  Proportion   Std. Err.      [95% Conf. Interval]
-------------+----------------------------------------------------------------
            |     13,880   .1790346    .0032541       .1726883    .1855157
```

Ordinal or continuous descriptives

In the example below the median age is **58** with overall range of 1-89 and IQR of **43-71**. Other cutoffs are (for example): 95% of cases were at least **87** years old. The mean is **56.6** with **SD 19.0**.

```
                    Age
-------------------------------------------------------------
      Percentiles      Smallest
 1%          16             1
 5%          24             1
10%          29             1       Obs              13,822
25%          43             1       Sum of Wgt.      13,822

50%          58                     Mean           56.55347
                        Largest     Std. Dev.      19.03476
75%          71            89
90%          82            89       Variance        362.322
95%          87            89       Skewness      -.2508046
99%          89            89       Kurtosis       2.330527
```

Epidemiologic tables

When the rare-disease assumption is violated, Stata's cohort study command is used to generate (univariable) calculations for risk ratio with its **95% CI**, as a metric for relative risk. The same command procedure is useful to calculate NNT (or number needed to harm, etc.); the NNT is simply the inverse of the **risk difference** (thus the NNT for the table below would be 1/.27 or 3.7 (its 95% CI be similarly calculated by inverting the **95% for risk difference**). The Stata command also produces a **chi-square *p*** (and some other output which is not reproduced here as it's nearly always ignored).

```
                 | Exposed   Unexposed |      Total
-----------------+----------------------+-----------
           Cases |        20         77 |         97
        Noncases |         7         85 |         92
-----------------+----------------------+-----------
           Total |        27        162 |        189
                 |                      |
            Risk |  .7407407   .4753086 |   .5132275
                 |                      |
```

| Point estimate | [95% conf. interval]

```
       |----------------+----------------------------
Risk difference |      .2654321    |  .0831219 .4477423
    Risk ratio |      1.558442    |  1.183001   2.053033
       +-------------------------------------------------------
                chi2(1) =    6.53  Pr>chi2 = 0.0106
```

Estimating median differences between groups (non-normal data)

One common situation entails finding non-normal continuous (or ordinal) data, for which we wish to determine the median difference (with its 95% CI). Stata output in this regard is depicted below: the median difference between groups is highlighted in **blue** with its 95% CI highlighted in **yellow**. (Note that the sign depends on how the groups are numbered; in this case a preceding "summarize" command estimated the medians to be 37 for normal subjects and 64 for COVID subjects; this aids in interpreting the sign of the output.)

```
Y-variable: emean (Emean)
Grouped by: dx
Group numbers:

      dx |     Freq.   Percent      Cum.
--------+  -----------------------------------
   Normal |     1,200     40.36     40.36
    COVID |     1,773     59.64    100.00
--------+---------------------------------------
     Total |     2,973    100.00
Transformation: Fisher's z
95% confidence interval(s) for percentile difference(s)
between values of emean in first and second groups:
  Percent   Pctl_Dif   Minimum     Maximum
       50  -21.799999      -25  -18.779999
```

t test

The *t* test output allows evaluation of both the *p* value and also the between-groups point estimate (with its 95% CI). (The sign of the between-groups differences is interpreted based on looking up at the means in the output to see which gorup's mean was greater.)

In the output below there is no difference **(p = .705)** between **means of Blind and US-guided groups' ages**. The point estimate for the intergroup difference in ages is **-1.9** with 95% CI of **minus 12.3 to plus 8.4.**

```
Two-sample t test with equal variances
--------------------------------------------------------
  Group |     Obs      Mean   Std. err.  Std. dev.  [95% conf. interval]
------+-----------------------------------------------------------------------
  Blind |     21  65.47619   3.411199   15.63208   58.36055   72.59183
 USGuide |     19  67.42105   3.822307   16.66105   59.39068   75.45142
------+-----------------------------------------------------------------------
Combined |     40      66.4   2.521904   15.94992   61.29897   71.50103
------+-----------------------------------------------------------------------
   diff |        -1.944862   5.106405              -12.28224   8.392514
--------------------------------------------------------
   diff = mean(Blind) - mean(USGuide)                 t = -0.3809
H0: diff = 0                              Degrees of freedom =   38
```

Ha: diff < 0	Ha: diff != 0	Ha: diff > 0
Pr(T < t) = 0.3527	Pr(|T| > |t|) = **0.7054**	Pr(T > t) = 0.6473

Logistic regression

For logistic regression output tables: in the output below, the outcome is **death**. If the logistic regression is **clustered** the number of clusters is indicated below the pseudo-R^2 (which result is usually ignored). For a given predictor variable, each line gives its OR, its Wald *p* value (which *may not* be the ultimate *p* value measure used), and the 95% CI for the OR. In
the example below: for the predictor “ACE/ARB” the OR was **0.75** (95% CI **.59-.94**, *p* = **.01**).

```
Logistic regression                 Number of obs   =    11,555
                            Wald chi2(14)   =  1783.24
                            Prob > chi2       =    0.0000
Log pseudolikelihood = -3807.0284          Pseudo R2       =    0.2030

                  (Std. Err. adjusted for 109 clusters in hospital)
--------------------------------------------------------------
        |          Robust
   died | Odds Ratio  Std. Err.     z   P>|z|     [95% Conf. Interval]
--------+-----------------------------------------------------
  acearb |  .7453943  .0863024  -2.54  0.011     .594064   .935274
 quarter |  .6946281  .0712361  -3.55  0.000    .5681451  .8492694
     age |  1.075208  .0031866  24.47  0.000     1.06898  1.081472
    male |   1.55701  .0802553   8.59  0.000    1.407397  1.722527
```

Output for other modeling is generally the same as for the example above.

References

1. Hosmer DW, Lemeshow S, Sturdivant RX. Applied logistic regression. Wiley series in probability and statistics 398. Third edition / ed. Hoboken, New Jersey: Wiley,; 2013. p. 1 online resource.

2. Schmidt CO, Kohlmann T. When to use the odds ratio or the relative risk? *Int J Public Health* 2008; **53**(3): 165–7.

3. McNutt LA, Wu C, Xue X, Hafner JP. Estimating the relative risk in cohort studies and clinical trials of common outcomes. *Am J Epidemiol* 2003; **157**(10): 940–3.

4. Senaviratna N, Cooray T. Diagnosing multicollinearity of logistic regression model. *Asian J Probab Statistics* 2019; **5**(2): 1–9.

5. Hardin JW, Hilbe JM. Generalized linear models and extensions. 4 ed. College Station, TX: Stata Press; 2018.

6. Ensor J, Kym I, Martin E. PMCALPLOT: Stata module to produce calibration plot of prediction model performance. Statistical Software Components. rev. 4 Jan 2020 ed. Boston, MA: Boston College Department of Economics; 2018.

7. Miller ME, Hui SL, Tierney WM. Validation techniques for logistic regression models. *Stat Med* 1991; **10**(8): 1213–26.

8. Steyerberg EW, Vickers AJ, Cook NR, et al. Assessing the performance of prediction models: a framework for traditional and novel measures. *Epidemiology* 2010; **21**(1): 128–38.

9. Mitchell MN. Interpreting and visualizing regression models using Stata. 2nd edition. ed. College Station, Tex.: Stata Press; 2021.

10. van Smeden M, de Groot JA, Moons KG, et al. No rationale for 1 variable per 10 events criterion for binary logistic regression analysis. *BMC Med Res Methodol* 2016; **16**(1): 163.

11. Ding R, McCarthy ML, Desmond JS, Lee JS, Aronsky D, Zeger SL. Characterizing waiting room time, treatment time, and boarding time in the emergency department using quantile regression. *Academic emergency medicine : official journal of the Society for Academic Emergency Medicine* 2010; **17**(8): 813–23.

12. Das K, Krzywinski M, Altman N. Quantile regression. *Nat Methods* 2019; **16**(6): 451–2.

13. Machado JA, Parente PM, Santos-Silva JM. QREG2: Stata module to perform quantile regression. *Statistical Software Components* 2011; **S457369**.

14. Machado JA, Santos-Silva JM. Glejser's test revisited. *J Econometrics* 2000.
