# Supplementary material for: Admission ionised calcium disturbances and adverse outcomes in paediatric major trauma: a UK multicentre retrospective cohort study
Source: BMJ Open. 2026 Sep 18;16(9):e122447. doi: 10.1136/bmjopen-2026-122447 (PMC13599871; doi:10.1136/bmjopen-2026-122447)
Supplement: Supplementary file 3 [file bmjopen-16-9-s004.pdf]

COWS
Paediatric Trauma Research

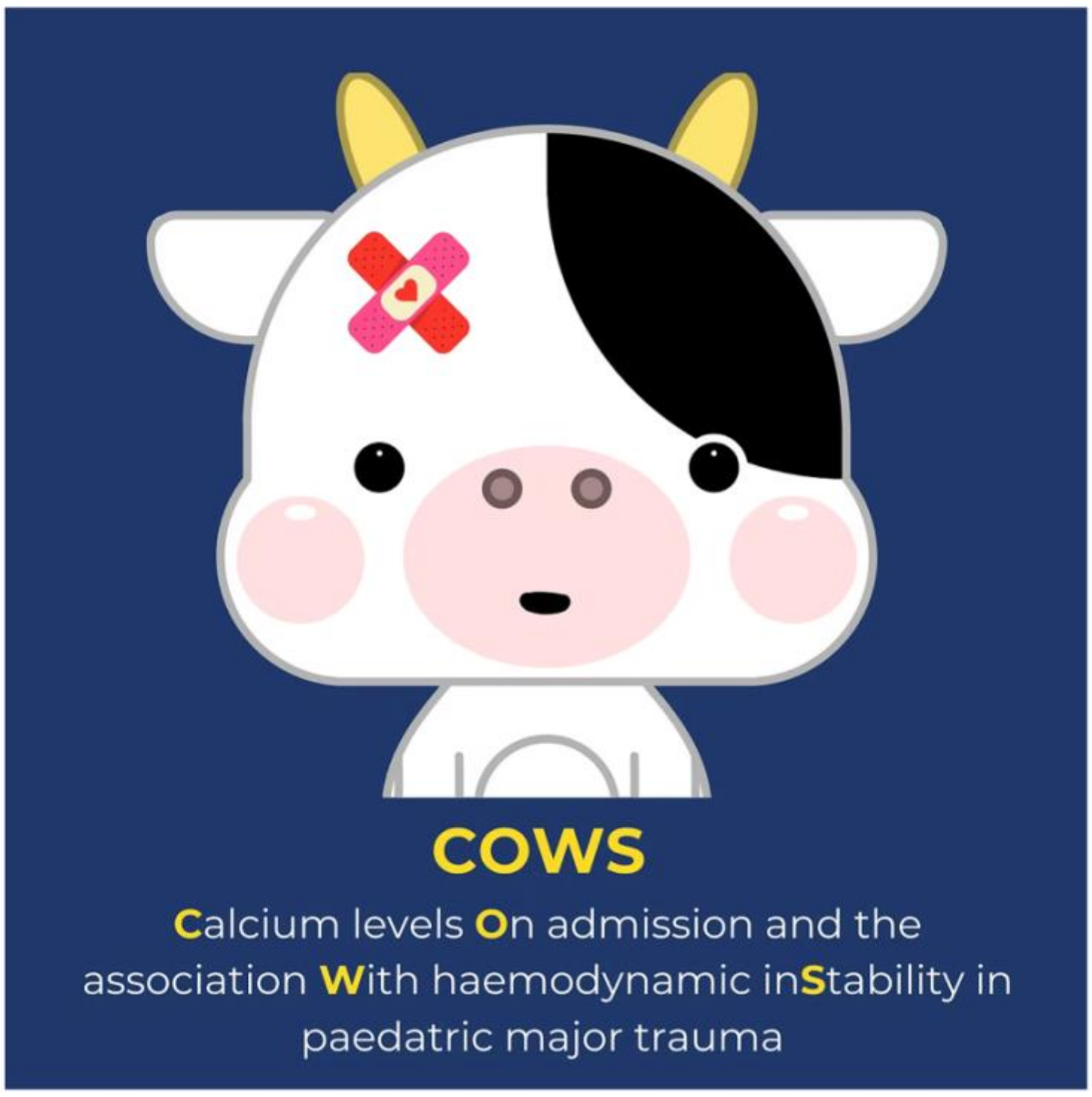

Why?
Why is this important?

How?
How are we looking into this?

What?
What data are we looking at?

Why is this study important?

Serious bleeding can occur following injury

Significant injuries are still one of the leading causes of death and disability in children and young people. Many of the deaths that occur following an injury are due to severe bleeding. Recognising and controlling bleeding and the factors within the body which make it worse is key to reducing preventable deaths from injury.

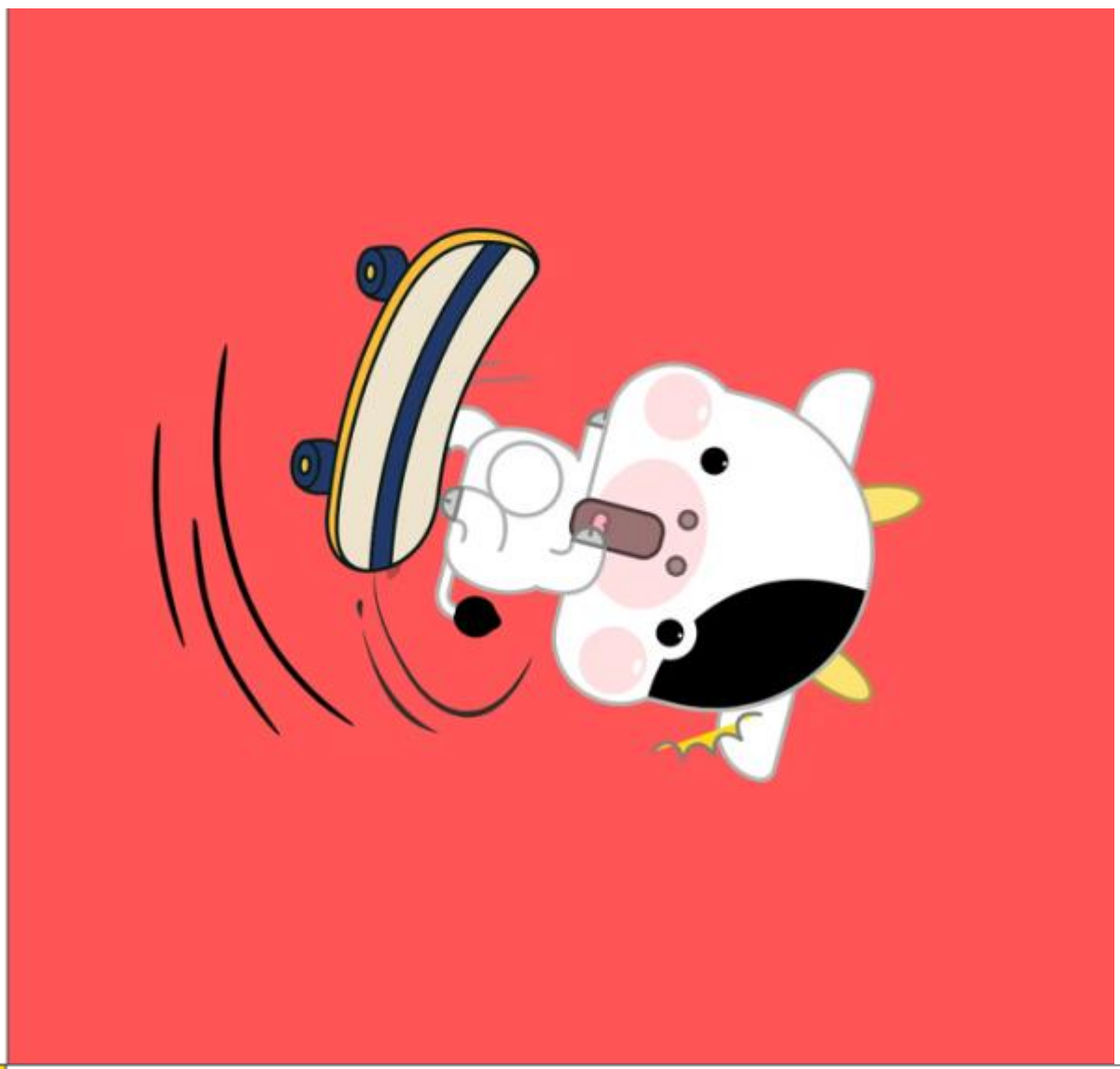

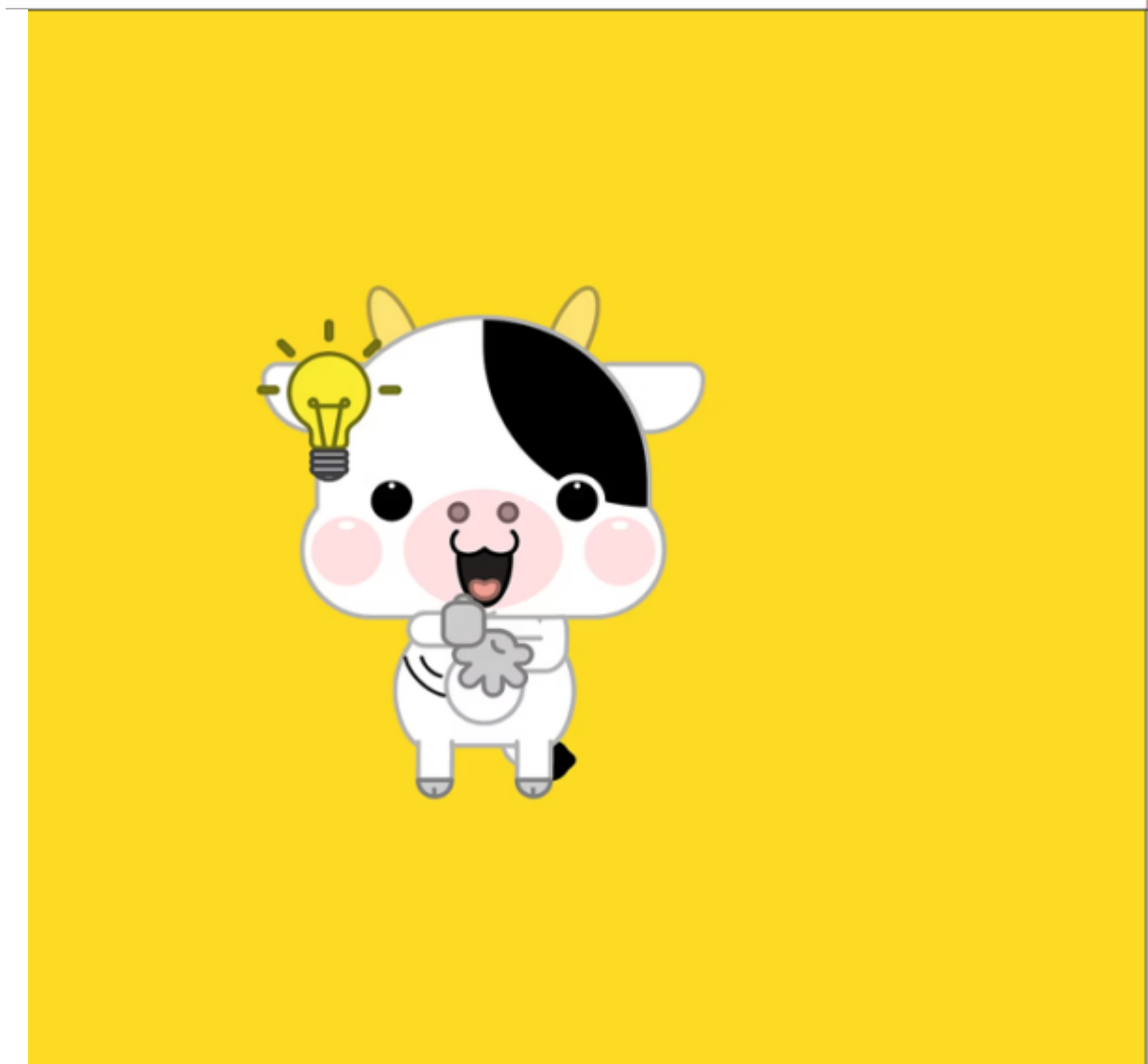

Calcium is important for the body

Calcium helps blood to clot, the heart to beat stronger, and blood vessels to better respond to blood loss.

Calcium might be low in injured people

Low calcium levels have been seen in injured adults. This might be due to the body's response to the injury, some of the treatments we give in the early stages of injury treatment, or a combination of both.

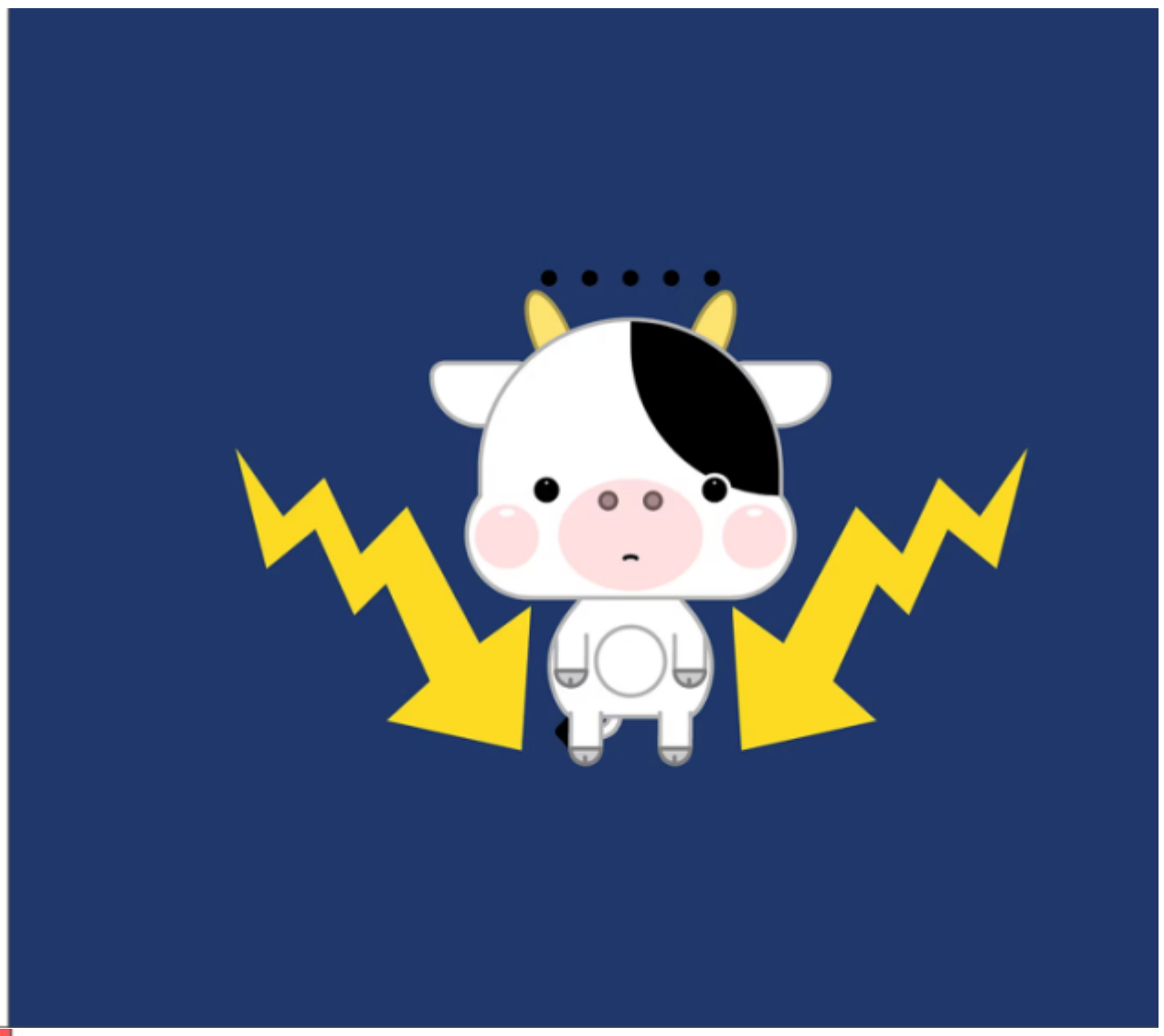

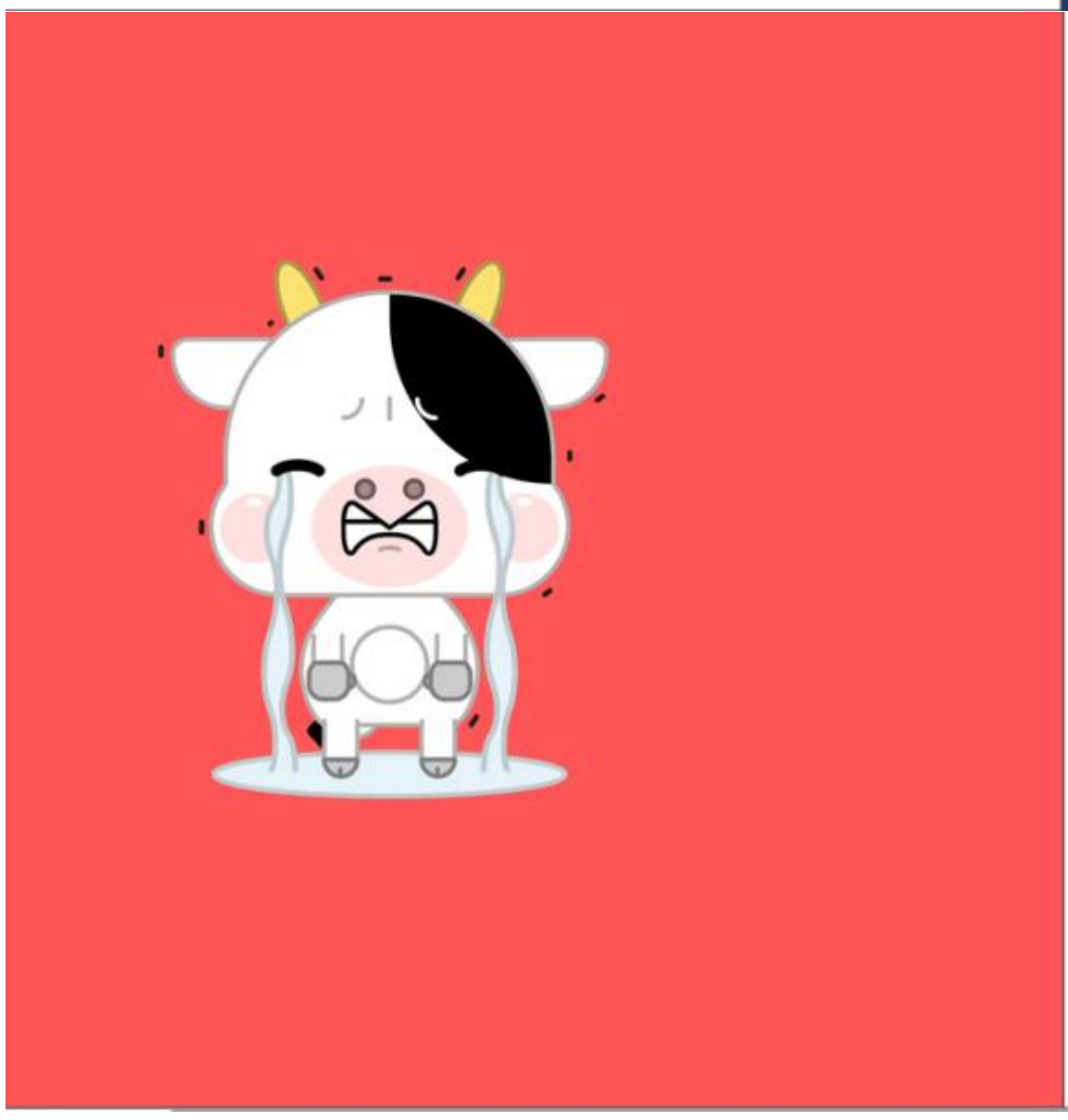

Low calcium might be detrimental in injured people

Amongst injured adults, low calcium appears to be associated with a more difficult injury journey, needing bigger treatments such as blood transfusions or operations, and having an increased chance of disability and death. We don't know if the same is the case amongst injured children and young people.

How are we looking into this?

Reviewing historical data

Archived data from 2016 to 2023 will be examined. This means that the event has already occurred, and the data has already been recorded as part of the care received at the time.

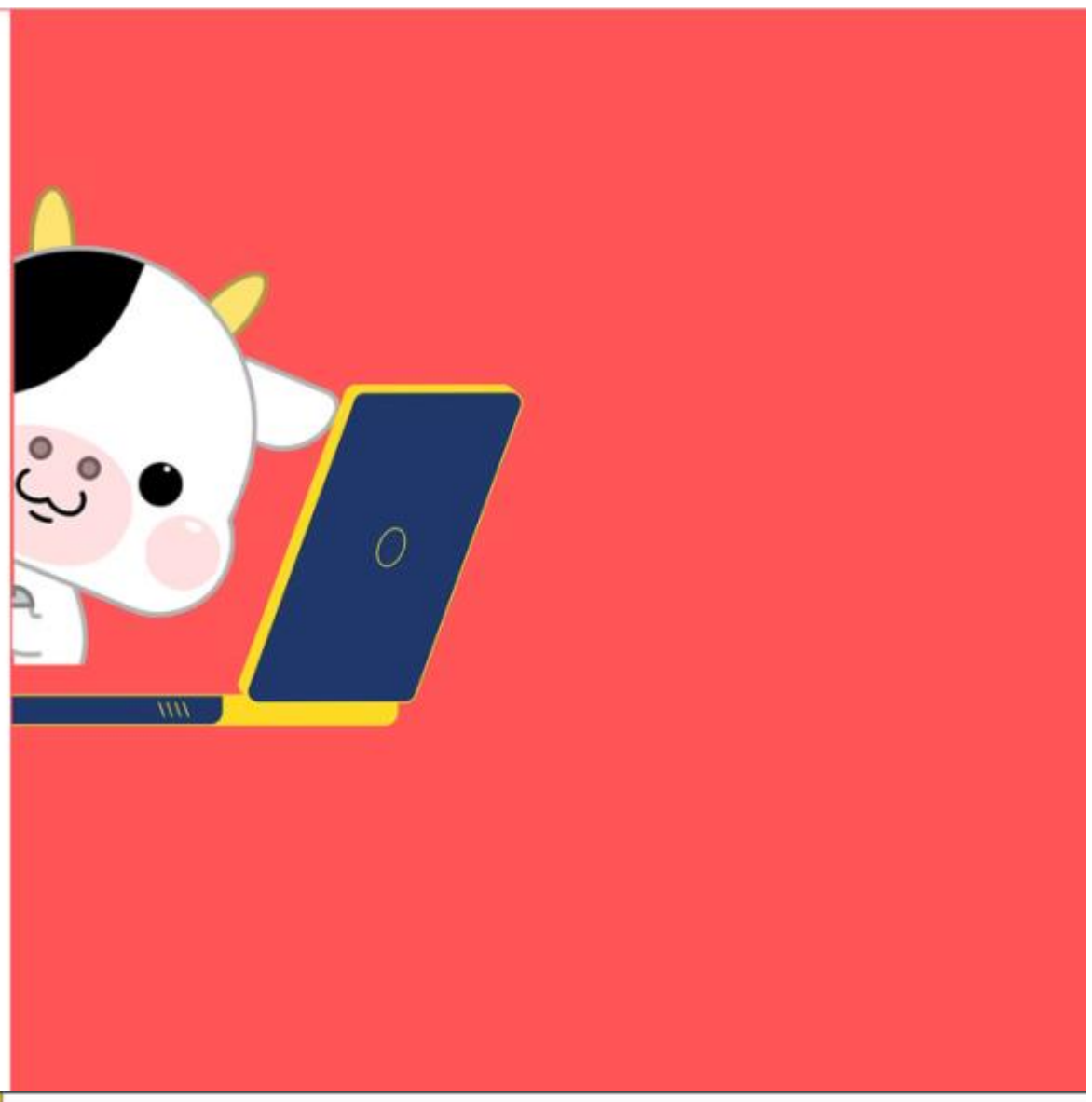

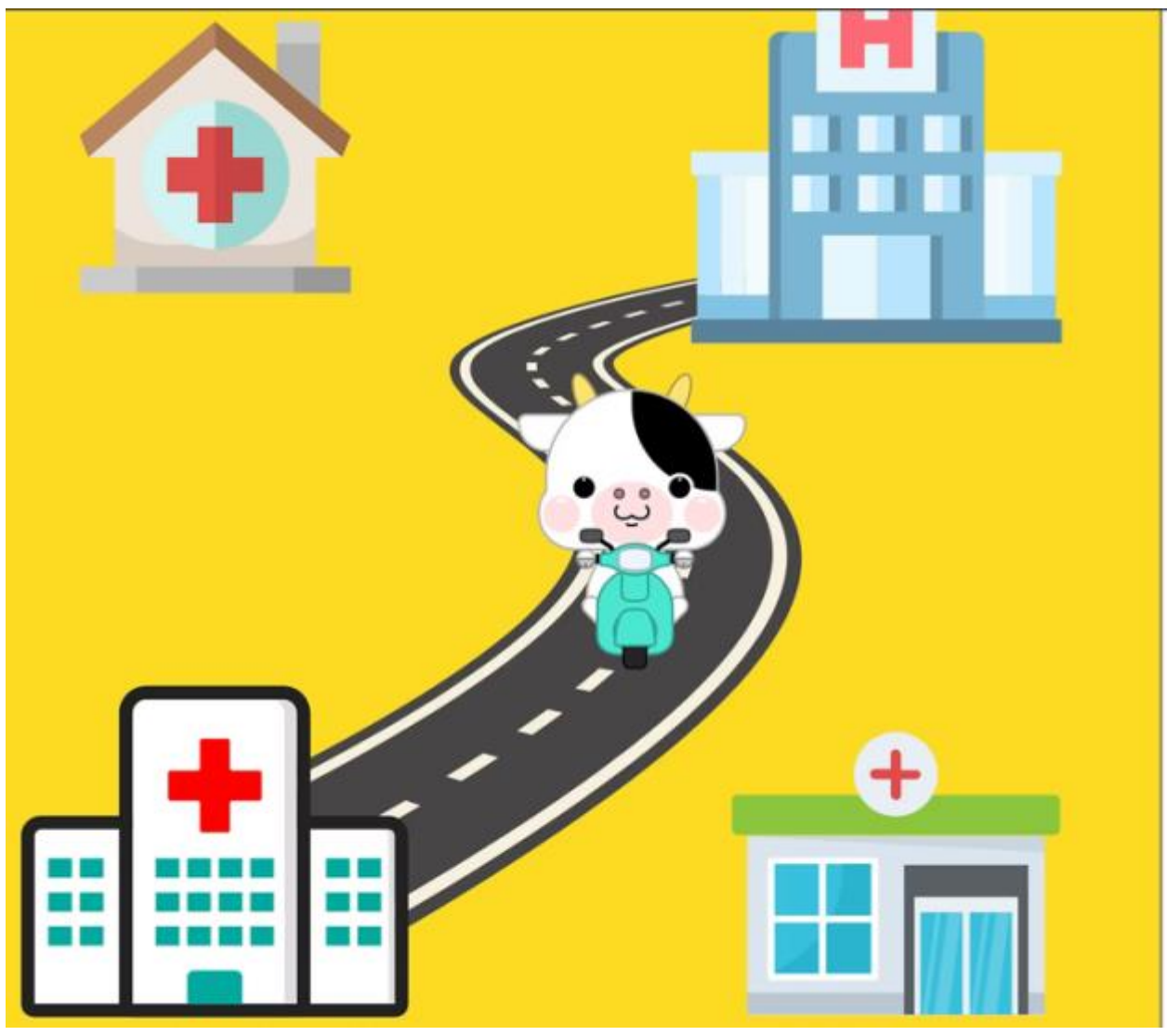

Using data from multiple hospitals

To explore the question properly, the study needs to include approximately 600 children and young people. This means that data from multiple hospitals will be used to achieve these numbers and to ensure the results aren't just a reflection of how children and young people are treated at one particular hospital.

The hospital teams will anonymise all data

The local hospital teams will review the data before passing it on to the research teams. This involves going through a process of anonymising the data. This includes removing any identifiable details, and also involves providing only the information that is relevant to answering the research question.

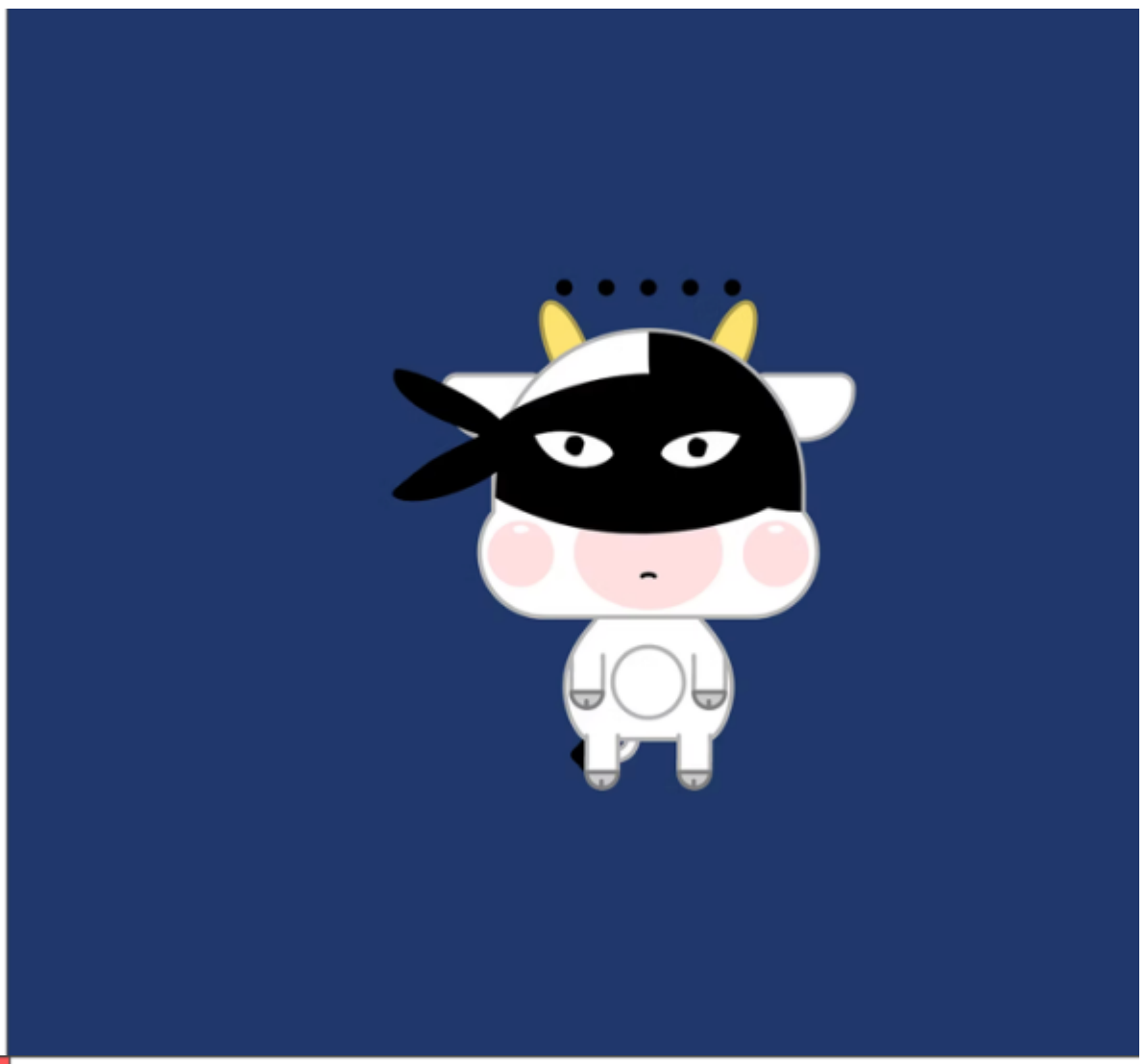

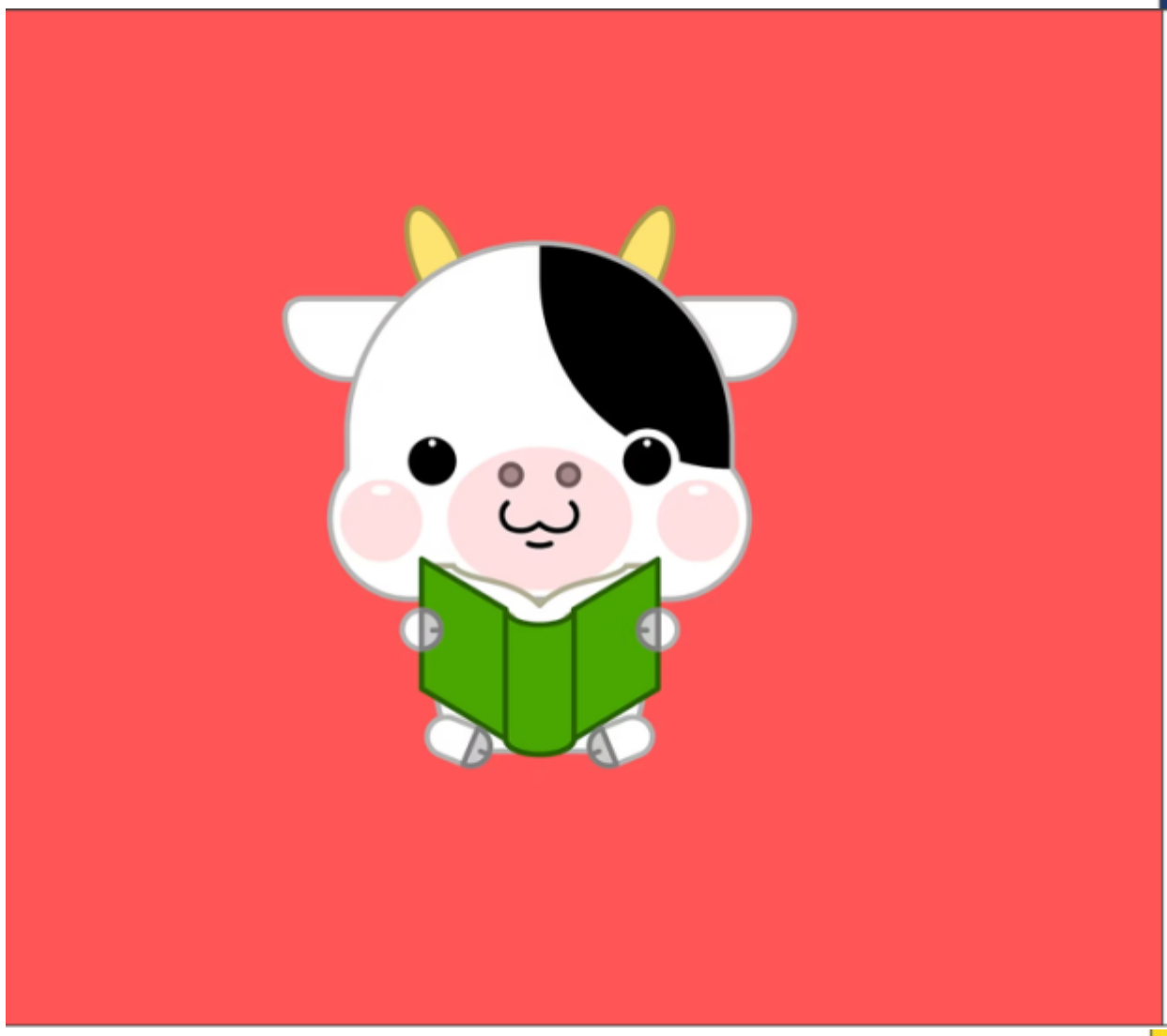

The researchers won't be able to identify any individuals

The researchers will not be able to identify any individual patients. Even if the injury was so rare or unusual that it made the news, the data will be anonymised in a way that the researchers will not be able to link this to the data.

Anonymous data will be pooled to try and answer the research question

In addition to the data being anonymised, it will also be pooled together to answer the question. For example, how many children and young people have low calcium levels? What proportion with low calcium had a low blood pressure compared to those with normal calcium levels?

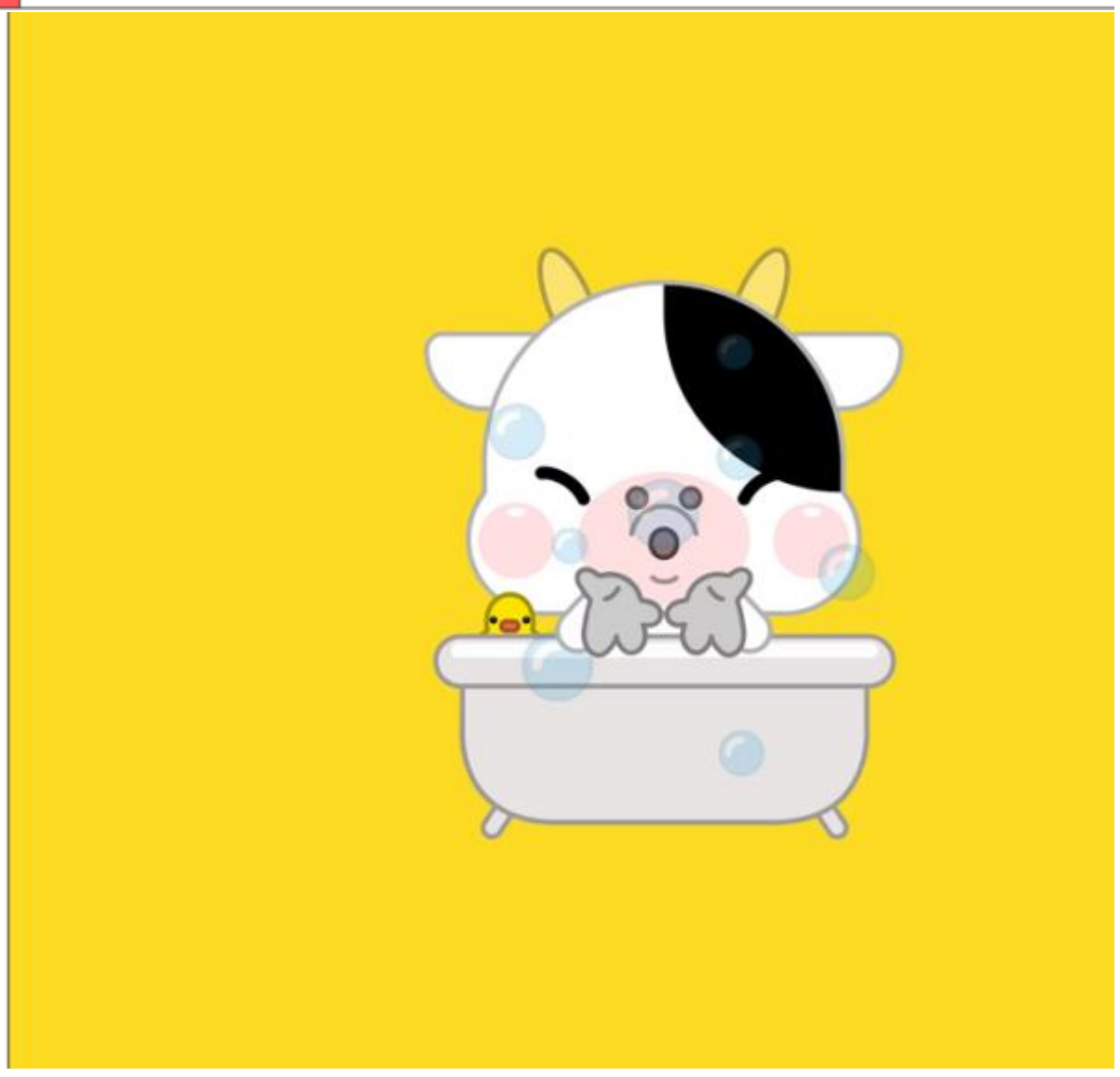

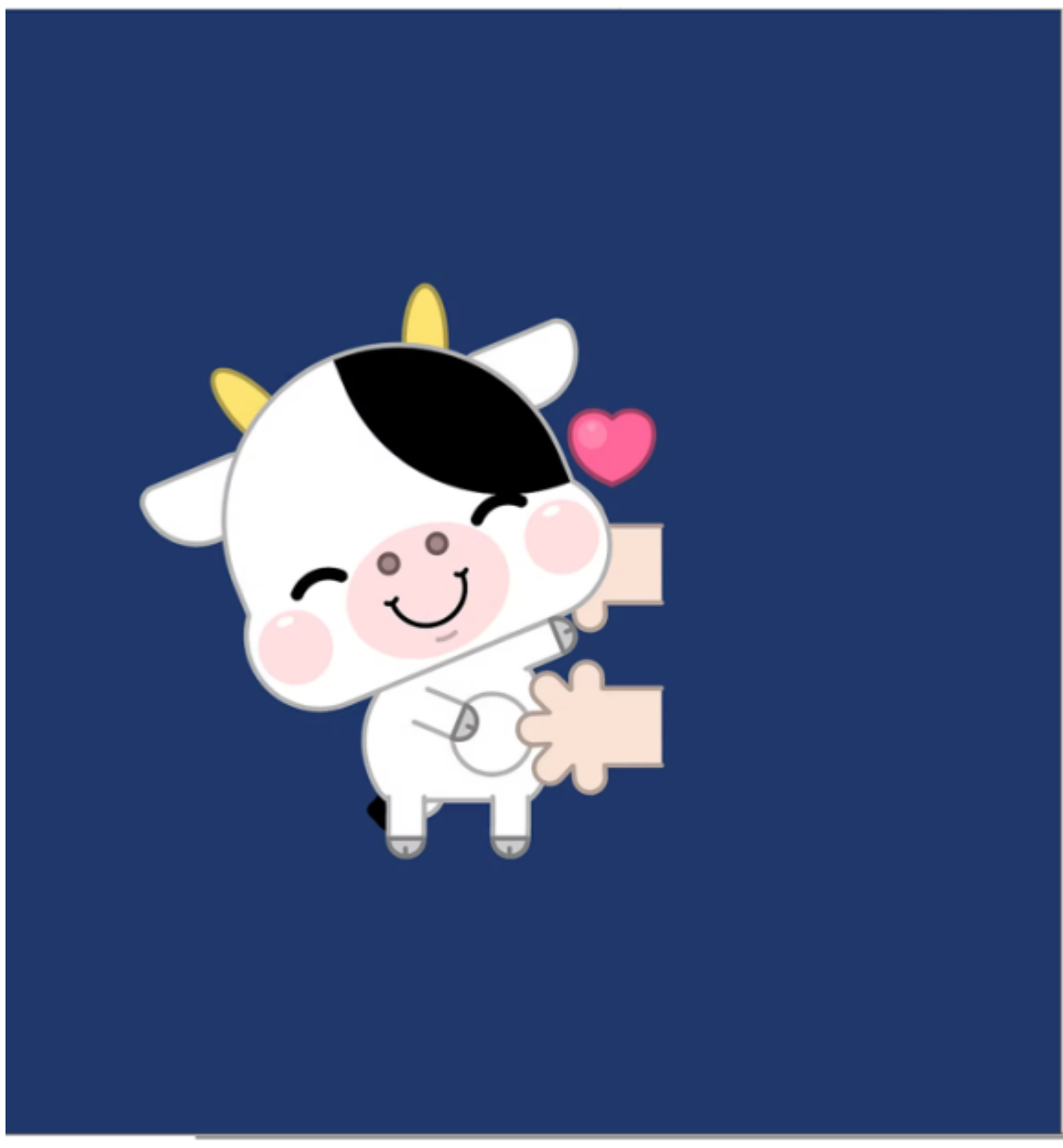

Findings will be published to help improve our understanding of this question

Publishing findings allows other researchers to learn from the results. This might change how we understand calcium in injured children and young people and might help us to think about how we can improve their care.

What data are we looking at?

The local teams anonymise all data before passing it on to the research team, who will then pool and analyse the data. Anticipating that some injuries (like shootings or stabbings) might be identifiable from the event itself, further steps have been taken to ensure the anonymity of data.

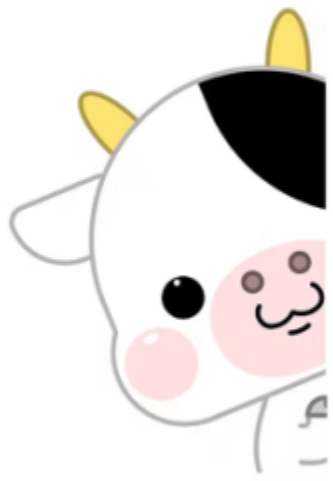

Patient details

The researchers will not have access to any identifiable patient data. Gender will be recorded as male, female, or other. Age will be broken down into categories rather than specific ages.

Injury details

The reason for the injury will be recorded either as blunt or penetrating rather than giving specific mechanisms (such as a car crash or stabbing). The severity of the injury will be split into categories based on a standard scoring system. Although the time of the injury won't be recorded, the local teams will let the research team know some important time intervals, such as how long it took to get to the hospital or how long it took to measure the calcium level.

Vital signs

Vital signs include a patient's blood pressure, heart rate, breathing rate, and oxygen levels. Abnormal vital signs can be a measure of how unwell a child or young person might be. The researchers will look at whether vital signs are abnormal and how abnormal these are.

Blood tests

Blood tests will include the calcium level and other factors that may be associated with worse bleeding or a consequence of bleeding.

Outcomes

The researchers will examine what treatments are needed, such as blood transfusions or operations. They will also look to see if the low calcium levels are associated with disability or death.
