# Supplementary material for: Admission ionised calcium disturbances and adverse outcomes in paediatric major trauma: a UK multicentre retrospective cohort study
Source: BMJ Open. 2026 Sep 18;16(9):e122447. doi: 10.1136/bmjopen-2026-122447 (PMC13599871; doi:10.1136/bmjopen-2026-122447)
Supplement: Supplementary file 4 [file bmjopen-16-9-s005.pdf]

PAEDIATRIC RESEARCH

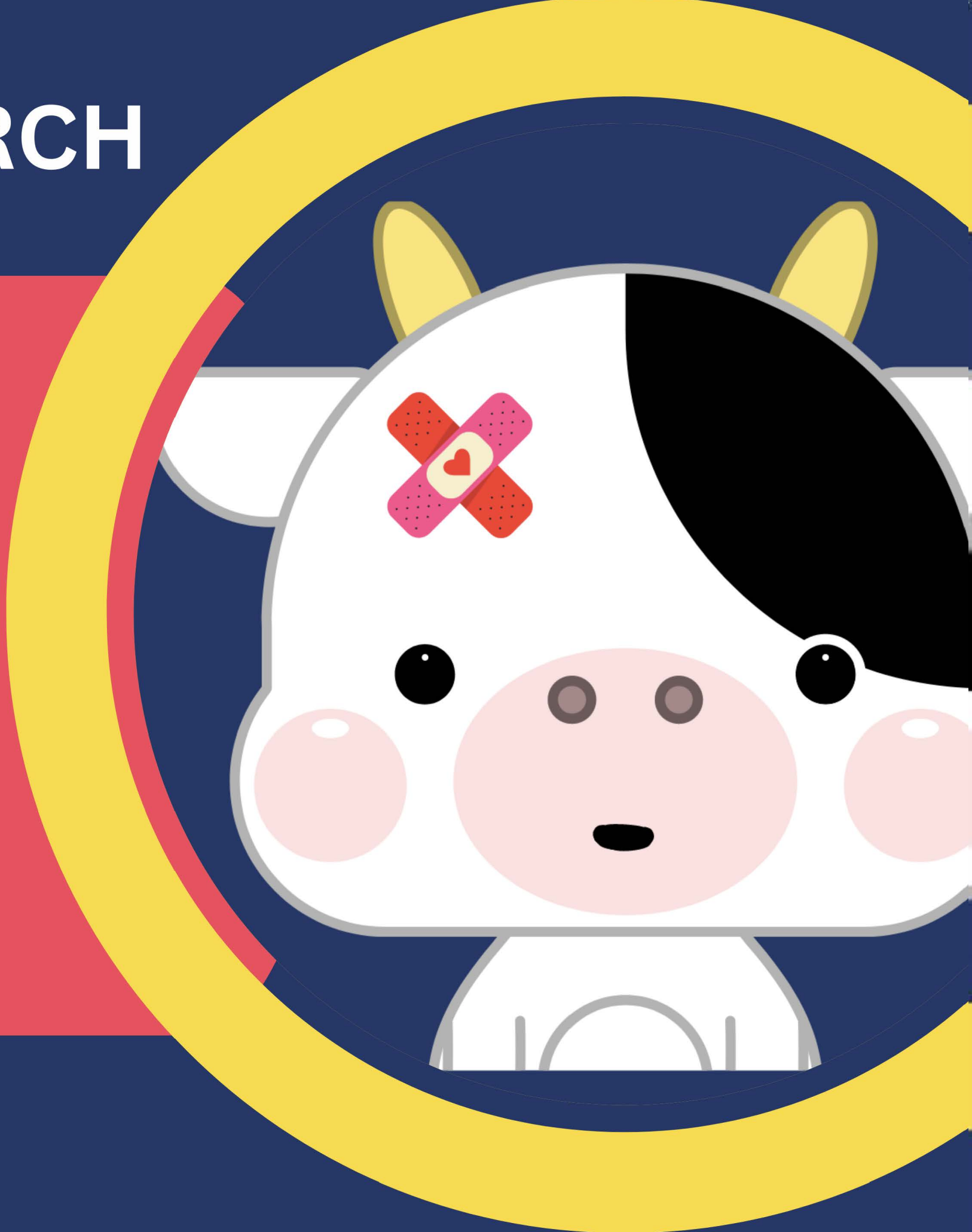

COWS

Calcium levels **O**n admission and the association **W**ith haemodynamic in**S**tability in paediatric major trauma

WHY ARE WE WORRIED ABOUT TRAUMA?

- Serious injuries, known as major trauma, are one of the most common reasons for young people to die. Many of the deaths that occur in trauma are as a result of bleeding.
- Recognising and controlling bleeding and the factors within the body which make it worse is key to reducing preventable deaths from major trauma.

WHY IS CALCIUM IMPORTANT?

- Calcium helps blood to clot, the heart to beat stronger, and blood vessels to better respond to blood loss.
- Calcium might be low in injured people. We know this happens in adults and is associated with being more unwell, needing more treatments, and having worse outcomes.
- We don't know if the same happens in young people.

WHAT ARE WE LOOKING AT?

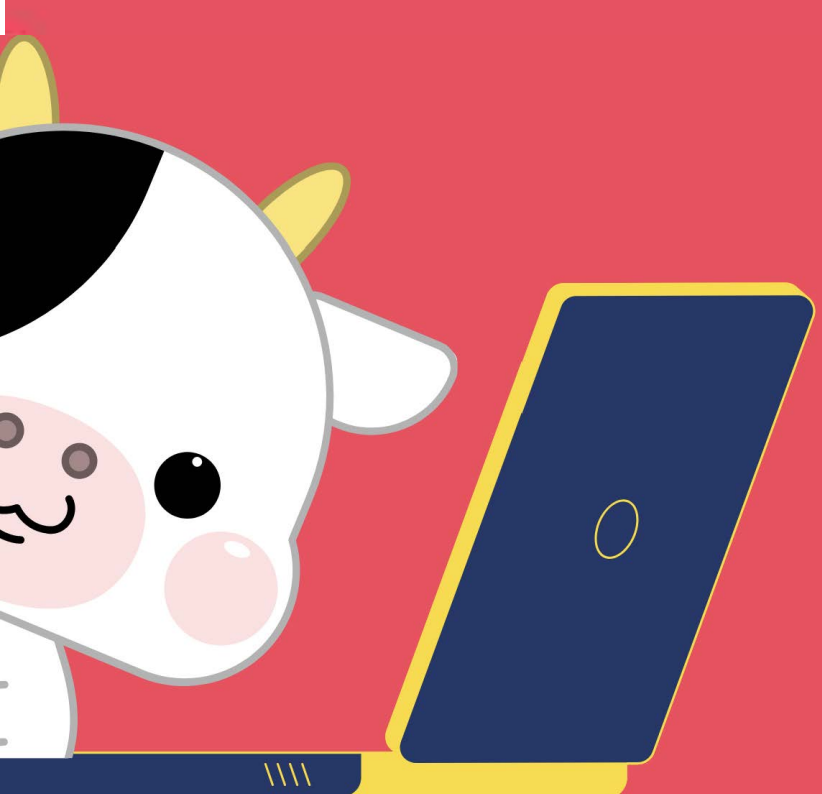

- Archived data from injured young people (<16yrs) from 2016-2023 from different hospitals.
- All data will be completely anonymised by the local hospitals before the research team has access to the data.
- How common are low calcium levels? Is this linked with a more difficult injury journey, needing bigger treatments, and having an increased chance of disability and death?
