# Supplementary material for: Admission ionised calcium disturbances and adverse outcomes in paediatric major trauma: a UK multicentre retrospective cohort study
Source: BMJ Open. 2026 Sep 18;16(9):e122447. doi: 10.1136/bmjopen-2026-122447 (PMC13599871; doi:10.1136/bmjopen-2026-122447)
Supplement: Supplementary file 5 [file bmjopen-2026-122447.reviewer_comments.pdf]

PEER REVIEW HISTORY

BMJ Open publishes all reviews undertaken for accepted manuscripts. Reviewers are asked to complete a checklist review form and are provided with free text boxes to elaborate on their assessment. These free text comments are reproduced below.

ARTICLE DETAILS

Title (Provisional)

Admission ionised calcium disturbances and adverse outcomes in paediatric major trauma: a UK multicentre retrospective cohort study

Authors

Hibberd, Owen; Barnard, Edward B G; Karageorgos, Spyridon; Roland, Damian; Harris, Tim; Thomas, Stephen H.; PERUKI, Paediatric Emergency Research in the United Kingdom & Ireland

---

VERSION 1 - REVIEW

**Reviewer**	**1**
**Name**	**Arafa, Mohamed**
**Affiliation**	**Tanta University Faculty of Medicine, Section of Pediatric Surgery, Department of Surgery**
**Date**	**23-Jun-2026**
**COI**	**None**

This multicentre retrospective cohort study addresses an important and understudied topic in paediatric trauma. The inclusion of 13 tertiary centres and a relatively large cohort of major trauma patients strengthens the manuscript. The novel finding regarding the association between admission ionised hypercalcaemia and adverse outcomes is potentially impactful and may generate future hypotheses regarding calcium physiology in injured children.

However, several methodological and interpretative issues should be addressed before publication.

Major Comments

1. Selection Bias Due to Convenience Sampling

The study included only children who had admission ionised calcium measurements performed. The indications for obtaining iCa measurements are not described and are likely influenced by clinician judgment.

Recommendation:

Clarify local indications for obtaining admission iCa measurements.

Discuss the possibility that sicker patients were preferentially tested, introducing selection bias.

Provide, if possible, the proportion of all TARN-eligible major trauma patients who underwent iCa measurement.

2. Potential Confounding by Injury Pattern

AIS data were available for only 39.9% of patients and were not incorporated into the multivariable models.

Given the observed association between hypercalcaemia and isolated head injury, injury location may substantially confound the reported associations.

Recommendation:

Perform sensitivity analyses adjusting for injury pattern in patients with available AIS data.

Discuss more explicitly how incomplete AIS data may have influenced the observed association between iHyperCa and mortality.

3. Interpretation of Hypercalcaemia

The manuscript proposes that hypercalcaemia represents a high-risk phenotype. However, causality cannot be inferred.

The observed association between iHyperCa and mortality may simply reflect severe physiological derangement rather than an independent prognostic biomarker.

Recommendation:

Temper the language throughout the manuscript.

Replace statements implying prognostic utility with statements indicating an association only.

Emphasise that iHyperCa may be a marker of injury severity and physiological stress.

4. Exogenous Calcium Administration

Children with iHyperCa received calcium administration more frequently than normocalcaemic patients.

This finding raises concerns regarding reverse causality and treatment bias.

Recommendation:

Clarify timing of calcium administration relative to calcium measurements.

Report whether calcium administration occurred before or after the index iCa value.

Consider additional analyses excluding patients who received prehospital calcium.

5. Model Development and Overfitting

The manuscript reports multiple logistic regression models with relatively few outcome events, particularly for 24-hour mortality.

For example:

27 deaths at 24 hours

Multiple covariates included

This raises concerns regarding model stability and overfitting.

Recommendation:

Report events-per-variable ratios.

Consider penalised regression methods.

Include model calibration statistics and discrimination measures in the supplementary material.

6. Missing Data Handling

The manuscript states that missing data were excluded from analyses.

Complete-case analysis may introduce bias if missingness was not random.

Recommendation:

Describe patterns of missingness.

Consider multiple imputation or provide justification for complete-case analysis.

Include the percentage of missing data for each regression variable.

7. Definition of Hypercalcaemia

The threshold of iCa ≥1.30 mmol/L appears derived primarily from adult literature.

Paediatric reference ranges vary substantially according to age, particularly during infancy.

Recommendation:

Justify the selected threshold in greater detail.

Discuss age-dependent physiological differences in calcium concentrations.

Consider age-adjusted analyses or sensitivity analyses using age-specific reference intervals.

8. Time from Injury to Calcium Measurement

Children with hypercalcaemia had significantly shorter injury-to-measurement intervals.

This finding may partially explain the higher measured calcium concentrations.

Recommendation:

Include time from injury to sampling as a covariate in multivariable analyses.

Discuss the possibility that calcium levels evolve dynamically after injury.

Minor Comments

1. Typographical and Formatting Issues

“iNormaCa” and “iNormoCa” are used interchangeably. Use one term consistently.

“Amongst” and “among” are used inconsistently.

Several p-values are written as:

p=<0.05

p=<0.001

Use:

$p < 0.05$

$p < 0.001$

throughout.

2. Table 1

The reported median injury-to-calcium measurement time:

116 [151–202] minutes

appears inconsistent because the median cannot be lower than the first quartile.

Please verify these values.

3. Table 2

For hypocalcaemia:

Hypotension:

Table: 44/102 (43.1%)

Text: 27/102 (26.5%)

These values are inconsistent and require correction.

4. Terminology

The manuscript alternates between:

“functional outcome”

“poor functional outcome”

“GOS ≤4”

Consider defining the endpoint once and using consistent terminology.

5. Supplementary Material

The supplementary appendices appear misnumbered:

Main text:

STROBE

Shapiro-Wilk plots

Patient information leaflets

Patient information website

Logistic regression report

Earlier sections refer to different appendix numbers.

Please verify numbering throughout.

6. Figure 1

The map of participating centres would be improved by:

Labelling sites directly;

Providing regional case numbers if available.

**Reviewer**	**2**
**Name**	**Helsloot, dries**
**Affiliation**	**UZ Leuven, Emergency Medicine**
**Date**	**29-Jul-2026**
**COI**	**None**

Reviewer Comments

Thank you for the opportunity to review this manuscript by Hibberd et al.

I would like to congratulate the authors on this important contribution to the existing trauma literature. The paediatric trauma population remains underrepresented in current studies, and conducting research in this population is particularly challenging. The multicentre design and relatively large cohort size are therefore significant strengths of the study.

In this retrospective multicentre analysis, the authors demonstrate that admission ionised hypercalcaemia (iHyperCa), but not ionised hypocalcaemia (iHypoCa), is independently associated with adverse outcomes in paediatric major trauma. These findings contrast with much of the existing literature, which has predominantly focused on hypocalcaemia. The discrepancy may reflect limitations of previous studies, including smaller sample sizes, lack of separate analysis of hypercalcaemia, and important physiological differences between adult and paediatric trauma populations. Indeed, even for the authors these findings may have been somewhat unexpected, particularly given the emphasis on the potential harmful

effects of hypocalcaemia in the patient information leaflet and much of the background literature.

Overall, I believe this is a valuable study addressing a clinically relevant question. However, some aspects of the methodology, interpretation, and presentation would benefit from further clarification.

Major comments

- Line 72: The statement that children may be more vulnerable to calcium disturbances requires further explanation. The physiological basis for this assumption is not sufficiently explained.

- Line 104: The manuscript would benefit from a graphical study flow diagram illustrating patient selection. Specifically, the authors should report: the total number of eligible patients and the number excluded for each exclusion criterion. Where calcium levels available for all eligible patients?

- In addition, several exclusion criteria require clarification. Why were patients who received blood transfusion in the emergency department before calcium measurement excluded, whereas patients who received prehospital blood products were apparently not excluded? Could any patients have received calcium supplementation before the recorded admission calcium measurement? Furthermore, the definition of "treatment at another hospital" should be clarified. Does this include a certain time phrame or predefined treatment criteria?

- Line 112: The rationale for selecting hypotension as a primary outcome deserves further discussion. It is not a “hard” outcome parameter. The variable may be influenced by measurement practices, recording bias, and registry data quality. How can the authors be certain that the registry captures the lowest blood pressure rather than the admission blood pressure? Hypotension is often a late manifestation of shock in children. A more detailed justification for this outcome measure would strengthen the manuscript.

- Line 130: The study appropriately uses age-adjusted definitions for several physiological variables. However, age-specific considerations do not appear to have been applied to ionised calcium levels. Interestingly, most patients with iHyperCa appear to be infants, whereas iHypoCa is more frequently observed in adolescents. This raises the question whether age-related physiological variation in ionised calcium concentrations may partly explain the observed findings. The authors should discuss whether age-specific ionised calcium reference ranges exist and whether alternative analyses accounting for age-dependent calcium physiology were considered. Obtaining accurate blood gas samples in very young children can be challenging, and pre-analytical variability may be more pronounced in this age group?

- Line 131: Please provide additional justification for the selected cut-off values defining ionised hypocalcaemia and hypercalcaemia. Are these thresholds based on paediatric-

specific reference ranges, previous trauma literature, laboratory standards, or expert consensus? Have the authors considered modelling ionised calcium as a continuous variable?

- Table 1: The time from injury to calcium measurement was significantly shorter in the iHyperCa group. Calcium is a dynamic physiological variable that may change considerably during the early post-injury period. Could this difference in sampling time have influenced the observed associations?

- Table 2: The authors report rates of acidosis and hyperlactataemia. Are pCO_2 values available within the dataset? Such information could help differentiate between predominantly respiratory and metabolic causes of acidosis and may aid interpretation of the calcium abnormalities observed.

- Table 4: It is notable that head injury appears to be the predominant injury pattern in the iHyperCa group, whereas thoracic and abdominal (bleeding?) injuries are relatively more prevalent in the iHypoCa group. The discussion references the study by Ciaraglia et al., suggesting that higher rates of hypocalcaemia may be explained by exclusion of isolated TBI and inclusion of more penetrating (bleeding?) injuries. This is an important observation that may also help explain differences between the current cohort and previous literature. I would encourage the authors to expand this discussion further and consider the potential influence of injury pattern and haemorrhage burden on their findings. Can they find any evidence in the literature why head injury would cause a higher incidence of iHyperCa?

- The multicentre design is appropriately highlighted as a strength. However, it may also introduce sources of heterogeneity and bias, including differences in laboratory analysers, variations in calcium measurement techniques, and local trauma management protocols.

- Although the overall cohort is relatively large for a paediatric trauma study and represents an important strength of the manuscript, it should be acknowledged that the final iHypoCa and iHyperCa subgroups remain relatively small. Consequently, some of the reported effect estimates should be interpreted with caution, as analyses on relatively few outcome events within these subgroups may be susceptible to sparse-data bias.

Minor comments

- Line 64: Reference citation appears incorrect ("1 5"). Please revise to either "1-5" or "1,5". There are a few similar errors in the references.

- Table 1: The inclusion of parathyroid and pancreatic injuries is interesting, given their theoretical relationship with calcium homeostasis. However, the absolute numbers are extremely low, and these variables are not discussed further in either the Results or Discussion sections. Either consider removing these variables or provide some commentary regarding their relevance.

- Line 329: The numbers (66/142) are already reported on line 327 and do not need to be repeated.

Finally, I would like to congratulate the authors once again on their considerable efforts in conducting this multicentre study and addressing an important gap in the paediatric trauma literature. The study provides valuable new insights into the relationship between calcium disturbances and outcome following major paediatric trauma, and I hope that the above comments will help further strengthen the manuscript.

VERSION 1 - AUTHOR RESPONSE

Response to Reviewers

Thank you to both reviewers for their valuable and insightful comments. We believe these have significantly strengthened the study and helped improve its clarity, scope, and focus, while also clarifying future research priorities in this field. We are sincerely grateful for all comments received and are happy to make any additional changes as required to further improve the study.

Reviewer 1: Dr. Mohamed Arafa

This multicentre retrospective cohort study addresses an important and understudied topic in paediatric trauma. The inclusion of 13 tertiary centres and a relatively large cohort of major trauma patients strengthens the manuscript. The novel finding regarding the association between admission ionised hypercalcaemia and adverse outcomes is potentially impactful and may generate future hypotheses regarding calcium physiology in injured children.

However, several methodological and interpretative issues should be addressed before publication.

Major Comments

1. Selection Bias Due to Convenience Sampling

The study included only children who had admission ionised calcium measurements performed. The indications for obtaining iCa measurements are not described and are likely influenced by clinician judgment.

Recommendation:

Clarify local indications for obtaining admission iCa measurements.

Discuss the possibility that sicker patients were preferentially tested, introducing selection bias.

Provide, if possible, the proportion of all TARN-eligible major trauma patients who underwent iCa measurement.

- Thank you, we agree. The study included only patients in whom admission ionised calcium was measured, reflecting routine clinical practice rather than protocolised testing. We have clarified that calcium measurement was clinician-directed and therefore subject to selection bias. On reflection this may also be apparent in variation in numbers of patients available at each centre. We have expanded the limitations to acknowledge that clinicians were likely to request calcium measurements in patients perceived to be more severely injured or physiologically unstable. This now reads: 'The multicentre design may have introduced heterogeneity and potential bias through differences in blood gas analysers, local trauma management protocols, and clinician decision-making regarding when to measure ionised calcium. For example, clinicians may have been more likely to request iCa measurements in children perceived to be more severely injured or physiologically unstable, introducing the potential for selection bias'.
- The number of TARN eligible patients was not available, and this has been acknowledged as a limitation. This now reads: 'Since the study occurred in the interim period between TARN closing and the National Major Trauma Registry (NMTR) opening, data availability and patient identification were challenging; this meant that the total number of eligible patients was not available and led to a convenience sample rather than a consecutive sample being obtained, which could have introduced selection and data-availability bias; however, the multi-centre design is likely to have reduced the effect of this.'

2. Potential Confounding by Injury Pattern

AIS data were available for only 39.9% of patients and were not incorporated into the multivariable models.

Given the observed association between hypercalcaemia and isolated head injury, injury location may

substantially confound the reported associations.

Recommendation:

Perform sensitivity analyses adjusting for injury pattern in patients with available AIS data. Discuss more explicitly how incomplete AIS data may have influenced the observed association between iHyperCa and mortality.

- Thank you. Detailed information on AIS regional injuries was available for only 207 cases (40.04% of 517). Since we were aware of the missingness of AIS details during study planning, the *a priori* methodology did not account for injury patterns. This limitation has been emphasised in the revised manuscript, and future studies would benefit from exploring this: 'There was also a significant amount of missing data, particularly related to AIS, prehospital, and additional biochemical data, which reflects the challenges with data collection. Future studies may benefit from synthesising data from UK trauma and prehospital registries which are being established.'
- Given the reviewer's reasonable question about injury patterns, we have calculated our main results incorporating the available AIS data to generate three additional analyses. In the first analysis, we incorporate the AIS for the head region as a scored variable (from 0 to 6). In the second analysis, we incorporated a dichotomous definition for "any head injury" (any head AIS >0). In the third analysis, we classified cases as to whether the head injury was the main injury; this variable was coded as 1 if none of the other AIS regional scores exceeded that of the head AIS. The results from these three analyses are shown in the table below. For the main manuscript we have added the results for 'head injury is most severe injury (no other regional AIS exceeds head AIS)' into the text: 'Head injury as the most severe injury (no other regional AIS exceeded head AIS) was associated with poor functional outcome (OR 2.20 (1.01-4.81)), 24-hour mortality (OR 17.79 (4.05 – 61.53)) and 30-day mortality (OR12.34 (4.82 – 31.58)).'

Analysis	Main reported result for hypercalcemia independent variable, OR (95% CI)	Results incorporating head AIS in three different ways		
		Head AIS as ordinal from 0-6, OR (95% CI)	Any head injury (AIS head >0), dichotomous, OR (95% CI)	Head injury is most severe injury (no other regional AIS exceeds head AIS)
Functional dependence	2.52 (1.45-4.35)	2.31 (1.04-5.11)	2.37 (1.08-5.21)	2.20 (1.01-4.81)
24-hour mortality	19.33 (6.96-53.69)	16.40 (4.16-64.59)	17.2 (4.25-69.64)	15.79 (4.05-61.53)
30-day mortality	9.07 (4.92-16.72)	12.16 (4.78-30.97)	11.73 (4.59-29.98)	12.34 (4.82-31.58)

**Abbreviations:* AIS – Abbreviated Injury Scale (score)

Definitions: Poor functional outcome – Glasgow Outcome Score ≤4

3. Interpretation of Hypercalcaemia

The manuscript proposes that hypercalcaemia represents a high-risk phenotype. However, causality cannot be inferred.

The observed association between iHyperCa and mortality may simply reflect severe physiological derangement rather than an independent prognostic biomarker.

Recommendation:

Temper the language throughout the manuscript.

Replace statements implying prognostic utility with statements indicating an association only.

Emphasise that iHyperCa may be a marker of injury severity and physiological stress.

- Thank you. We agree and have updated the manuscript accordingly, placing further emphasis on iHyperCa as a marker of physiological stress and avoiding the term ‘prognostic’.
- We have rewritten the paragraph on the proposed iHyperCa mechanism, this now reads: ‘The mechanism for iHyperCa is not completely understood. While a small proportion of children with iHyperCa received prehospital calcium supplementation, the majority did not. Sensitivity analyses excluding these patients yielded similar results. Moreover, iHyperCa was measured

early after injury. These findings argue against iHyperCa being just an iatrogenic phenomenon. Recent experimental work has provided important mechanistic insights into calcium disturbances following trauma. Using a controlled porcine polytrauma model with serial physiological sampling, Helsloot et al. demonstrated that iCa undergoes dynamic changes throughout injury and resuscitation, reflecting the combined effects of acid–base disturbances, haemorrhage, tissue hypoperfusion, metabolic derangement, and endocrine responses. There may also be age-related physiological differences that are relevant. In this cohort, iHyperCa was more commonly seen in infants, occurred early after injury, and was associated with acidosis and hyperlactataemia. Plausible mechanisms include acidosis-related iCa shifts, haemoconcentration, severe tissue injury, or age-specific changes in calcium homeostasis. These factors may be more pronounced in infants, owing to their smaller circulating volume, smaller total calcium stores, heightened bone turnover that amplifies inflammatory calcium release, immature parathyroid function, and increased sensitivity to pH-mediated calcium shifts. Greater pre-analytical variability may also have been present in this age group owing to the technical challenges of obtaining blood gas samples in infants and the possibility that clinicians preferentially obtained blood gases in those perceived to be more severely unwell. Sensitivity analyses using age-specific reference ranges did not materially alter the findings. However, because the dataset used broad age categories rather than exact ages, more detailed exploration of age-related physiological variation in iCa concentrations was not possible. In particular, neonates could not be distinguished from older infants, despite the neonatal period theoretically being associated with more pronounced differences in calcium homeostasis. Future studies should explore these age-specific effects using more granular age data and larger sample sizes. Overall, causality cannot be inferred from this data, and the observed association suggests that iHyperCa may be an area for further research and mechanistic studies in paediatric trauma.'

- We have emphasised that causality cannot be inferred from this data: 'However, causality cannot be inferred from this data, and the observed association suggests that iHyperCa may be an area for further research and mechanistic studies in paediatric trauma.'

4. Exogenous Calcium Administration

Children with iHyperCa received calcium administration more frequently than normocalcaemic patients.

This finding raises concerns regarding reverse causality and treatment bias.

Recommendation:

Clarify timing of calcium administration relative to calcium measurements.

Report whether calcium administration occurred before or after the index iCa value.

Consider additional analyses excluding patients who received prehospital calcium.

- Thank you. The following has also been added to the limitations: 'Additionally, outside the prehospital setting, the timing of exogenous calcium administration was not recorded and therefore could not be confirmed as occurring before or after the index ionised calcium measurement. However, patients receiving emergency department blood transfusion before calcium measurement were excluded, and empiric calcium administration before either blood transfusion or ionised calcium measurement would not be routine clinical practice.'
- While documentation of prehospital therapy was available for only part of the dataset (*n* = 328), calcium treatment was initiated prehospital in only six cases. With exclusion of those six cases, repetition of key findings did not suggest any substantive change in findings, as evidenced by the table below (which has been added to the supplemental appendices). The table reproduces the methods of the main results (including primary reporting of Firth penalised regression results for the 24-hour mortality endpoint).

**Analysis**	**Main reported result for hypercalcemia independent variable, OR (95% CI)**	**Result excluding cases with prehospital calcium, OR (95% CI)**
Poor functional outcome	2.52 (1.45-4.35)	2.53 (1.45-4.43)
24-hour mortality	19.33 (6.96-53.69)	22.27 (7.60-65.24)

30-day mortality	9.07 (4.92-16.72)	9.90 (5.31-18.48)

**Abbreviations:* CI – confidence interval, OR – odds ratio

Definitions: Poor functional outcome – Glasgow Outcome Score ≤4

5. Model Development and Overfitting

The manuscript reports multiple logistic regression models with relatively few outcome events, particularly for 24-hour mortality.

For example:

27 deaths at 24 hours

Multiple covariates included

This raises concerns regarding model stability and overfitting.

Recommendation:

Report events-per-variable ratios.

Consider penalised regression methods.

Include model calibration statistics and discrimination measures in the supplementary material.

- We agree that some of the regression models may be susceptible to low events-per-variable ratios and the risk of partial or complete separation. During manuscript preparation, we had originally cited the work of van Smeden et al., supporting our decision-making surrounding employment of Firth penalised logistic regression (van Smeden M, de Groot JA, Moons KGM, et al. No rationale for 1 variable per 10 events criterion for binary logistic regression analysis. BMC Med Res Methodol. 2016;16:163). This reference was subsequently removed during efforts to reduce the manuscript length. It has now been reinstated, and additional methodological references have also been included to further justify the statistical approach employed (detailed in the final section of this response document).

- We agree. We used Firth-penalised logistic regression when we encountered convergence difficulties or when we found separation in the Stata modelling reports. This was the reason that the 24-hour mortality results were primarily reported as Firth results. Even using the most liberal events:variable, and keeping in mind that events:variable is calculated based on the covariates in the initial (not final, parsimonious) model, we lacked sufficient numbers to provide definitive results. Our event numbers were 27 (5.22% of 517) for 24-hour mortality, 62 (11.99% of 517) for 30-day mortality, and 152 (31.02% of 490 with non-missingness) for dependent status; these would support mortality assessments with only 2-3 variables for the 24-hour endpoint and 5-6 variables for the 30-day endpoint. Since we could not assume that age band had a linear association with any endpoint, we assessed age as an indexed variable. This means that assessment of age accounted for four variables (*k* - 1 where *k* is five age groups).
- The events:variable conundrum would be a critical shortcoming if we were attempting a highly precise effect estimate, but our study's message was a simpler reporting of a signal of associations (between hypercalcemia and the various endpoints) that warrant further evaluation. Because of this, and because the results were reported using Firth where complete separation was identified, we believe it defensible to report our results with conservative and preliminary interpretation. The table below shows results for our main findings (hypercalcemia and endpoints of mortality and functional outcome) and the similarity of findings from Firth regression.

**Analysis**	**Main reported result for hypercalcemia independent variable, OR (95% CI)**	**Result obtained using Firth logistic regression, OR (95% CI)**
Poor functional outcome	2.52 (1.45-4.35)	2.50 (1.45-4.31)
24-hour mortality	19.33 (6.96-53.69)	(Main result reported Firth logistic regression finding)
30-day mortality	9.07 (4.92-16.72)	8.80 (4.81-16.11)

**Abbreviations:* CI – confidence interval, OR – odds ratio

Definitions: Poor functional outcome – Glasgow Outcome Score ≤4

- The Supplement now includes discrimination and calibration information. The table below summarises the findings.

**Analysis**	**Receiver operator characteristic curve area (95% confidence interval)**	**Calibration-in-the-large**	**Calibration slope**
Poor functional outcome	0.73 (0.68-0.77)	0.00 (-0.21 to 0.20)	1.01 (0.74-1.28)
24-hour mortality	0.78 (0.68-0.88)	-0.11 (-0.53 to 0.31)	1.04 (0.71-1.37)
30-day mortality	0.78 (0.72-0.85)	-0.02 (-0.31 to 0.27)	1.01 (0.76-1.26)

**Definitions*: Poor functional outcome – Glasgow Outcome Score ≤4

Calibration plot for hypercalcemia and functional outcome

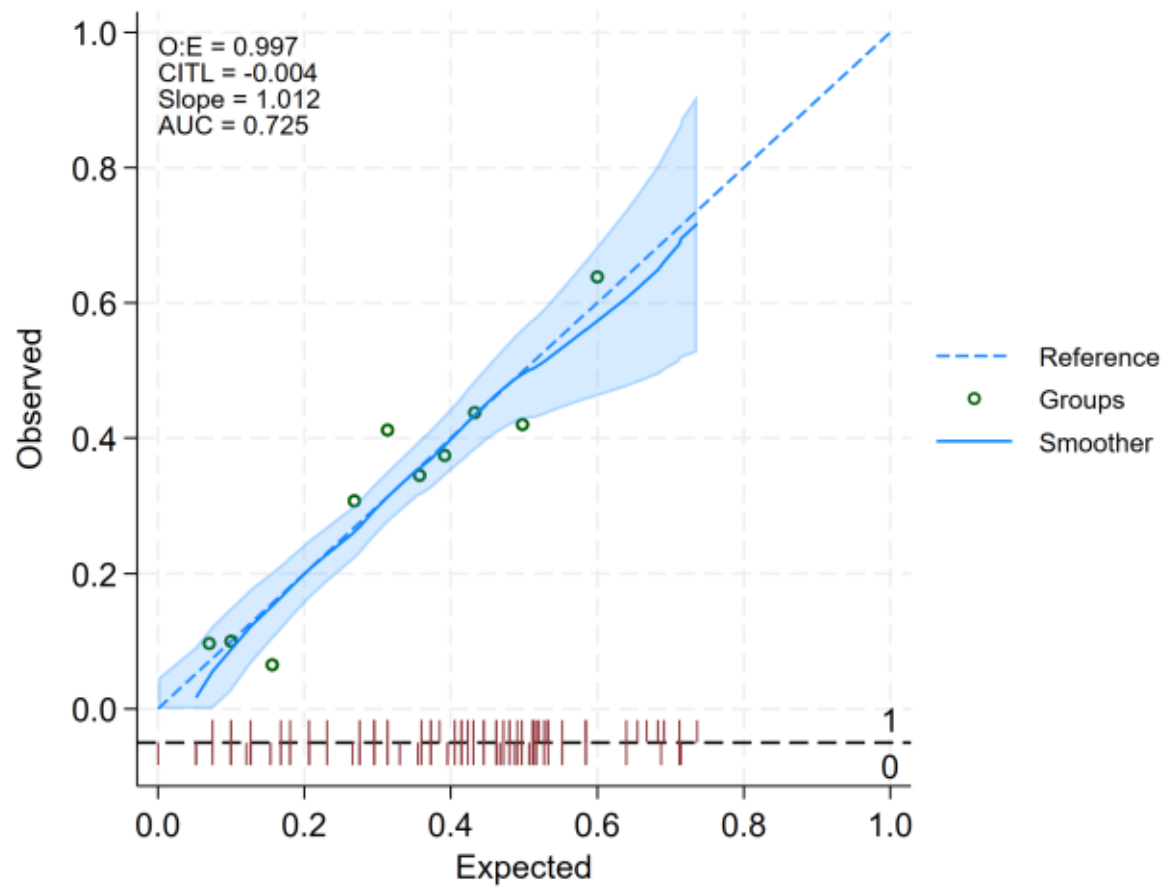

Calibration plot for hypercalcemia and 24-hour mortality

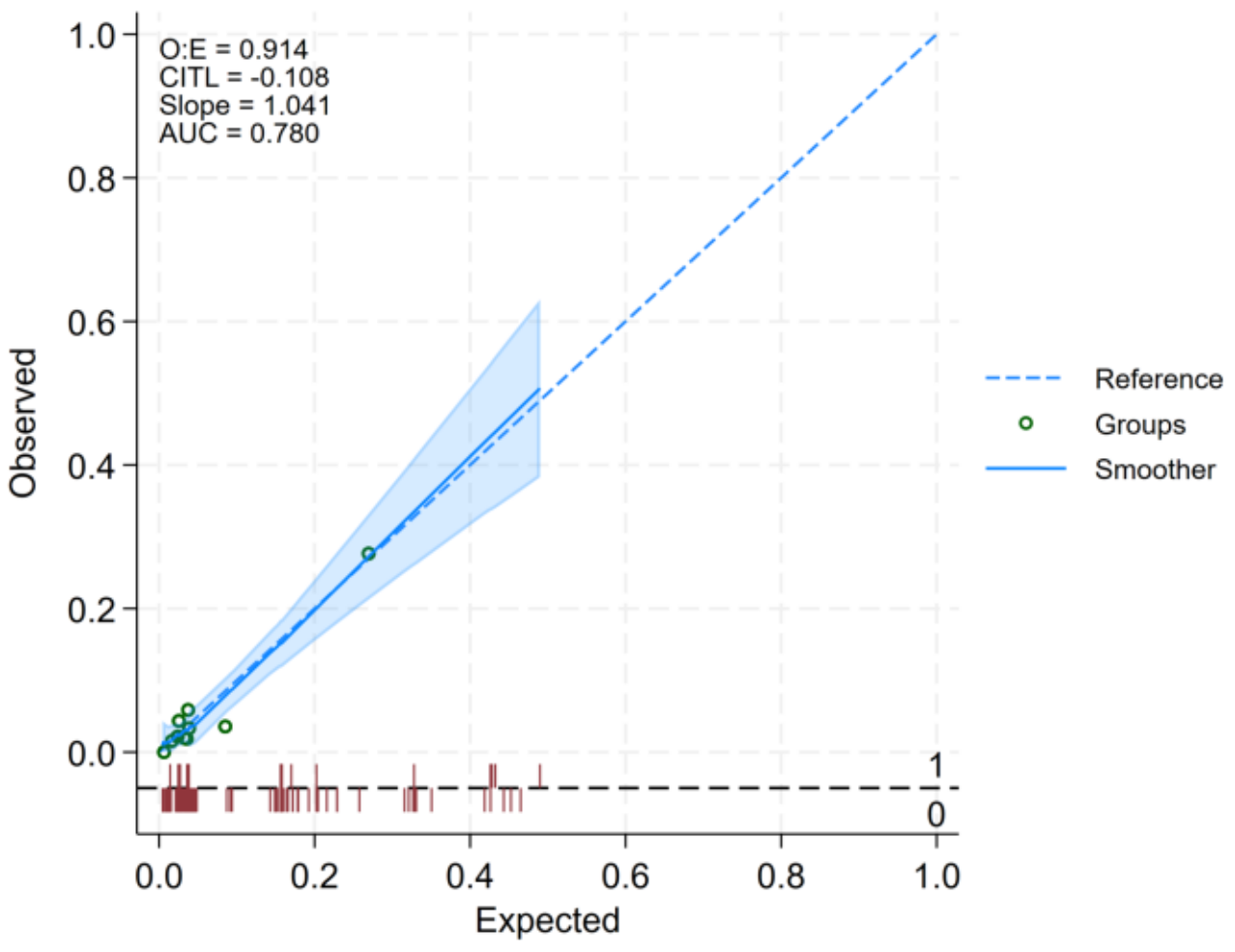

Calibration plot for hypercalcemia and 30-day mortality

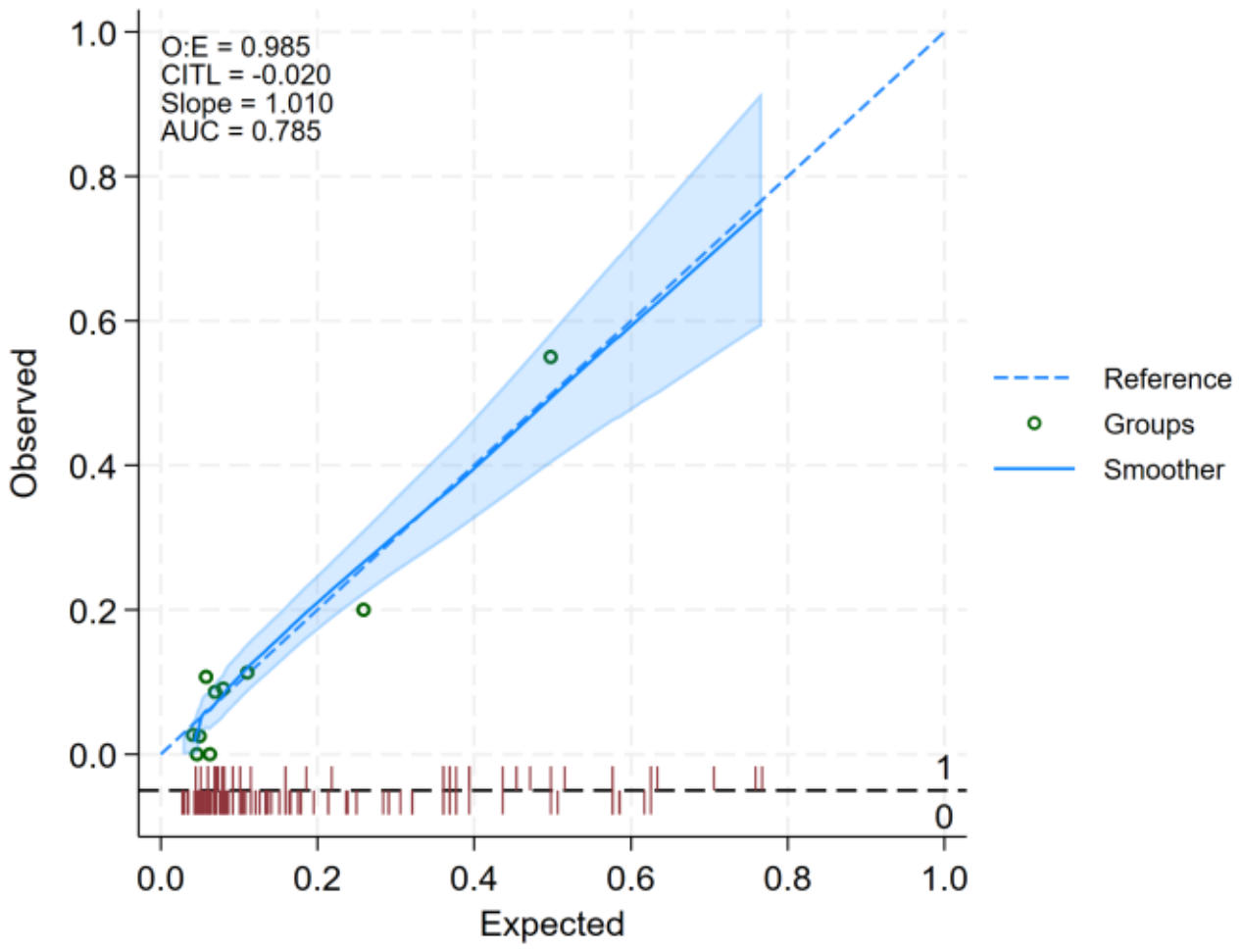

6. Missing Data Handling

The manuscript states that missing data were excluded from analyses.

Complete-case analysis may introduce bias if missingness was not random.

Recommendation:

Describe patterns of missingness.

Consider multiple imputation or provide justification for complete-case analysis.

Include the percentage of missing data for each regression variable.

- Thank you. Out of an overall *n* = 517 patients, the two mortality models (for 24-hour and 30-day time frames) reported on 515 cases, indicating low missingness rates that resulted in exclusion of only 2 cases. This missingness rate was the result of low missingness amongst the two models' covariates. For the 24-hour mortality model the covariates were age band (0% missingness) and ISS (missing in 2 of 517 cases, 0.39%). For the 30-day mortality model the covariates were ISS (missingness 0.39%) and trauma mechanism (penetrating or blunt, missing in 0 cases). There were too few missing data to assess patterns of missingness for these endpoints, and we judged that the low missingness rates (well under 5%) did not support the added complexity of multiple imputation by chained equation (MICE).
- The model for poor functional outcome utilised a smaller portion of the overall dataset: of the overall *n* of 517, the functional outcome utilised 488 patients. However, nearly all of the model's missingness was due to missing functional outcome in 27 cases (5.22% of 517). With removal of these cases due to lack of ascertainment of the functional status, there were only two other cases dropped due to covariate (ISS) missingness. Since we do not execute MICE in either the primary independent variable (hypercalcemia, with 0% missingness) or the outcome (functional status), we judged there was no role for MICE for these data. We have added a sensitivity analysis to the supplemental appendices that shows the results of replacing all missing functional outcome cases as either having poor functional outcome or good functional outcome; results are shown along with the original results in the table below and indicate little substantive change in the effect estimate (with 95% CIs that overlap with each other and the main results, and which exclude the null value).
- Information on missing data is provided in the footnotes of each table, and this has now also been added to the regression model table.

**Main independent variable**	**Main results OR (95% CI) for hypercalcemia independent variable in estimation sample *n* = 488**	**Replacement of cases missing functional outcome with either favorable or non-favorable status**	
		**Replacement with unfavorable status, OR (95% CI)**	**Replacement with favorable status, OR (95% CI)**

Hypercalcemia	2.52 (1.45-4.35)	2.82 (1.68-4.71)	2.49 (1.45-4.29)

**Abbreviations:* CI – confidence interval, GOS – Glasgow Outcome Core, OR – odds ratio

7. Definition of Hypercalcaemia

The threshold of iCa ≥1.30 mmol/L appears derived primarily from adult literature.

Paediatric reference ranges vary substantially according to age, particularly during infancy.

Recommendation:

Justify the selected threshold in greater detail.

Discuss age-dependent physiological differences in calcium concentrations.

Consider age-adjusted analyses or sensitivity analyses using age-specific reference intervals.

- Thank you for this important comment. We selected the ionised calcium thresholds used in the primary analysis to facilitate comparison with the existing adult trauma literature, where the majority of published studies have employed similar thresholds. We agree, however, that paediatric age-specific reference ranges are less well established and remain heterogeneous across the literature. This lack of consensus is reflected in both our previous systematic review and a recent national survey of clinician practice, which demonstrated substantial variation in the definitions and management of calcium disturbances in paediatric trauma.
- To address this concern, we have added a sensitivity analysis using age-specific ionised calcium thresholds to the Supplementary Appendix. The purpose of this analysis was to determine whether the observed associations between iHyperCa and adverse outcomes persisted when paediatric age-based definitions were applied. Reassuringly, the findings remained robust, with no substantive attenuation of the effect estimates towards the null. Application of the age-specific thresholds reclassified 27 patients from the hypercalcaemia group to the normocalcaemia group; of these, 19 were infants, six

were toddlers, and one each was of school age and adolescence. The results of this sensitivity analysis are presented in the table below.

**Analysis**	**Main reported result for hypercalcemia independent variable, OR (95% CI)**	**Result obtained when utilizing age-based varying reference for hypercalcemia, OR (95% CI)**
Poor functional outcome	2.52 (1.45-4.35)	3.39 (1.72-6.67)
24-hour mortality	19.33 (6.96-53.69)	27.65 (10.17-75.23)
30-day mortality	9.07 (4.92-16.72)	12.63 (6.28-25.40)

**Abbreviations:* CI – confidence interval, OR – odds ratio

Definitions: Poor functional outcome – Glasgow Outcome Score ≤4

- Exploring this further, we have also added the following to the main text:
  - ‘Sensitivity analyses using age-specific thresholds produced similar results, with no meaningful movement of the effect estimates towards the null (supplemental appendix 3).’
  - These findings reflect the wider literature, where there remains no consensus regarding the ionised calcium thresholds that define clinically relevant calcium disturbances, and evidence supporting age-specific reference ranges in children is limited.
- We have added the following to the discussion exploring age-related differences in more detail:
  - ‘These factors may be more pronounced in infants, owing to their smaller circulating volume, smaller total calcium stores, heightened bone turnover that amplifies inflammatory calcium release, immature parathyroid function, and increased sensitivity to pH-mediated calcium shifts.[1 2 51] Greater pre-analytical variability may also have been present in this age group owing to the technical challenges of obtaining blood gas samples in infants and the possibility that clinicians preferentially obtained blood gases in those perceived to be more severely unwell.’
  - ‘Sensitivity analyses using age-specific reference ranges did not materially alter the findings. However, because the dataset used broad age categories rather than exact ages,

more detailed exploration of age-related physiological variation in iCa concentrations was not possible. In particular, neonates could not be distinguished from older infants, despite the neonatal period theoretically being associated with more pronounced differences in calcium homeostasis. Future studies should explore these age-specific effects using more granular age data.'

- Additionally, to avoid ambiguity, the age categories have been revised to clearly represent continuous age ranges rather than appearing to contain gaps and therefore more accurately reflecting the data. They are now reported as 0–1 years, 1–3 years, 3–5 years, 5–12 years, and 12–16 years.

8. Time from Injury to Calcium Measurement

Children with hypercalcaemia had significantly shorter injury-to-measurement intervals.

This finding may partially explain the higher measured calcium concentrations.

Recommendation:

Include time from injury to sampling as a covariate in multivariable analyses.

Discuss the possibility that calcium levels evolve dynamically after injury.

- We agree that time from injury to calcium assessment may be important. We did not include this variable because of its missingness (missing in 83 of 517 cases, 16.05%). With a *caveat* that the modeling of time from injury to calcium assessment is not optimal – due to both missingness and also possibility of non-linear relationships for which did not evaluate – we evaluated the three hypercalcemia endpoints in models that incorporated time from injury to calcium assessment. In none of the three models was the time from injury to calcium statistically significant, although the evaluation for the poor functional outcome approached significance (time from injury to calcium assessment $p = .069$). For the two mortality endpoints the time from injury to calcium assessments were both >.9. As shown in the table below, there was no substantive change in hypercalcemia effect estimates for any of the three outcomes; the directionality of movement when incorporating time from injury to calcium

assessment was away from the null. This table has been added to the main text: 'Incorporating time from injury to calcium measurement into the regression models did not result in any substantive change in the effect estimates for the three outcomes significantly associated with ionised hypercalcaemia. In each case, the effect estimates shifted slightly further away from the null (Table 4).'

**Analysis**	**Main reported result for hypercalcemia independent variable, OR (95% CI)**	**Result obtained when adding time from injury to calcium assessment as an additional covariate, OR (95% CI)**
Poor functional outcome	2.52 (1.45-4.35)	2.65 (1.48-4.78)
24-hour mortality	19.33 (6.96-53.69)	19.47 (6.95-54.58)
30-day mortality	9.07 (4.92-16.72)	10.85 (5.62-20.93)

**Abbreviations:* CI – confidence interval, OR – odds ratio

Definitions: Poor functional outcome – Glasgow Outcome Score ≤4

- In exploring the mechanism of calcium disturbances in the discussion we have added the following: 'Recent experimental work has provided important mechanistic insights into calcium disturbances following trauma. Using a controlled porcine polytrauma model with serial physiological sampling, Helsloot et al. demonstrated that iCa undergoes dynamic changes throughout injury and resuscitation, reflecting the combined effects of acid–base disturbances, haemorrhage, tissue hypoperfusion, metabolic derangement, and endocrine responses.'

Minor Comments

1. Typographical and Formatting Issues

"iNormaCa" and "iNormoCa" are used interchangeably. Use one term consistently.

- Thank you. We greatly appreciate this attention to detail and have updated this to be iNormoCa throughout.

"Amongst" and "among" are used inconsistently.

- Thank you. This has been updated to ‘among’ throughout.

Several p-values are written as:

p=<0.05

p=<0.001

Use:

$p < 0.05$

$p < 0.001$

throughout.

- Thank you. This has been updated accordingly.

2. Table 1

The reported median injury-to-calcium measurement time: 116 [151–202] minutes appears inconsistent because the median cannot be lower than the first quartile.

Please verify these values.

- Thank you. This has been corrected.

3. Table 2

For hypocalcaemia:

Hypotension:

Table: 44/102 (43.1%)

Text: 27/102 (26.5%)

These values are inconsistent and require correction.

- Thank you. This typo has been corrected to 44/102 (43.1%) in the text.

4. Terminology

The manuscript alternates between:

“functional outcome”

“poor functional outcome”

“GOS ≤4”

Consider defining the endpoint once and using consistent terminology.

- Thank you. We have now used ‘poor functional outcome’ throughout and defined this as Glasgow Outcome Score ≤4 in the methods and in relevant tables.

5. Supplementary Material

The supplementary appendices appear misnumbered:

Main text:

STROBE

Shapiro-Wilk plots

Patient information leaflets

Patient information website

Logistic regression report

Earlier sections refer to different appendix numbers.

Please verify numbering throughout.

- Thank you. This has been updated accordingly.

6. Figure 1

The map of participating centres would be improved by:

Labelling sites directly;

Providing regional case numbers if available.

- Thank you. This is an excellent suggestion, and we have updated the figure accordingly.

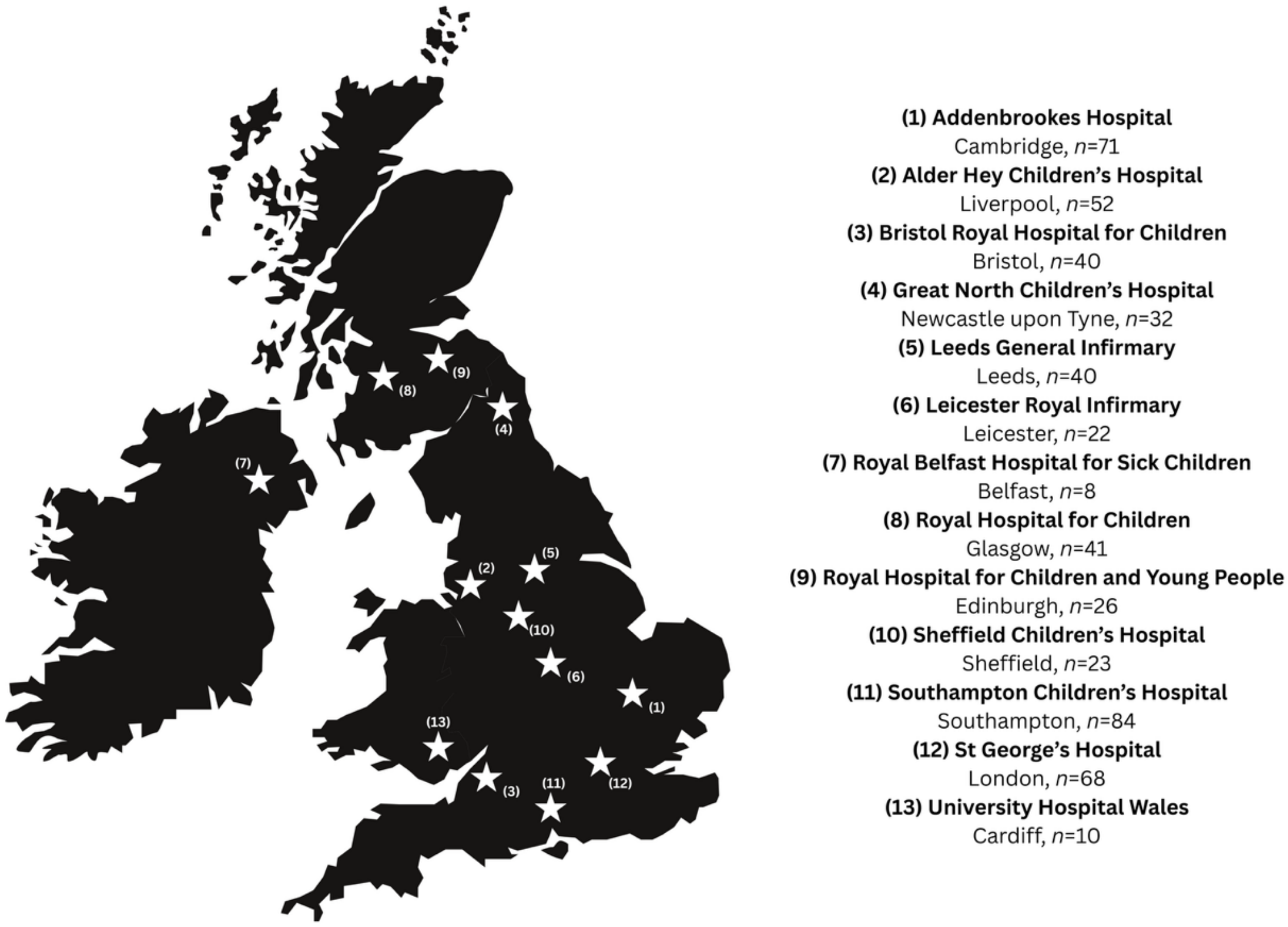

Reviewer 2: Dr. Dries Helsloot

Thank you for the opportunity to review this manuscript by Hibberd et al.

I would like to congratulate the authors on this important contribution to the existing trauma literature. The paediatric trauma population remains underrepresented in current studies, and conducting research in this population is particularly challenging. The multicentre design and relatively large cohort size are therefore significant strengths of the study.

In this retrospective multicentre analysis, the authors demonstrate that admission ionised hypercalcaemia (iHyperCa), but not ionised hypocalcaemia (iHypoCa), is independently associated with adverse outcomes in paediatric major trauma. These findings contrast with much of the existing literature, which has predominantly focused on hypocalcaemia. The discrepancy may reflect limitations of previous studies, including smaller sample sizes, lack of separate analysis of hypercalcaemia, and important physiological differences between adult and paediatric trauma

populations. Indeed, even for the authors these findings may have been somewhat unexpected, particularly given the emphasis on the potential harmful effects of hypocalcaemia in the patient information leaflet and much of the background literature.

Overall, I believe this is a valuable study addressing a clinically relevant question. However, some aspects of the methodology, interpretation, and presentation would benefit from further clarification.

Major comments

Line 72: The statement that children may be more vulnerable to calcium disturbances requires further explanation. The physiological basis for this assumption is not sufficiently explained.

- Thank you. We have update this to read: 'Children may also be particularly vulnerable to calcium disturbances compared with adults because of differences in haemodynamic response and physiological reserves, different injury mechanisms and patterns, higher bone turnover, smaller total calcium stores, and developmental haemostasis referring to the age-related maturation of the coagulation system, whereby the concentrations and function of coagulation factors, anticoagulant proteins, fibrinolytic pathways, and platelet activity differ between neonates, infants, children, and adults.'
- Additional references have been added to support this (detailed at the end of this response document).

Line 104: The manuscript would benefit from a graphical study flow diagram illustrating patient selection. Specifically, the authors should report: the total number of eligible patients and the number excluded for each exclusion criterion. Were calcium levels available for all eligible patients?

- Thank you. Unfortunately, the total number of eligible patients was not available, nor did sites provide numbers of excluded patients. We recognise this as a significant limitation and have updated this to read: 'Since the study occurred in the interim period between TARN closing and the National Major Trauma Registry (NMTR) opening, data availability and patient

identification were challenging; this meant that the total number of eligible patients was not available and led to a convenience sample rather than a consecutive sample being obtained, which could have introduced selection and data-availability bias; however, the multi-centre design is likely to have reduced the effect of this.'

In addition, several exclusion criteria require clarification. Why were patients who received blood transfusion in the emergency department before calcium measurement excluded, whereas patients who received prehospital blood products were apparently not excluded? Could any patients have received calcium supplementation before the recorded admission calcium measurement? Furthermore, the definition of "treatment at another hospital" should be clarified. Does this include a certain time frame or predefined treatment criteria?

- Thank you. Please see lines 167 to 184 of this response document.
- Patients who received blood product transfusion in the emergency department before ionised calcium measurement were excluded because citrate-containing blood products may confound admission iCa levels. We did not apply the same exclusion to prehospital blood product recipients because complete prehospital data were unavailable for all patients. Among those with available prehospital data, only a small proportion received prehospital blood products or calcium supplementation. However, excluding only those with documented prehospital blood product transfusion/calcium supplementation would have risked introducing additional bias, as some patients without prehospital data may also have received these treatments. We have clarified this in the manuscript and acknowledge that prospective studies with complete prehospital data collection are needed to address this limitation.
- This is now more clearly described in the limitations as: 'The inclusion of patients who received prehospital blood product transfusions or exogenous calcium may also be considered a limitation. Although excluding this group might have reduced confounding, access to prehospital data was not consistently available across sites. Given that all UK HEMS services carry blood products, applying this as an exclusion criterion would have risked inadvertently

including such patients due to incomplete data capture.[15] Importantly, only a small proportion of the overall cohort were documented as receiving prehospital blood product transfusion, and blood products were administered to patients in both the iHypoCa and iHyperCa groups, reducing the likelihood of bias in one direction. Additionally, outside the prehospital setting, the timing of exogenous calcium administration was not recorded and therefore could not be confirmed as occurring before or after the index ionised calcium measurement. However, patients receiving emergency department blood transfusion before calcium measurement were excluded, and empiric calcium administration before either blood transfusion or ionised calcium measurement would not be routine clinical practice.'

- We have added the following to clarify the exclusion criteria around secondary transfers: 'Patients transferred from another hospital following initial treatment for the same injury were excluded because transfer-related delays and incomplete access to data from the referring hospital could have confounded the analyses.'

Line 112: The rationale for selecting hypotension as a primary outcome deserves further discussion. It is not a "hard" outcome parameter. The variable may be influenced by measurement practices, recording bias, and registry data quality. How can the authors be certain that the registry captures the lowest blood pressure rather than the admission blood pressure? Hypotension is often a late manifestation of shock in children. A more detailed justification for this outcome measure would strengthen the manuscript.

- Thank you. We agree, however, hypotension was selected over mortality due to it being a clinically relevant marker of shock, that is actionable in the emergency department, whereas mortality is rarer in paediatric trauma.
- We have also included shock-index paediatric-adjusted (SIPA) in the outcome data, however, particularly in the context of trauma, tachycardia is a less sensitive paediatric marker for shock due to confounders such as pain and distress.

- Although some data were available through local trauma offices (such as age categories, mechanism of injury, length of stay, and mortality data), a large amount of data required challenging manual extraction by local collaborators, details of which are included in the methods section. The lowest blood pressure in the ED was manually extracted by local collaborators following extensive review of patient electronic medical records.
- We have added the following to the methods: 'This outcome was selected because of its hypothesised mechanistic relationship with calcium disturbances and its clinical relevance in the emergency department management of paediatric trauma, where mortality is relatively uncommon.'

Line 130: The study appropriately uses age-adjusted definitions for several physiological variables. However, age-specific considerations do not appear to have been applied to ionised calcium levels. Interestingly, most patients with iHyperCa appear to be infants, whereas iHypoCa is more frequently observed in adolescents. This raises the question whether age-related physiological variation in ionised calcium concentrations may partly explain the observed findings. The authors should discuss whether age-specific ionised calcium reference ranges exist and whether alternative analyses accounting for age-dependent calcium physiology were considered. Obtaining accurate blood gas samples in very young children can be challenging, and pre-analytical variability may be more pronounced in this age group?

- Thank you. Please see the above on lines 292-338 of this response document.
- We have also added the following to the discussion: 'Greater pre-analytical variability may also have been present in this age group owing to the technical challenges of obtaining blood gas samples in infants and the possibility that clinicians preferentially obtained blood gases in those perceived to be more severely unwell.'

Line 131: Please provide additional justification for the selected cut-off values defining ionised hypocalcaemia and hypercalcaemia. Are these thresholds based on paediatric-specific reference

ranges, previous trauma literature, laboratory standards, or expert consensus? Have the authors considered modelling ionised calcium as a continuous variable?

- Thank you. Please see the above on lines 292-338 of this response document.
- Our a priori plan was to analyse calcium in categories, as this seemed more useful as a general guide, but we agree that the next steps should include evaluation of calcium as a continuous variable. Such analyses may be quite detailed, as the data suggest that there will be a non-linear relationship (as described in your studies too). We feel that a larger sample size would be required for this, requiring either additional studies or individual-participant data meta-analysis of existing data.

Table 1: The time from injury to calcium measurement was significantly shorter in the iHyperCa group. Calcium is a dynamic physiological variable that may change considerably during the early post-injury period. Could this difference in sampling time have influenced the observed associations?

- Thank you. Please see the above lines 347-372 in this response document.

Table 2: The authors report rates of acidosis and hyperlactataemia. Are pCO_2 values available within the dataset? Such information could help differentiate between predominantly respiratory and metabolic causes of acidosis and may aid interpretation of the calcium abnormalities observed.

- Unfortunately, $PaCO_2$ was not requested from sites, and therefore we are unable to analyse this. Future studies may benefit from including this.
- We have added the following to the discussion: 'Additionally, given the complex physiological mechanisms underlying calcium disturbances demonstrated in Helsloot et al's. experimental study, future studies should incorporate a broader panel of biochemical markers. Alongside iCa, this should include arterial carbon dioxide ($PaCO_2$), which is routinely available from blood gas analysis, as well as potassium, phosphate, and magnesium, to better characterise the metabolic and physiological response to trauma.'

Table 4: It is notable that head injury appears to be the predominant injury pattern in the iHyperCa group, whereas thoracic and abdominal (bleeding?) injuries are relatively more prevalent in the iHypoCa group. The discussion references the study by Ciaraglia et al., suggesting that higher rates of hypocalcaemia may be explained by exclusion of isolated TBI and inclusion of more penetrating (bleeding?) injuries. This is an important observation that may also help explain differences between the current cohort and previous literature. I would encourage the authors to expand this discussion further and consider the potential influence of injury pattern and haemorrhage burden on their findings. Can they find any evidence in the literature why head injury would cause a higher incidence of iHyperCa?

- Thank you. Please see the above lines 85-110 in this response document.
- We have also rewritten the discussion around Ciaraglia et al's paper: 'The higher incidence of severe iHypoCa reported by Ciaraglia et al. may be explained by differences in the study population, including the exclusion of isolated head injuries and a higher proportion of penetrating trauma (38/142, 26.7%), which is likely to have been associated with a greater haemorrhagic burden'.

The multicentre design is appropriately highlighted as a strength. However, it may also introduce sources of heterogeneity and bias, including differences in laboratory analysers, variations in calcium measurement techniques, and local trauma management protocols.

- Thank you. We have added this to the limitations and have also cited our recently published survey, which demonstrates variations in protocols: 'The multicentre design may have introduced heterogeneity and potential bias through differences in blood gas analysers, local trauma management protocols, and clinician decision-making regarding when to measure ionised calcium. For example, clinicians may have been more likely to request iCa measurements in children perceived to be more severely injured or physiologically unstable, introducing the potential for selection bias'.
- We have also introduced this survey in the introduction to provide further rationale for this study: 'Moreover, a recent international survey of practices around the measurement and

replacement of calcium in paediatric major trauma demonstrated substantial variation in both the measurement and replacement of iCa, highlighting a lack of consensus and a need for further evidence.'

Although the overall cohort is relatively large for a paediatric trauma study and represents an important strength of the manuscript, it should be acknowledged that the final iHypoCa and iHyperCa subgroups remain relatively small. Consequently, some of the reported effect estimates should be interpreted with caution, as analyses on relatively few outcome events within these subgroups may be susceptible to sparse-data bias.

- Thank you. We agree. The Firth penalised regression partially mitigates, but does not eliminate, this problem.
- Thank you. We have added this to the limitations: 'Similarly, the number of patients with calcium disturbances was a small proportion, and the reported effect estimates should be interpreted with caution, as the results may be susceptible to sparse-data bias.'

Minor comments

Line 64: Reference citation appears incorrect ("1 5"). Please revise to either "1-5" or "1,5". There are a few similar errors in the references.

- We apologise. This appears to be the BMJ style selected on the EndNote plug-in for Word. If required, we can manually update these.

Table 1: The inclusion of parathyroid and pancreatic injuries is interesting, given their theoretical relationship with calcium homeostasis. However, the absolute numbers are extremely low, and these variables are not discussed further in either the Results or Discussion sections. Either consider removing these variables or provide some commentary regarding their relevance.

- Thank you. We agree that these variables were not appropriately placed within Table 1. They have therefore been removed from the table and incorporated into the injured area subgroup

analysis, with the relevant findings now reported in the Results text. This now reads: 'Pancreatic (9/517, 1.7%) and parathyroid injuries (1/517, 0.2%) were rare and occurred too infrequently to permit meaningful statistical analysis.'

Line 329: The numbers (66/142) are already reported on line 327 and do not need to be repeated.

- Thank you. We have removed the repetition and appreciate your attention to detail.

Finally, I would like to congratulate the authors once again on their considerable efforts in conducting this multicentre study and addressing an important gap in the paediatric trauma literature. The study provides valuable new insights into the relationship between calcium disturbances and outcome following major paediatric trauma, and I hope that the above comments will help further strengthen the manuscript.

Additional references

This is a rapidly evolving field of research, highlighting the growing recognition of the clinical importance of calcium disturbances following trauma.

Since the manuscript was originally submitted, several relevant studies have been published that further explore the physiology of trauma-induced calcium disturbances, current paediatric clinical practice, and priorities for future trauma research.

Recent publications which inform the study:

- Becker ER, Pratt CG, Whitrock JN, et al. Early Hypercalcemia Is Associated With Mortality in Critically Ill, Transfused Trauma Patients. J Surg Res. 2025;315:991-996. doi:10.1016/j.jss.2025.10.009
- Helsloot D. Ionized calcium: A dynamic physiological marker in trauma and resuscitation?. J Trauma Acute Care Surg. Published online July 17, 2026. doi:10.1097/TA.0000000000005137

- Helsloot D, Neyrinck A, Pottel H, et al. Kinetics and dynamics of ionized calcium in a porcine major trauma model. *Journal of Trauma and Acute Care Surgery* 2026:10.1097/TA.0000000000004997. doi: 10.1097/ta.0000000000004997
- Hibberd O, Ranaweera M, Karageorgos S, et al. Survey of practices around the measurement and replacement of calcium in paediatric major trauma. *BMJ Paediatrics Open* 2026;10(1):e004588. doi: 10.1136/bmjpo-2026-004588
- Schauer SG, Nicholson SE, Rizzo JA, et al. Initial Calcium Derangements in Major Trauma and Outcomes. JAMA Netw Open. 2026;9(2):e260083. doi:10.1001/jamanetworkopen.2026.0083
- Sloane C, Waterfield T, Evans J, et al. Refreshing the paediatric emergency medicine research priorities across the UK and Ireland. *Emerg Med J* 2026 doi: 10.1136/emermed-2025-215836 [published Online First: 20260601]
- Wilson AM, Borgman MA, Cox CS, et al. Increased mortality in children with severe trauma and bleeding: Hypotheses and methods to reduce the risk. Transfusion. 2026;66 Suppl 1:S337-S344. doi:10.1111/trf.70109

Additional references added to support physiological rationale:

- Cho WI, Yu HW, Chung HR, et al. Clinical and laboratory characteristics of neonatal hypocalcemia. *Ann Pediatr Endocrinol Metab* 2015;20(2):86-91. doi: 10.6065/apem.2015.20.2.86 [published Online First: 20150630]
- McNeilly JD, Boal R, Shaikh MG, et al. Frequency and aetiology of hypercalcaemia. *Arch Dis Child* 2016;101(4):344-7. doi: 10.1136/archdischild-2015-309029 [published Online First: 20160222]
- Omosule CL, Holmes V, Jasek C, et al. Pediatric ionized calcium reference intervals from archived radiometer data. *Clin Biochem* 2022;104:13-18. doi: 10.1016/j.clinbiochem.2022.03.001 [published Online First: 20220311]
- Rodríguez Soriano J. Neonatal hypercalcemia. *J Nephrol* 2003;16(4):606-8.

- Toulon P, Berruyer M, Brionne-François M, et al. Age dependency for coagulation parameters in paediatric populations. Results of a multicentre study aimed at defining the age-specific reference ranges. *Thromb Haemost* 2016;116(1):9-16. doi: 10.1160/th15-12-0964 [published Online First: 20160317]
- Toulon P. Developmental hemostasis: laboratory and clinical implications. *Int J Lab Hematol* 2016;38 Suppl 1:66-77. doi: 10.1111/ijlh.12531
- Vuralli D. Clinical Approach to Hypocalcemia in Newborn Period and Infancy: Who Should Be Treated? *Int J Pediatr* 2019;2019:4318075. doi: 10.1155/2019/4318075 [published Online First: 20190619]

Additional references added to support statistical methodology:

- Miller ME, Hui SL, Tierney WM. Validation techniques for logistic regression models. *Stat Med*. 1991;10(8):1213-1226. doi:10.1002/sim.4780100805
- Steyerberg EW, Vickers AJ, Cook NR, et al. Assessing the performance of prediction models: a framework for traditional and novel measures. *Epidemiology*. 2010;21(1):128-138. doi:10.1097/EDE.0b013e3181c30fb2
- van Smeden M, de Groot JA, Moons KG, et al. No rationale for 1 variable per 10 events criterion for binary logistic regression analysis. *BMC Med Res Methodol*. 2016;16(1):163. Published 2016 Nov 24. doi:10.1186/s12874-016-0267-3

VERSION 2 - REVIEW

**Reviewer**	**2**
**Name**	**Helsloot, dries**
**Affiliation**	**UZ Leuven, Emergency Medicine**
**Date**	**23-Aug-2026**
**COI**	

Thank you for the opportunity to review this revised manuscript. I would like to acknowledge the considerable effort the authors have invested in addressing the reviewers' comments. The manuscript has improved considerably and now provides a much clearer description of both the study methodology and its limitations.

As acknowledged by the authors, this study inevitably faces several challenges inherent to retrospective multicentre research in a paediatric trauma population, including limitations in data availability and the potential for bias. However, the authors have appropriately acknowledged these limitations and have adopted a more balanced interpretation of their findings.

Overall, this study provides valuable new insights into calcium disturbances in paediatric major trauma, an area where evidence remains limited and difficult to generate.

Finally, I would like to congratulate the authors once more on their efforts in conducting this multicentre study, addressing the reviewers' comments in a thoughtful and comprehensive manner, and integrating recent insights into the revised manuscript. I believe the manuscript is suitable for publication in its current form.
